# Structural Design and Energy and Environmental Applications of Hydrogen‐Bonded Organic Frameworks: A Systematic Review

**DOI:** 10.1002/advs.202400101

**Published:** 2024-04-22

**Authors:** Xiaoming Liu, Guangli Liu, Tao Fu, Keren Ding, Jinrui Guo, Zhenran Wang, Wei Xia, Huayuan Shangguan

**Affiliations:** ^1^ Department of Resources and Environment Moutai Institute Renhuai 564507 China; ^2^ College of Environmental Sciences and Engineering Peking University Beijing 100871 China; ^3^ AgResearch Ruakura Research Centre Hamilton 3240 New Zealand; ^4^ College of Environmental Science and Engineering Tongji University Shanghai 200092 China; ^5^ School of Environmental Science and Engineering Southwest Jiaotong University Chengdu 611756 China; ^6^ Key Laboratory of Urban Environment and Health Institute of Urban Environment Chinese Academy of Sciences Xiamen 361021 China

**Keywords:** assembly design, coupling perspectives, energy applications, environmental governance, Hydrogen‐bonded organic frameworks

## Abstract

Hydrogen‐bonded organic frameworks (HOFs) are emerging porous materials that show high structural flexibility, mild synthetic conditions, good solution processability, easy healing and regeneration, and good recyclability. Although these properties give them many potential multifunctional applications, their frameworks are unstable due to the presence of only weak and reversible hydrogen bonds. In this work, the development history and synthesis methods of HOFs are reviewed, and categorize their structural design concepts and strategies to improve their stability. More importantly, due to the significant potential of the latest HOF‐related research for addressing energy and environmental issues, this work discusses the latest advances in the methods of energy storage and conversion, energy substance generation and isolation, environmental detection and isolation, degradation and transformation, and biological applications. Furthermore, a discussion of the coupling orientation of HOF in the cross‐cutting fields of energy and environment is presented for the first time. Finally, current challenges, opportunities, and strategies for the development of HOFs to advance their energy and environmental applications are discussed.

## Graphical abstract

In this work, the unique properties, structural designs, and synthesis methods of hydrogen‐bonded organic frameworks (HOFs) are systematically classified, summarized, and discussed based on recent progress. Further, the environmental and energy applications of HOFs are comprehensively delineated, and the cross‐disciplinary opportunities and challenges of HOFs in future directions are proposed.

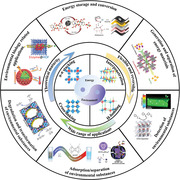

## 1 Introduction

Porous crystalline materials, such as metal‐organic frameworks (MOFs) and covalent organic frameworks (COFs), have made substantial progress in terms of their synthesis and applications.^[^ ^1^ ^]^ MOFs consist of metal ions (clusters) connected to organic ligands by ligand bonds.^[^ ^2^ ^]^ The refinement of secondary building units and reticular chemistry has facilitated their design and synthetic development.^[^ ^3^ ^]^ Similarly, COFs are assembled from organic building blocks connected by covalent bonds and have similar rapid advantages as MOFs.^[^ ^1^, ^4^ ^]^ Due to the pre‐designable organic units and topologies, MOFs, and COFs demonstrate tunable framework structures, pore sizes, and specific surface areas^[^ ^5^ ^]^ and have made progress in practical applications.^[^ ^4^, ^6^ ^]^

**Figure 1:**
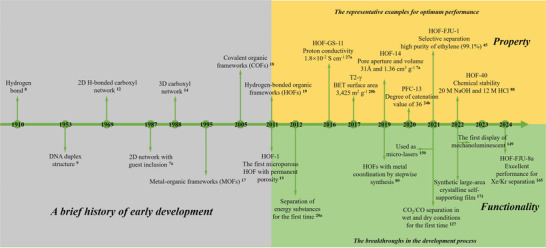
Timeline of the milestones and representative research advances during the development of hydrogen‐bonded organic frameworks (HOFs).

Hydrogen‐bonded organic frameworks (HOFs) consist of organic or metal‐organic architectural units connected by hydrogen bonds and are gradually developing into an attractive new type of porous crystalline materials (**Figure** **1**).^[^ ^7^ ^]^ Hydrogen bonding theory provides the fundamental basis for the construction of HOFs. In 1912, Moore and Winmill first discussed the alkalinity of trimethylammonium hydroxide in terms of intermolecular interactions known as hydrogen bonds.^[^ ^8^ ^]^ The concept and role of hydrogen bonding in water were elucidated by Latimer and Rodebush in 1920, and hydrogen bonding in solid organic molecules was subsequently reported.^[^ ^9^ ^]^ In 1953, Watson and Crick reported that base complementary pairs based on hydrogen bonding played a key role in the formation of stable DNA structures.^[^ ^10^ ^]^ The International Union of Pure and Applied Chemistry (IUPAC) defines hydrogen bonding as attractive interactions between a hydrogen atom from a molecular fragment and an electronegative atom or atomic group from another molecular fragment.^[^ ^11^ ^]^ The bonding energy and directionality of most hydrogen bonds are inferior to those of the ligand bonding in MOFs or the covalent bonding of COFs,^[^ ^7c^ ^]^ giving them labile structures and complex molecular assemblies. However, this precludes the rational design and feasibility of constructing hydrogen‐bonded organic framework materials by using previous approaches.

Some pioneering work addressed the challenge of constructing HOF networks. Duchamp and Marsh attempted to construct a hexagonal honeycomb crystalline hydrogen‐bonded network using trimesic acids (TMAs) as the building blocks.^[^ ^12^ ^]^ In 1987, Herbstein et al. obtained a hydrogen‐bonded network by using template molecules (e.g., long‐chained alkanes and primary alcohols) in a guest‐encapsulated hydrogen‐bond network with non‐chained TMAs.^[^ ^13^ ^]^ In 1988, Ermer reported the crystal structure of adamantane‐1,3,5,7‐tetracarboxylic acid (ADTA) with a five‐fold interpenetrating 3D dense network rhombic topology.^[^ ^14^ ^]^ In 1991, a series of hydrogen‐bonded constructed HOFs was reported by Wuest's research team.^[^ ^15^ ^]^ The concept of constructing porous hydrogen‐bonded networks predates Robson's work on the coordination polymer, tetracyanotetraphenylmethane,^[^ ^16^ ^]^ as well as the terminology of MOFs^[^ ^17^ ^]^ and COFs,^[^ ^18^ ^]^ which were coined by Yaghi in 1995 and 2005, respectively. Although the concepts of constructing HOFs and MOFs were proposed almost contemporaneously, HOFs received less attention, causing their development to significantly lag behind that of MOFs. This was mainly due to the weak, flexible, and poorly orientated hydrogen bonds, which made it difficult to precisely synthesize stable HOF structures. The frameworks also tended to collapse when the solvent was removed from the pores in early synthetic attempts. Furthermore, early scientists did not focus on the porous structure of HOFs, resulting in extremely slow development. In 2011, Chen et al. proposed the first precise definition of HOFs and elucidated for the first time the potential applications of permanent pores in HOF‐1 for gas separation (ethylene/ethane), marking a new beginning in this field.^[^ ^19^ ^]^ Oppel's group prepared HOFs with a rigid backbone and ultra‐high void content in 2012 and 2017, achieving >2700, and 3400 m^2^ g^−1^, respectively.^[^ ^20^ ^]^ These pioneering works established an understanding of the porosity and high porosity of HOFs and their applications and stimulated interest and motivation in the development of new multifunctional HOFs. Since then, a new era of development has begun, leading to the formalization of HOFs as a new and unique class of porous framework materials favored by researchers.

The properties of HOFs give them unique potential material formation and multifunctional applications.^[^ ^7b,c^ ^]^ HOFs can be synthesized under mild and reversible conditions, are formed after removing volatile solvents, and can dissociate into their original building frameworks in some solvents. Therefore, HOFs are processable,^[^ ^21^ ^]^ reproducible, and recyclable.^[^ ^21^, ^22^ ^]^ The flexibility of HOFs' structure allows them to exhibit adaptive dynamics during guest encapsulation and also provides a good platform for molecular recognition studies. The metal‐free nature of HOFs endows them with better biocompatibility and lower cytotoxicity, giving them potential biological applications. In addition, the convenience of adding substituents to organic building blocks has facilitated the development of multifunctional HOFs.^[^ ^7b^ ^]^ However, the weak bonding energy and poor directionality of hydrogen bonding have limited the development of HOFs.^[^ ^23^ ^]^ The structure of HOFs depends on the solvents and synthesis conditions and is susceptible to external factors and forces.^[^ ^24^ ^]^ Currently, several structural design strategies have been proposed to enhance the stability and the intermolecular forces of HOFs, such as the introduction of multiple hydrogen bonds,^[^ ^20^, ^25^ ^]^ π–π stacking,^[^ ^26^ ^]^ charge‐assisted hydrogen bonding,^[^ ^27^ ^]^ interpenetration,^[^ ^21^, ^28^ ^]^ and chemical cross–linking.^[^ ^5a^ ^]^ Many stable HOFs with permanent pores have been reported and may serve as precursors for fabricating multifunctional composites. In addition, high‐throughput theoretical calculations have advanced the strategy shift of HOFs from experimental attempts to map their properties to rational construction schemes. Day et al. and other research groups have applied advanced theoretical computational strategies to the structural design and functional prediction of HOFs.^[^ ^20^, ^25^ ^]^ These pioneering works have stimulated research in this field and led to the rapid development of HOFs in terms of both structural design and applications. Due to these efforts, HOFs show great potential applications in fields such as gas storage and separation, molecular recognition, conductive and optical applications, multiphase catalysis, and biomedicine.^[^ ^7^, ^29^ ^]^

**Scheme 1:**
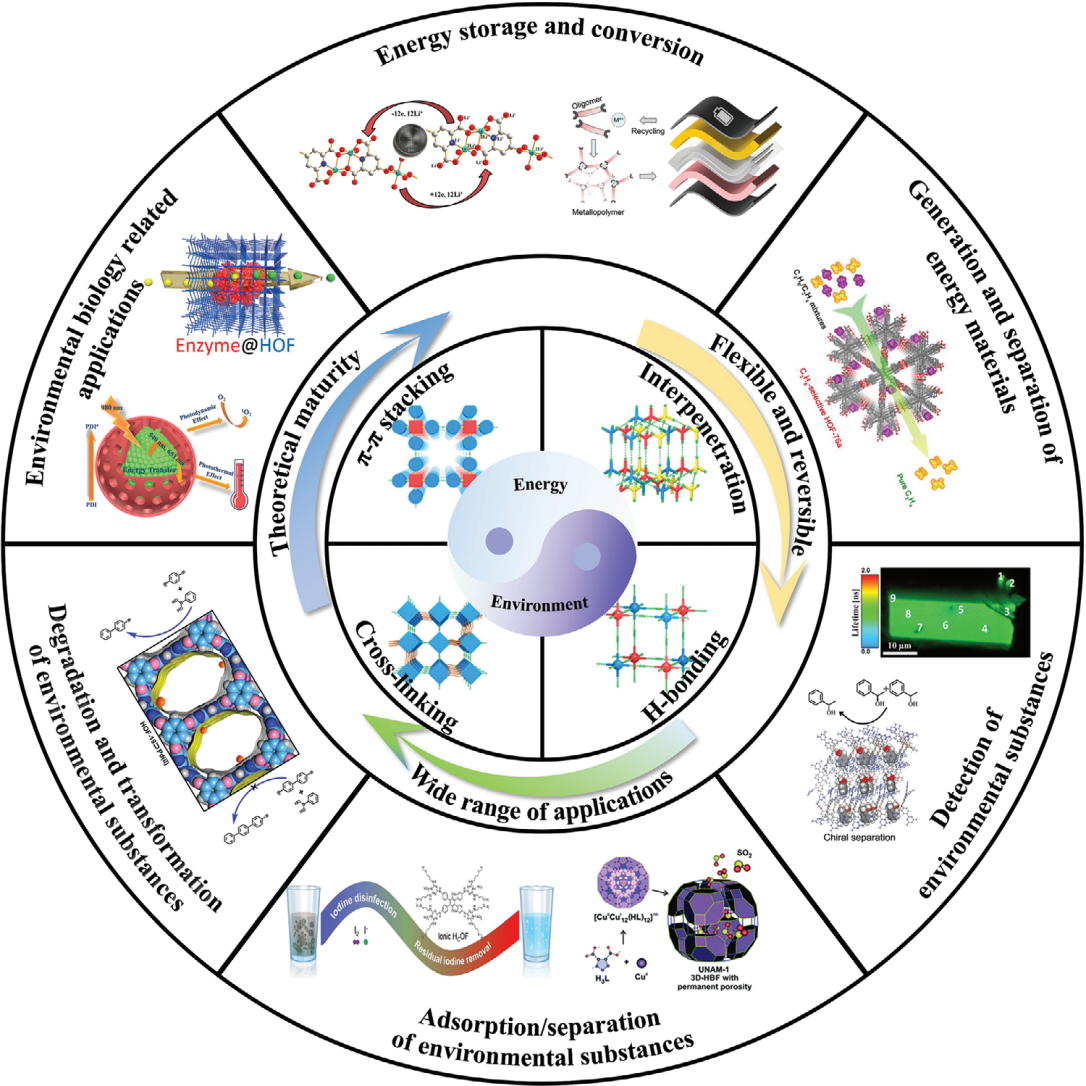
Schematic illustration of structural designs and energy and environmental applications of HOFs. Reproduced with permission.^[^ ^5a^ ^]^ Copyright 2022, American Chemical Society. Reproduced with permission.^[^ ^7b^ ^]^ Copyright 2022, Elsevier. Reproduced with permission.^[^ ^27c^ ^]^ Copyright 2019, American Chemical Society. Reproduced with permission.^[^ ^54^ ^]^ Copyright 2021, American Chemical Society. Reproduced with permission.^[^ ^79a^ ^]^ Copyright 2019, American Chemical Society. Reproduced with permission.^[^ ^95c^ ^]^ Copyright 2016, American Chemical Society. Reproduced with permission.^[^ ^102^ ^]^ Copyright 2021, American Chemical Society. Reproduced with permission.^[^ ^138^ ^]^ Copyright 2020, American Chemical Society. Reproduced with permission.^[^ ^161^ ^]^ Copyright 2019, Royal Society of Chemistry. Reproduced with permission.^[^ ^168^ ^]^ Copyright 2022, Wiley‐VCH. Reproduced with permission.^[^ ^194^ ^]^ Copyright 2021, Wiley‐VCH.

The future development and prospects of HOFs will see a new dawn with structural and functionalization development. However, few review articles have comprehensively outlined the design strategies, synthetic methods, and application prospects of HOFs. Therefore, this review predominantly concentrates on the design principles, regulatory strategies, and synthetic methods of HOFs. It underscores the interconnected relationship between design theory, material structure, and practical applications. The aim is to provide a comprehensive reference for the development of new structures and applications of HOFs. Especially in the context of carbon neutrality and energy scarcity, there have been few reports of progress in the environmental applications of HOFs and energy‐related fields and their intercrossed applications. We also focus on the use of HOFs for energy and environmental applications, including energy storage and conversion, energy substance generation and separation, environmental pollutant detection, environmental pollutant degradation and conversion, and environmental biological applications (**Scheme** **1**).^[^ ^7^, ^29^ ^]^ Based on the intersection of these fields, the optimal design, challenges, and opportunities of HOFs for coupled applications are proposed. This review promotes the rapid development of HOFs in environmental and energy‐related fields and the maturation of HOFs from multiple perspectives.

## 2 Structural Design and Regulation of HOFs

Hydrogen bonds in HOFs are a unique force that confers tunability, polymorphism, and flexibility during structural design. Understanding the fundamental modes of construction and properties of HOFs is the basis for accurately designing and constructing functional HOFs. The weak hydrogen‐bonding interactions make it challenging to construct permanently pore‐stable HOFs, so understanding the assembly design principles, common synthesis strategies, and typical use cases of highly stable HOFs will be beneficial for tailoring HOFs to the requirements of different applications.

### 2.1 Structural Design and Characteristics of HOFs

#### 2.1.1 Hydrogen Bond Construction and Design in HOFs

**Figure 2:**
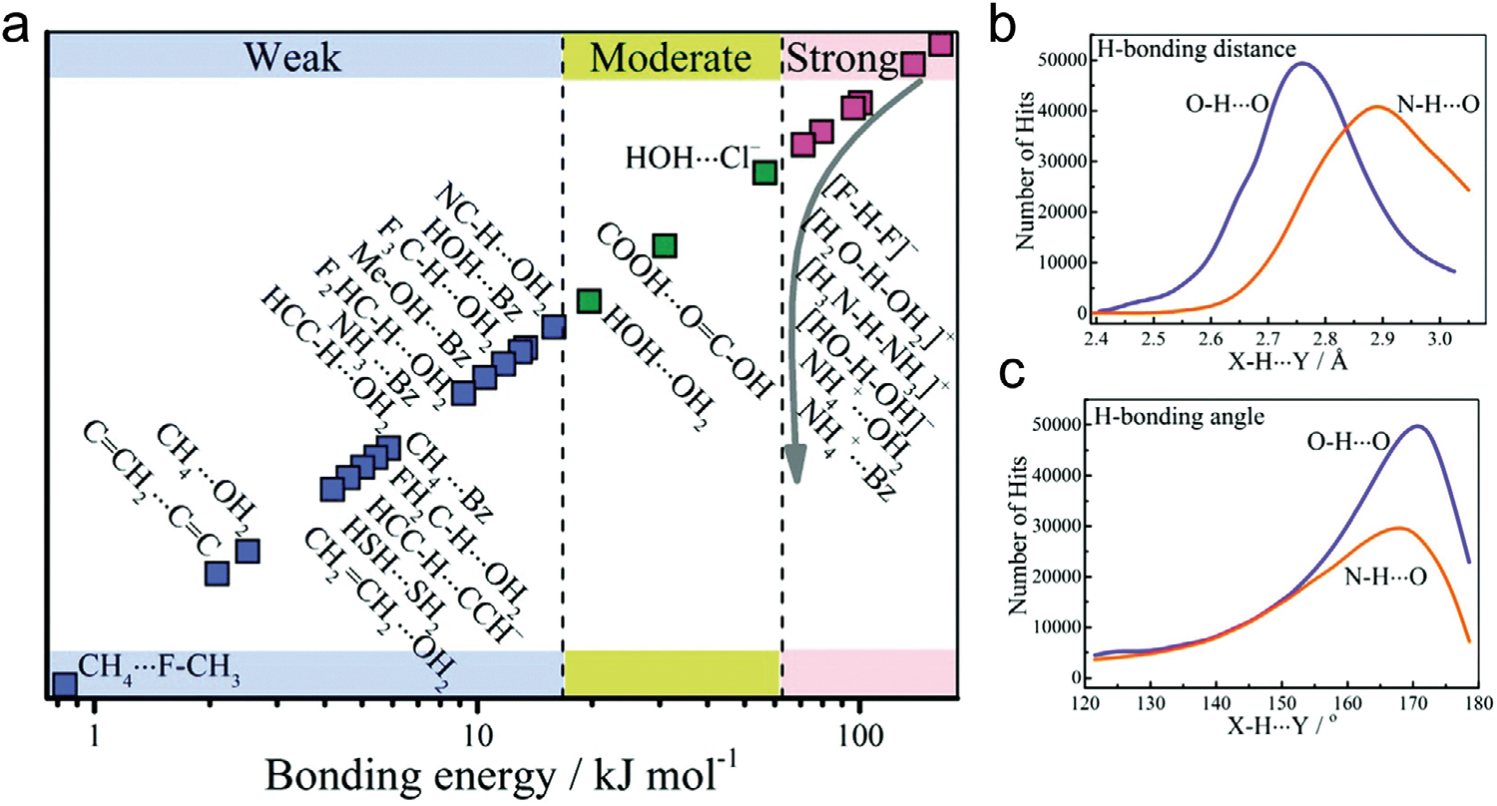
Basic properties of hydrogen bonding. a) Various types of hydrogen bonds (the positions and colors are non‐quantitative bond energy scales). Distributions of b) O─H⋯O and N─H⋯O bond length and c) corresponding directionality. Reproduced with permission.^[^ ^7c^ ^]^ Copyright 2019, Royal Society of Chemistry.

HOFs are mainly connected through intermolecular hydrogen bonds. Therefore, it is essential to first introduce the fundamental properties of hydrogen bonds to understand the construction of hydrogen bonds in HOFs. Precisely, hydrogen bonds are defined as electrostatic interactions between a hydrogen atom attached to a strongly electronegative atom or group (N, O, F, etc.) and another neighboring atom with a pair of lone pairs of electrons (N, O, F, etc.).^[^ ^11^ ^]^ According to their bond energy, they can be divided into weak hydrogen bonds, medium hydrogen bonds, and strong hydrogen bonds (**Figure** **2**a).^[^ ^7c^ ^]^ Strong hydrogen bonds with high directionality mainly exist as intermolecular O/N─H⋯O/N interactions with average bonding distances of 2.77 and 2.88 Å for O─H⋯O and N─H⋯O, respectively (Figure 2b).^[^ ^7c^ ^]^ A shorter bond distance indicates a stronger hydrogen bond and better orientation, i.e., the bond angles are centered at 171° and 168° (Figure 2c). Strong hydrogen bonds have high bond energies and can form stable HOFs, while the use of weak hydrogen bonds can result in polycrystallinity, flexibility, or framework collapse. The bond length and directionality of weak hydrogen bonds are poor, leading to the formation of different polycrystalline forms, so they are usually not chosen for constructing HOFs.

Reticular chemistry is used to design periodic network materials from well‐defined building block units through strong connecting bonds,^[^ ^30^ ^]^ while the weak interactions, high flexibility, and low directionality of hydrogen bonds make it difficult to obtain specific strong connections when synthesizing HOFs. The instability and fragility of the connections, as well as their interpenetration and tendonization, greatly hinder the use of reticular chemistry to design HOFs.^[^ ^31^ ^]^ As described above, the major challenge to building HOFs lies in the implementation of permanent porosity, rigidity, and stability of the framework, which are hampered by fragile and weak hydrogen bonds.

**Figure 3:**
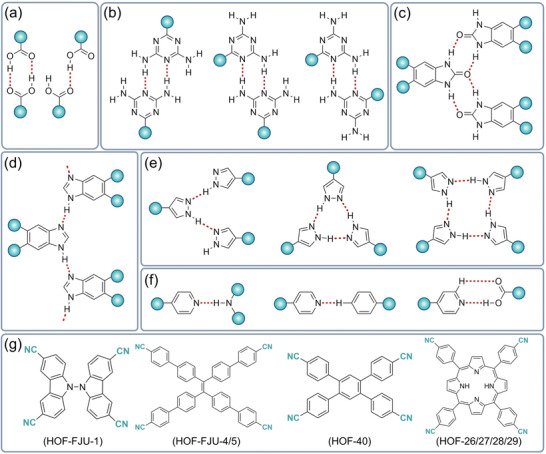
General hydrogen bonding patterns and molecular building blocks in HOFs. a) Carboxylate, b) Diaminotriazine, c) Urea, d) Imidazole, e) Pyrazole, f) Pyridine, and g) Cyanide. The red dotted line and cyan circle represent hydrogen bonds and substituents, respectively. Reproduced with permission.^[^ ^30^ ^]^ Copyright 2022, Wiley‐VCH.

Supramolecular synthetic species formed by multiple hydrogen bond pairs, some of which are highly orientated and exhibit specific spatial geometries, have recently been explored to serve as stable linking units for reticulated chemically designed HOFs. As shown in **Figure** **3** and Table S1 (Supporting Information), carboxyl dimers, 2,4‐diaminotriazine (DAT) dimers, cyclic pyrazole trimers, benzimidazolone chains, amidinocarboxylates, guanidinium sulphonates, and cyanide are some of the most common supramolecular synthetics for building HOFs.^[^ ^30^ ^]^ Carboxy dimers are the most common building blocks used to connect molecules via O─H⋯O hydrogen bonds between molecules to generate HOFs (Figure 3a). DAT can be used to construct novel HOFs in three different bonding modes (Figure 3b). Building blocks containing urea moieties can be used to generate ribbon structures via N─H⋯O hydrogen bonds to produce ribbon structures (Figure 3c). A similar linear system can be formed by imidazole with fewer hydrogen bonds implying a weaker skeleton (Figure 3d). Pyrazoles can be constructed with hydrogen‐bonded synthetics having either linear, triangular, or tetragonal geometries (Figure 3e). Pyridines can be synthesized via N─H⋯N, C─H⋯N, and O─H⋯N hydrogen bonds to form hydrogen‐bonded networks (Figure 3f). Cyanine‐containing units can form different types of HOFs by changing the skeleton monomer (Figure 3g). Recently, many HOFs have been constructed from cyanide‐containing units, including HOF‐26/27/28/29, mainly using interactions such as C─H⋯N, C─H⋯π, and π–π interactions (Figure S1, Supporting Information).

**Figure 4:**
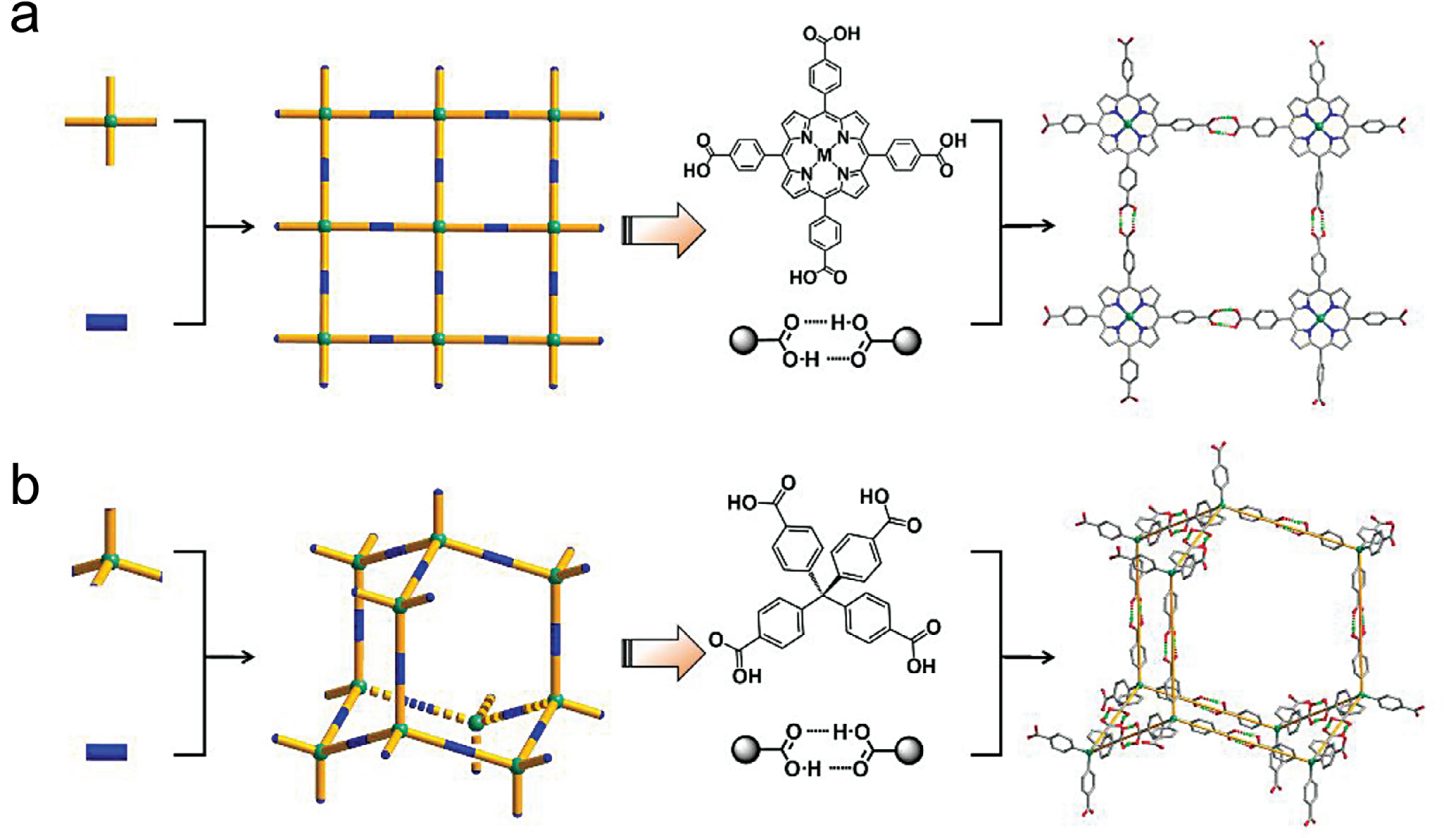
Examples showing the reticular chemistry used to design HOFs with a) *sql* and b) *dia* topologies, respectively. Reproduced with permission.^[^ ^29b^ ^]^ Copyright, 2022 American Chemical Society.

High‐throughput computational methods have also been used to screen and predict HOF structures, where the probability of obtaining a structure is related to its lattice energy. Stacking arrangements with porous structures can be identified by comparing the lattice energies of the simulated structures. **Figure** **4** shows a representative example of HOFs designed by reticular chemistry.^[^ ^29b^ ^]^ To date, libraries of stable HOFs with permanent porosity have been designed and synthesized by synergistically selecting the above supramolecular synthons and rigid constructors. Some HOFs are very stable and retain their structures well, even under extreme conditions.

Cooper and co‐workers combined computational crystal structure prediction (CSP) with high‐throughput crystallographic screening to predict and isolate novel porous polycrystals of TMA and ADTA, thus validating the feasibility of their method.^[^ ^25^ ^]^ Day et al. combined CSP with property prediction to create an energy‐structure‐function (ESF) diagram for predicting possible structures and their properties.^[^ ^20b^ ^]^ Using this strategy, three new porous polycrystals of triheptene imidazolidinone T2 derivatives (i.e., T2‐β, T2‐γ, and T2‐δ), as well as an ultra‐low‐density form of T2E (i.e., T2E‐α) were discovered. The calculated structures and properties were in good agreement with the experimental results. CSP was recently used to predict and construct a cage‐based low‐density mesoporous HOF with a BET surface area of 1750 m^3^ g^−1^.^[^ ^32^ ^]^ This was the first non‐penetrating mesoporous 3D HOF with permanent porosity. Pyzer‐Knapp et al. utilized a parallel Bayesian optimization technique to selectively obtain energy and property data at a very low computational cost to obtain the same level of insight, improve ESP, and accelerate the computational discovery of porous HOFs.^[^ ^33^ ^]^

#### 2.1.2 Intrinsic Characteristics and Regulation of HOFs

##### Adjustable Pore Size and Surface Ratio

A HOF's pore shape and size can be tuned by selecting different building blocks. The same number of hydrogen bond donors and acceptors in an organic moiety can form intrinsic hydrogen bond building blocks such as dimers and trimers.^[^ ^7c^ ^]^ The selection of a suitable hydrogen bond building block is essential for constructing highly porous HOFs because they can transfer their geometry to the HOF network when they are combined with organic frameworks. In this way, they form extended frameworks with different pore sizes and topologies.^[^ ^34^ ^]^

**Figure 5:**
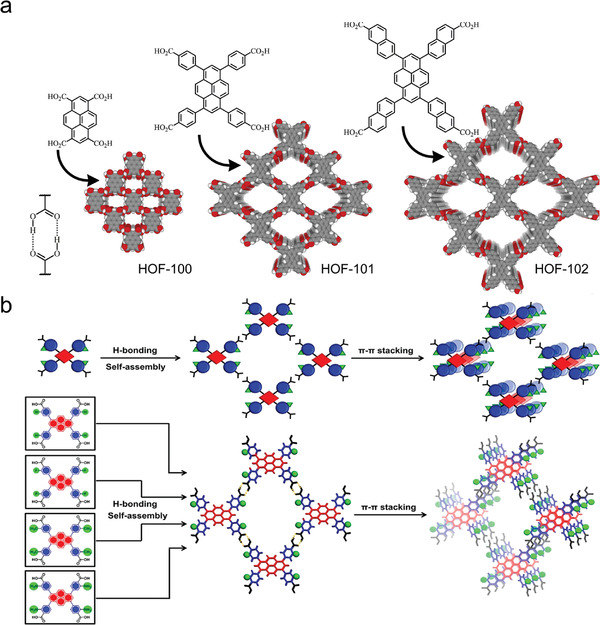
Adjustable pore size and surface ratio. a) Isostructural principle to tune the pore sizes of HOFs by increasing the linker sizes. Reproduced with permission.^[^ ^7b^ ^]^ Copyright 2022, Elsevier. b) Schematic illustration of the self‐assembly of HOF materials from various tectons. Reproduced with permission.^[^ ^36^ ^]^ Copyright 2021, Wiley‐VCH.

For HOF prototypes constructed with highly oriented hydrogen‐bonded structural units, adjusting the spacer lengths of the organic core will produce homo‐structured HOFs with the same network topology, thus enabling the pore size to be tuned. The use of ─COOH dimers allows for the rational design of HOFs, with their geometry playing a dominant role in shaping the framework topology (**Figure** **5**). Isostructural design gives HOFs tunable porosity parameters without changing their basic framework topology. In addition, the relatively loose stacking of HOF building blocks may lead to symmetry entanglements in the hydrogen‐bond network, which can be controlled by the substituents of the guest or host molecules.^[^ ^35^ ^]^ Chen et al. synthesized three isostructural 2D HOFs (Figure 5a)^[^ ^7b^ ^]^ with BET surface areas in the range of 900–2500 m^2^ g^−1^ and pore diameters in the range of 8–25 Å, respectively, by employing different coplanar tetracarboxylic acids. This shows how the pore diameter and BET surface area can be rationally adjusted by increasing the length of the linker. Recently, Li, Farha, and collaborators extended this platform by chemically engineering HOF‐101 (Figure 5b) by pre‐functionalizing the building blocks with CH_3_, F, and NH_2_ groups.^[^ ^36^ ^]^ This approach allows its structural properties and photosensitivity to be modulated. In addition, the unique properties of fluorine (high electronegativity and a small van der Waals radius of 1.47 Å) have attracted the use of fluorination to modify the chemical and physical properties of porous materials. For example, Miljanic and co‐workers reported a porous structure that could be self‐assembled with fluorinated aromatic pyrazole building blocks with BETs reaching 1159 m^2^g^−1^.^[^ ^22^ ^]^

**Figure 6:**
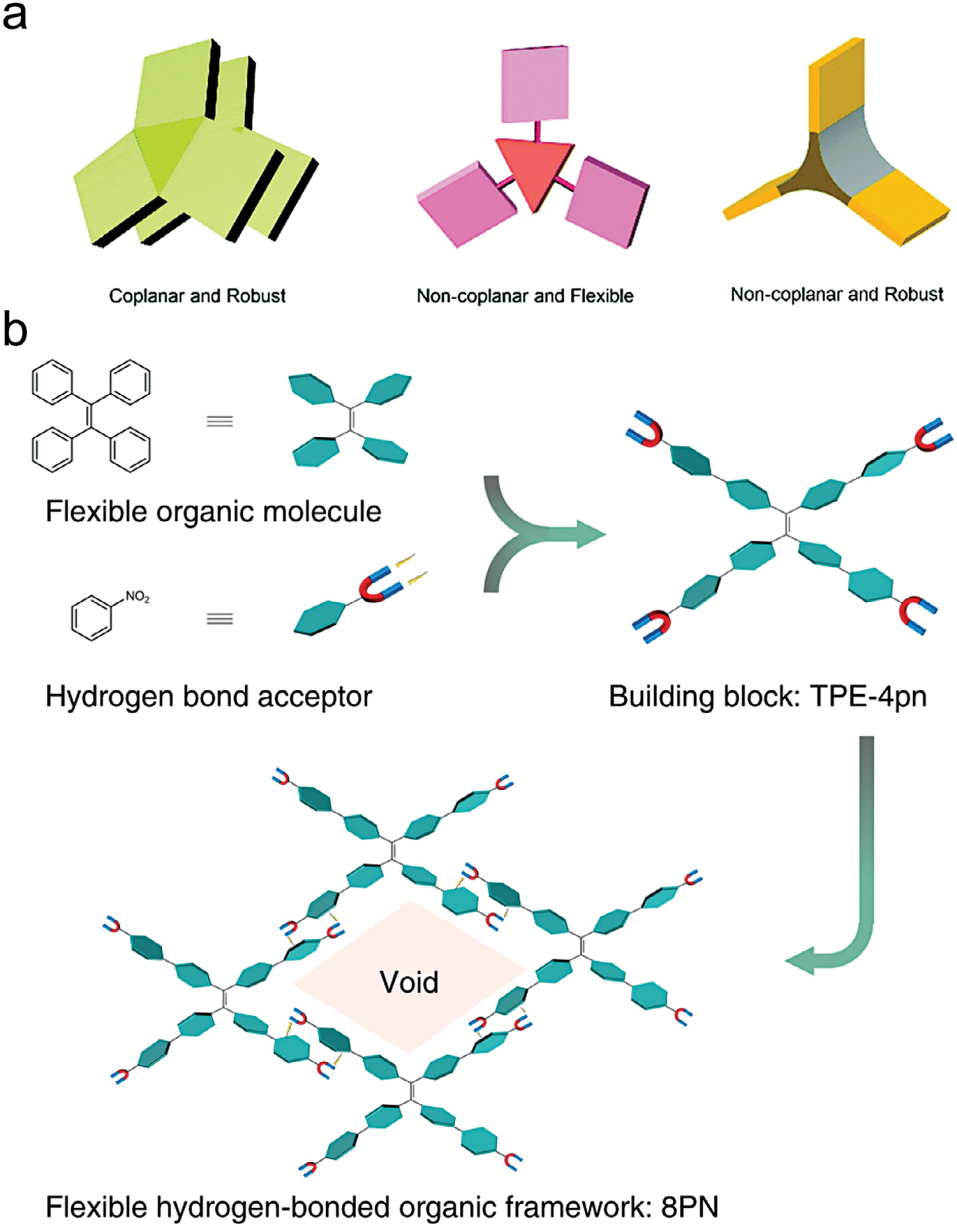
Flexible hydrogen‐bonded organic framework. a) Organic building blocks with distinct robustness and coplanarity were used to construct HOFs. Reproduced with permission.^[^ ^38^ ^]^ Copyright 2017, Royal Society of Chemistry. b) Design strategy for 8PN. Reproduced with permission.^[^ ^24a^ ^]^ Copyright 2019, Springer Nature.

Researchers also assembled three isostructural hydrogen‐bonded rhombic networks of benzene, tetrathiafulvalene, and pyrazinoquinoxaline tetracarboxylic acids, with showed pore sizes of 12.5 × 24.8, 14.9 × 27.0, and 12.5 Å × 28.7 Å, respectively.^[^ ^37^ ^]^ The isostructural triheptene imidazolidinone HOF had a larger pore size (19.9 Å) and larger BET surface area (3425 m^2^ g^−1^) than TTBI (14.5 Å and 2796 m^2^ g^−1^).^[^ ^20^ ^]^ Theoretically, the use of rigid, non‐coplanar organic units avoids potential π–π stacking, maximizes the exposure of molecular surfaces in the pores, and reduces the effect of molecular overlap on porosity. These properties make it the preferred strategy for constructing hydrogen‐bonded organoskeletal materials with high specific surface areas. For example, Li's group developed a tridecene‐based porous hydrogen‐bonded organoskeleton(FDM‐15) constructed from rigid, noncoplanar tridecene‐based organic building blocks.^[^ ^38^ ^]^ Its specific surface area reached 749 m^2^ g^−1^ with a channel diameter of 11.5 Å (**Figure** **6**a). Due to the absence of π–π interactions in the framework, FDM‐15 exhibited a high adsorption capacity and selectivity toward aromatic compounds. Chi et al. synthesized flexible HOF materials, named 8PN, using the organic small molecule 1,1,2,2‐tetrakis(4′‐nitro‐[1,1′‐biphenyl]−4‐yl) ethane (TPE‐4pn) as the fundamental unit.^[^ ^24a^ ^]^ This series of materials achieved large‐scale pore adjustment (total volume ratio variation in the range of 4.4–33.2%) by adjusting the solvent or reaction conditions. This approach was used to obtain nine single‐crystal structures with the same organic unit but different hydrogen bonding connection modes. 8PN underwent multi‐modal reversible structural transformations to obtain a series of high‐quality co‐crystals containing guests with different shapes, sizes, aggregation states, and even numbers of guests (Figure 6b).

##### Structural Isomerization and Polymorphism

**Figure 7:**
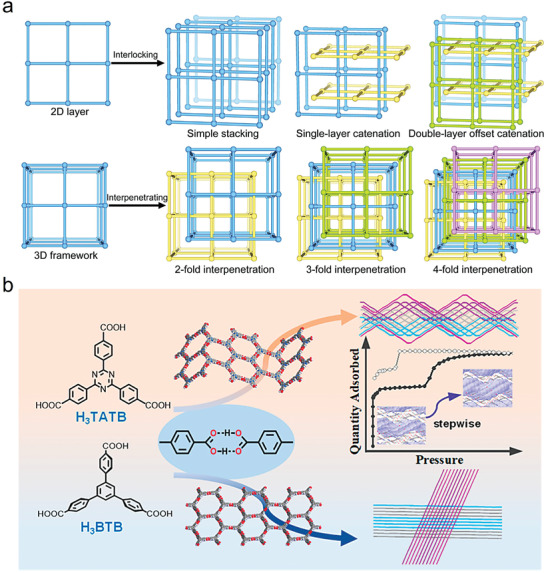
Structural isomerization and polymorphism. a) Interlock modes of 2D networks and interpenetrating single 3D networks into a multifold polycatenated structure (e.g., 2‐, 3‐, or 4‐fold interpenetration). Reproduced with permission.^[^ ^7b^ ^]^ Copyright 2022, Elsevier. b) Self‐assembly of rigid planar ligands produces planar hexagonal honeycomb patterns that form more complex structures under specific conditions. Reproduced with permission.^[^ ^24b^ ^]^ Copyright 2020, American Chemical Society.

Compared with MOFs and COFs, HOF structures have greater variability and polymorphism, which makes their design challenging. Small changes to the molecular structure can lead to dramatic changes in the assembled framework structures. For example, a planar rigid tetracarboxylic acid with a pyrene core can form a 2D HOF with an *sql* rhombic H‐bond network topology (PFC‐1), whereas a slightly twisted variant with a biphenyl core will form a 3D network with a dia topology in HOF‐TCBP (**Figure** **7**a).^[^ ^7b^ ^]^ Similarly, Li et al. found that the self‐assembly of rigid planar ligands produced planar hexagonal honeycomb patterns that could be expanded into undulating 2D layers to ultimately give rise to polycatenated HOFs with a range of complexities.^[^ ^24b^ ^]^ The absence of this undulation was observed in 2D layers constructed from very similar nonplanar ligands, indicating that a slight twist in the ligand leads to significant structural changes (Figure 7b). These changes confer upon the material a unique stepwise adsorption behavior at a certain pressure. This originates from movement between the intertwined hexagonal networks, as well as high chemical stability, phase transitions, and preferential adsorption of aromatic compounds in these HOFs. Their work contributed to the understanding of the self‐assembly behavior of HOFs and provided new insights into the rational design of HOF materials for practical applications.

The main design challenge for HOFs arises from the competition among weak hydrogen bonds that influence the spontaneous stacking of complex, self‐organized networks. Due to the low bond energies of hydrogen‐bonded pairs, the associated dissociation energies for reversible bonding are also low. This implies that crystallization between the body‐building modules also produces different polycrystalline forms with small differences in lattice energies,^[^ ^20b^ ^]^ including thermodynamic and kinetic products.

In addition to hydrogen bonding, other intermolecular interactions with similar bonding energies, such as π–π stacking and van der Waals interactions, also affect the crystallization of HOFs.^[^ ^7c^ ^]^ These can significantly stabilize a wide range of polycrystals resulting from various hydrogen bonding configurations under suitable crystallization conditions. This results in crystals that are sensitive to changes in solvent, guest molecules, concentration, and temperature. In principle, crystallization in highly concentrated solutions or short reaction times produces kinetic polycrystals, whose crystallization can be slowed by decreasing the solution concentration or the reaction temperature. This can overcome the barrier of activation energy conversion between different polycrystals. Polymorphs are found not only in identical constructed molecules with different linkages but also in molecules constructed with identical linkages and different degrees of framework entanglement, e.g., interpenetration or polyenylation.^[^ ^7b^ ^]^

Macrocyclic hydrogen‐bonded structures are common in HOFs constructed from large building blocks or from units with longer bonds. The high complexity of multiconnected networks creates entanglements that stabilize open frameworks with large spans and increase the associated molecular packing density while also reducing the pore size. The degree of chelation or interpenetration varies depending on the side group substitution or guest molecule.^[^ ^35^ ^]^ Seven H3BTB derivatives with methyl, methoxy, nitro, or amino substituents on the central benzene ring spontaneously assembled into a series of multi‐catenated hcb H‐bonded networks with four‐ to seven‐fold catenation. The non‐substituted H3BTB showed eight‐fold catenation with an H‐bonded hexagonal ring with a diameter >20 Å.^[^ ^35^ ^]^ Several different stacking/catenation modes were observed in this series of HOFs, including single‐layer offset catenation, double‐layer offset catenation, and rotated‐layer offset catenation. These highly complex entanglements were investigated by systematically evaluating the lattice energy necessary to lock in stable polymorphs.

The concept of energy‐structure‐function diagrams has been introduced, whereby computational predictions of crystal structures and a comparison of their relative lattice energies can help identify undiscovered structures.^[^ ^20b^ ^]^ For example, three new phases (T2‐β, T2‐γ, and T2‐δ) of triheptene imidazolidinone were identified and experimentally characterized. Following the crystallographic screening of this imidazolidinone molecule, activated T2‐γ with a BET surface area of 3425 m^2^ g^−1^ was obtained and used for methane storage. Crystallization solvents play a crucial role in stabilizing highly porous networks. Gao et al. systematically investigated the effect of solvents on this type of polymorphism, including hydrophilic and hydrophobic solvents.^[^ ^39^ ^]^ Crystallization solvents with high relative dielectric constants and good H‐bond acceptors of carboxylic acids (e.g., methanol and ethanol) stabilized the free ─COOH sites, thus facilitating the formation of HOF‐16. For alcohols with low relative dielectric constants and large molecular sizes such as *n*‐propanol and isopropanol, the HOF‐11 isomer was obtained. Three isomers were obtained for planar 4,4′,4″‐(1,3,5‐triazine‐2,4,6‐triyl)tribenzoic acid (H3TATB), i.e., PFC‐11, PFC‐12, and PFC‐13, which showed 24, 18, and 36‐fold catenation, respectively. This demonstrates the multi‐catenation of highly complex HOFs.^[^ ^24b^ ^]^ Interestingly, due to the large span of the macrocyclic H‐bond structure, the BET surface areas of interpenetrating PFC‐11 and PFC‐12 still reached 750 and 650 m^2^ g^−1^, respectively.

##### Structural Flexibility

The fixed orientation of hydrogen bonds is significantly weaker than that of covalent and coordination bonds. The bond angles of stronger hydrogen bonds can also vary (130–180°).^[^ ^7c^ ^]^ Hydrogen bonds have much lower bond energies, and their bond lengths (2.5–3.2 Å) are longer than those of most organic covalent bonds (1.2–1.5 Å).^[^ ^7b^ ^]^ The flexibility of hydrogen bonds permits relatively large deformations of local structures, enabling structural investigations that require the preservation of framework periodicity and single‐crystal properties. The rotation and vibration of hydrogen bonds can also bring about global framework deformations such as sliding, bending, expansion, and contraction. Understanding how to construct and utilize the flexibility of HOF structures is the key to applied studies of their properties.

**Figure 8:**
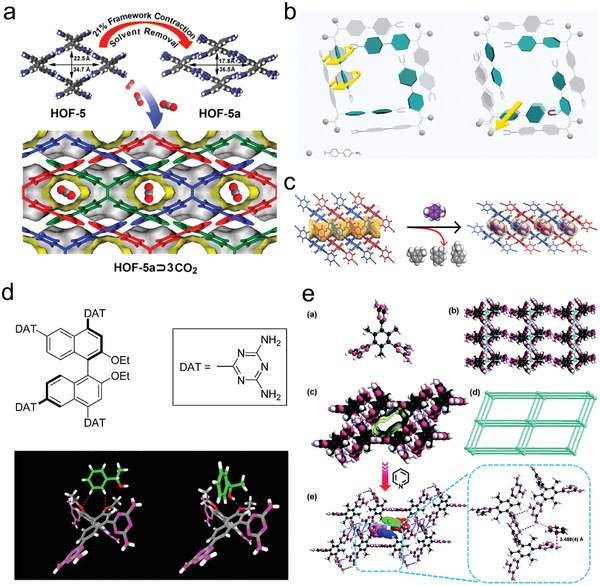
Structural flexibility of HOFs. a) Volume contraction demonstrating the flexibility of the HOF‐5 framework. Reproduced with permission.^[^ ^40^ ^]^ Copyright 2015, American Chemical Society. b) Illustration of the large‐scale regulation of the pore size by dihedral angles among different types of 8PN samples. Reproduced with permission.^[^ ^24a^ ^]^ Copyright 2019, Springer Nature. c) Schematic representation of the adaptive structural transformation from HOF‐29 to HOF‐29 ⊃ pX driven by the specific recognition of pX. Reproduced with permission.^[^ ^41^ ^]^ Copyright 2022, Wiley‐VCH. d) Organic building block to build HOF‐2 and the differential recognition of these two enantiomers by HOF‐2 (C, gray; H, white; N, pink; O, red). Reproduced with permission.^[^ ^43^ ^]^ Copyright 2014, American Chemical Society. e) The monomer configuration and crystal structure of HOF‐9 and its interaction with Py. Reproduced with permission.^[^ ^44^ ^]^ Copyright 2017, Royal Society of Chemistry.

The dynamic nature of hierarchically flexible hydrogen‐bond networks contributes to the development of complex functional materials.^[^ ^7b^ ^]^ HOF‐1 exhibited stepwise adsorption of carbon dioxide (CO_2_) and acetylene (C_2_H_2_) at different temperatures, which was attributed to its structural transformation upon guest loading.^[^ ^19^ ^]^ During the desolvation process of HOF‐5, it underwent a shrinkage rate of 21% by cell volume.^[^ ^40^ ^]^ The flexibility of HOFs was verified by stepwise dinitrogen (N_2_) adsorption isotherm with hysteresis, with an initial experimental pore volume of 0.44 cm^3^ g^−1^, which could be further expanded to 0.55 cm^3^ g^−1^, consistent with HOF‐5 without a guest (**Figure** **8**a). The flexibility of the framework is even more pronounced for HOFs without strong hydrogen bonding, such as 8PN assembled from nitroso‐tetraphenylethylene, which showed remarkable flexibility for accommodating solvent molecules.^[^ ^24a^ ^]^ The flexibility of 8PN was demonstrated by its different crystal structures upon solvent addition, whose cell volume varied in the range of 4.4–33.2% (Figure 8b). Through the responsive rotation of the benzene ring unit, 8PN crystals could accommodate different shapes, sizes, and numbers of guests. HOF‐29 provides another example of adaptive guest adsorption.^[^ ^41^ ^]^ Single‐crystal structural studies have shown that the single‐crystal‐to‐single‐crystal transition from synthesized HOF‐29 to fully *p*‐xylene‐containing HOF‐29 ⊃ pX occurred due to the encapsulation of the *p*‐xylene molecule, 2D layer sliding, and localized deformation of the ligand. This provides evidence that HOF‐29 has a flexible structure (Figure 8c).

In addition, due to the flexible structural regulation of HOFs, the introduction of a chiral center can realize the unique enantioselective separation of chiral objects, allowing HOFs to play an important role in molecular recognition. HOFs can provide asymmetric pores for enantioselective separation by introducing chiral centers, while additional hydrogen bond donors/acceptors provide binding sites for complementary analytes.^[^ ^42^ ^]^ For example, Li et al. used a chiral porous HOF for the enantioselective separation of small molecules for the first time.^[^ ^43^ ^]^ The constructed microporous HOF showed chiral pores that could enantioselectively separate small alcohols, with a higher selectivity for chiral secondary alcohols. The enantioselectivity of 1‐phenylethanol reached 92%. Analysis showed that the high enantioselectivity was induced by the chiral microenvironment within the framework and hydrogen bonding with the guest (1‐phenylethanol). The enantioselective separation of chiral substances can also be achieved by inserting structural sites with specific recognition functions into flexible structures (Figure 8d).^[^ ^43^ ^]^ For example, by modifying the free amino group on the inner surface of the pores of HOF‐9, the unbonded amino group formed strong N─H⋯N interactions with the Py molecule, thus realizing the effective chiral recognition and isolation of Py by HOF‐9.^[^ ^44^ ^]^ The flexibility of the HOF enabled it to exhibit adaptive framework changes during different guest encapsulation processes and realize the corresponding host‐guest interactions (Figure 8e). As mentioned above, HOFs show great potential for molecular recognition and transport.

HOFs can exhibit both robustness and flexibility by incorporating a robust backbone. The pores in flexible HOFs allow for the adsorption of small guest molecules and are then transformed into macropores that can adsorb larger guest molecules under the action of stronger triggers such as higher temperatures or pressures. For example, Chen's group prepared three‐fold‐interpenetrated HOF‐FJU‐1 which was both flexible and robust and showed a gating mechanism where the threshold pressure required for gas adsorption varies with temperature.^[^ ^45^ ^]^ As mentioned above, HOFs show great potential as guest hosts for molecular recognition and transport.

### 2.2 Strategies for Building Structurally Stable HOFs

**Figure 9:**
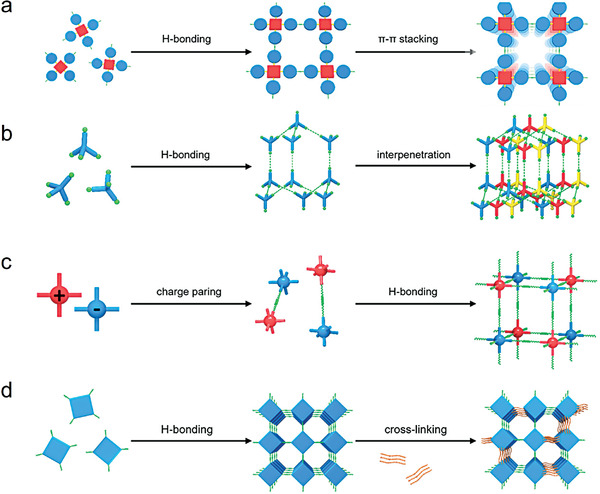
Commonly used strategies for constructing stable HOFs: a) π–π stacking, b) interpenetration, c) charge‐assisted hydrogen bonding, and d) cross–linking. Reproduced with permission.^[^ ^5a^ ^]^ Copyright 2022, American Chemical Society.

HOFs show environmental friendliness, easy regeneration and purification, metal‐free biocompatibility, adaptive structural transformation, solution processability, and recyclability.^[^ ^5a^ ^]^ However, they are less stable and show a limited possibility for synthesizing multifunctional materials.^[^ ^46^ ^]^ Solvents with low polarities and boiling points should be selected during synthesis to reduce the possibility of solvent coordination. Increasing the number of intermolecular hydrogen bonds,^[^ ^47^ ^]^ constructing multiple hydrogen bonds,^[^ ^43^, ^48^ ^]^ selecting rigid steric frameworks as hydrogen bonding building units,^[^ ^20^, ^49^ ^]^ and synergizing hydrogen bonding with other intermolecular interactions or even covalent bonds^[^ ^21b^ ^]^ can all be used to enhance the rigidity and stability of HOFs. This section summarises four common design strategies to enhance the stability of HOFs: π–π stacking, interpenetration, charge‐assisted hydrogen bonding, and chemical cross–linking (**Figure** **9**).^[^ ^5a^ ^]^ Synergistically enhancing an HOF's stability under various environmental conditions by using multiple strategies may expand their applications.^[^ ^27c^ ^]^ In most cases, more than one strategy was employed to synergistically enhance the stability of HOFs. For instance, π–π interactions, framework interpenetration, and electrostatic interactions were simultaneously introduced into BioHOF‐1, making it stable toward acidic pH, phosphate buffers, aqueous solutions, and even boiling water.^[^ ^27c^ ^]^ Zentner et al. reported that tcpb (1,3,5‐tris(4‐carboxyphenyl)benzene) assembled into an intricate eight‐fold‐polycatenated assembly of (6,3) hexagonal nets formed through a combination of hydrogen‐bonding and π‐stacking interactions. The resulting material showed excellent thermal and chemical stability.^[^ ^50^ ^]^ However, no study has systematically and accurately assessed the contribution of different strategies for improving the stability of HOF‐based materials toward various environmental stimuli. Future research should provide a deeper understanding of the characteristics of each strategy and their potential stacking or coupling to design more stable HOFs for practical applications.

#### 2.2.1 π–π Stacking

**Figure 10:**
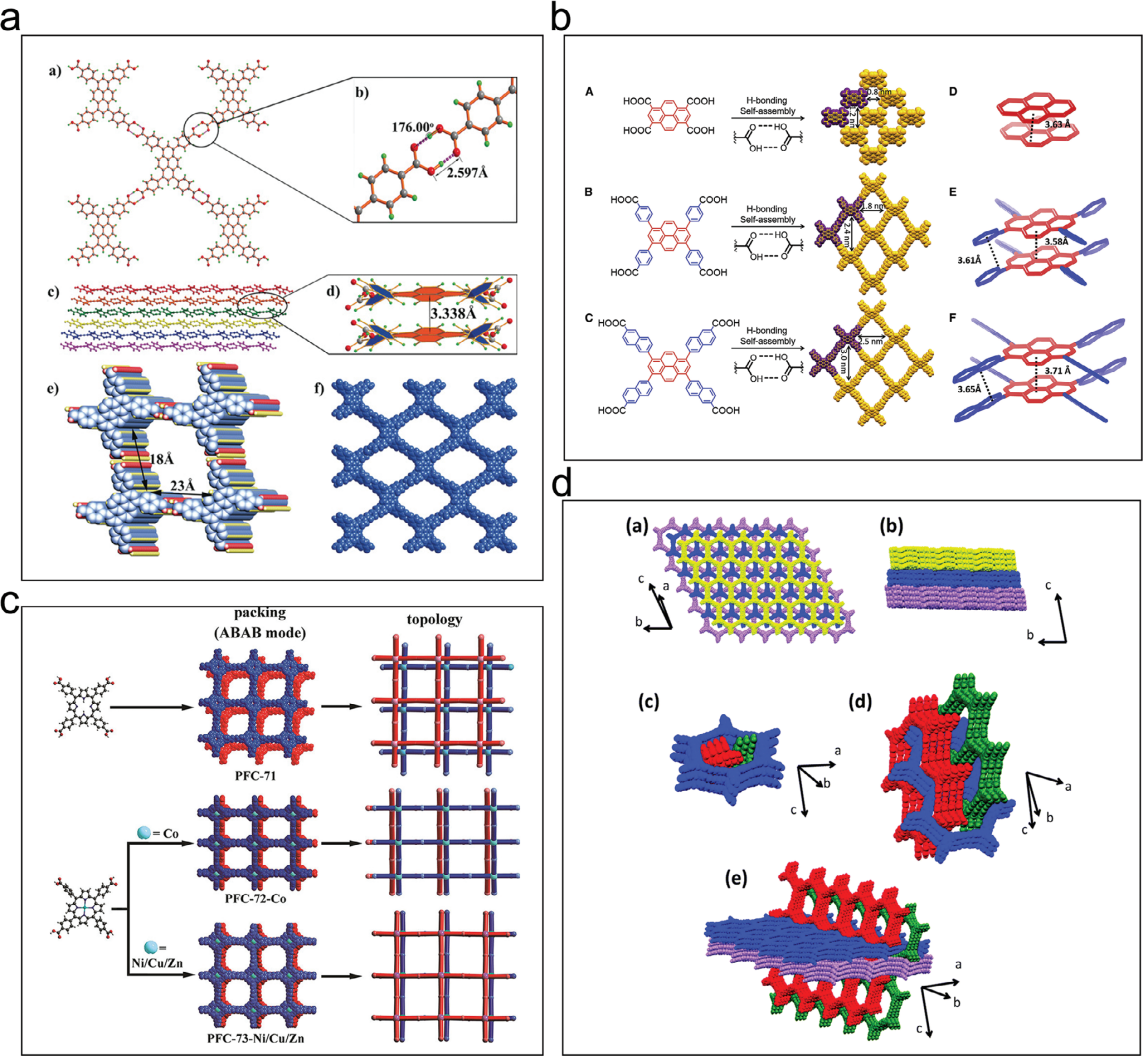
Examples of π–π stacking. a) Crystal structure of PFC‐1. Reproduced with permission.^[^ ^52^ ^]^ Copyright 2018, Wiley‐VCH. b) HOF‐100, PFC‐1/HOF‐101, and HOF‐14/HOF‐102 were prepared by C9–C11 building blocks, respectively. Reproduced with permission.^[^ ^49^ ^]^ Copyright 2020, Elsevier. c) Schematic representation of the construction of PFC‐71, PFC‐72‐Co, and PFC‐73‐Ni/Cu/Zn. Reproduced with permission.^[^ ^56^ ^]^ Copyright 2019, Wiley‐VCH. d) Crystal structure representations (P1 polymorph shown). Reproduced with permission.^[^ ^50^ ^]^ Copyright 2019, Royal Society of Chemistry.

π–π stacking refers to noncovalent interactions between aromatic rings, which provides a strategy for synergistically using hydrogen bonding and π–π stacking interactions to prepare stable HOFs.^[^ ^27c^ ^]^ Organic units are first connected by hydrogen bonds to form a 2D hydrogen‐bonded layer that is then populated by π–π stacking interactions to obtain 3D stabilized frameworks with 1D open channels along the filling direction. Planar and large π‐conjugated configurations with C2‐, C3‐, C4‐, and C6‐symmetries are well‐suited for constructing stable HOFs because of their easy accessibility to the 2D hydrogen‐bonding layer. The 2D square lattice (sql/net) is the most commonly used platform for constructing stable HOFs.^[^ ^51^ ^]^ Cao's group prepared a crystalline PFC‐1 based on 1,3,6,8‐tetrakis(*p*‐benzoic acid)pyrene (H4TBAPy), which consisted of a large π‐conjugated planar pyrene scaffold and four benzoic acid arms (**Figure** **10**a).^[^ ^52^ ^]^ In PFC‐1, each H4TBAPy was connected to four neighboring H4TBAPy molecules by a carboxyl dimer to form a 2D hydrogen‐bonded layer with an sql topology. Due to shape‐matching, the pyrene scaffold and four benzoic acid arms were perfectly stacked with the neighboring pyrene and benzoic acid molecules, respectively, in an AA pattern. Chen et al. replaced H4TBAPy (C10) with the H4PTTNA (C11) ligand with a larger conjugation system and prepared the isostructural HOF‐14,^[^ ^7a^ ^]^ which showed greater stability than PFC‐1. The stronger π–π interactions between the naphthalene ring and naphthyl ring replaced the benzene ring‐benzene ring π–π interactions of PFC‐1 so that its structure remained unchanged after being treated with concentrated hydrochloric acid, concentrated alkali, boiling water, and after heating at 300 °C for 2 h. Farha et al. used the topology principle to synthesize HOF‐100, HOF‐101, and HOF‐102 using H4TCPy (C9), H4TBAPy (C10), and H4PTTNA (C11), respectively. The stability of these structures followed the order HOF‐100 < HOF‐101 < HOF‐102 (Figure 10b).^[^ ^49^ ^]^

Molecules with planar structures and different π‐conjugation systems can create monolayer or multilayer structures through intermolecular hydrogen bonding interactions. These structures then form 3D frameworks that extend throughout the entire structure, facilitated by strong π–π stacking interactions.^[^ ^53^ ^]^ Zentner et al. constructed an octet of interpenetrating supramolecular structures assembled from Tcpb ((1,3,5‐triphenylene (4‐carboxybenzene)), which was formed through hydrogen bonding and π–π stacking interactions and showed permanent porosity (Figure 10d).^[^ ^50^ ^]^ Hisaki et al. utilized π–π stacking between a 1,2,4‐trichlorobenzene derivative containing carboxybenzene to construct acid‐responsive HOFs.^[^ ^54^ ^]^ π–π stacking interactions can also enhance the hydrothermal stability of HOFs. For example, HOF‐8 synthesized by Zhong et al. using N1,N3,N5‐tris(pyridin‐4‐yl)benzene‐1,3,5‐tricarboxamide (TPBTC) showed good hydrothermal stability.^[^ ^55^ ^]^ Yuan et al. used 3,3′,5,5″‐tetra‐(4‐carboxyphenyl)−1,1′‐biphenyl to synthesize an HOF with improved hydrothermal stability.^[^ ^21a^ ^]^ Yin et al. utilized π–π stacking to construct five metal‐porphyrin‐based HOFs, which showed the same topology but different filling patterns (Figure 10c).^[^ ^56^ ^]^ The metallization of the porphyrin centers gave PFC‐72 and PFC‐73 a high specific surface area and excellent thermal, chemical, and water stability. The materials maintained their structural integrity after immersion in boiling water, concentrated HCl, or even after heating to 270 °C.

Imparting cooperative π–π stacking interactions between 2D layers can reliably stabilize the overall framework structure. HOFs containing large, π‐conjugated aromatic molecules as building blocks exhibit superior thermal stabilities and increased chemical resistance to organic solvents and acidic or basic aqueous solutions due to the inertness of the tectons. Several groups have independently demonstrated that HOFs with shape‐fitted π–π stacking interactions show type III stability upon desolvation and are often even more stable due to a larger effective π–π stacking area. However, the poor directionality of π–π stacking interactions compared with other strong forces makes it difficult to control and predict their behavior when used to assemble highly stable HOFs.^[^ ^57^ ^]^

#### 2.2.2 Interpenetration

**Figure 11:**
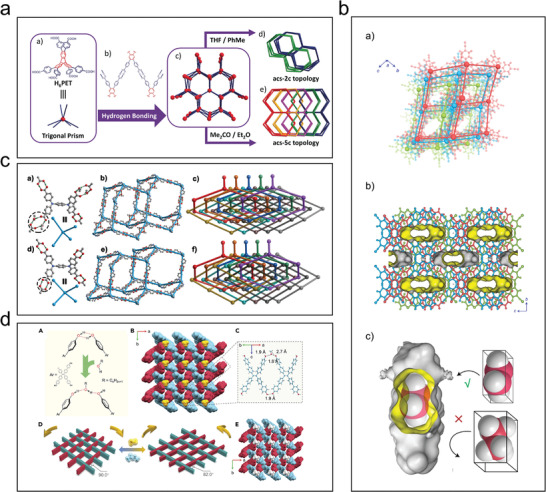
Examples of interpenetration. a) Assembly of a pair of interpenetrating isomers. Reproduced with permission.^[^ ^57^ ^]^ Copyright 2019, Wiley‐VCH. b) Crystal structure of HOF‐FJU‐1. Reproduced with permission.^[^ ^45^ ^]^ Copyright 2021, Springer Nature. c) Illustration of the reversible structural transformations between HOF‐30 and HOF‐30a. Reproduced with permission.^[^ ^58^ ^]^ Copyright 2019, Wiley‐VCH. d) 1D chemical structure and interlocking mode at the entanglement node in the SXRD structure of 2D‐90. Reproduced with permission.^[^ ^59^ ^]^ Copyright 2022, Elsevier.

Introducing appropriate interpenetrating structures is an effective method to improve the stability of HOFs. Although framework interpenetration reduces the pore size and number of pores in HOFs, the thermodynamic stability of such frameworks is higher.^[^ ^29b^ ^]^ Li et al. used H6PET as a building block to control the interpenetration by adjusting the synthesis conditions to obtain PETHOF‐1 with a double‐interpenetrating structure and PETHOF‐2 with quintuple interpenetrating structures (**Figure** **11**a).^[^ ^57^ ^]^ Multiple interpenetrating structures were constructed by the structural modification of the building blocks and by regulating the assembly conditions to improve the thermal and chemical stability of PETHOF‐2.

In 2017, Chen's group prepared a highly interspersed and stable microporous HOF‐11 by slowly diffusing *n*‐hexane into a tetrahydrofuran solution containing tris(4‐carboxyphenyl)amine.^[^ ^28^ ^]^ Single‐crystal X‐ray diffraction analyses of HOF‐11 showed that it had a stable 3D ammonia‐bonded mesh structure with an 11‐fold interspersed topological type was (10,3)‐b with a pore size of 6.2 Å × 6.8 Å. Hu et al. utilized interpenetration to construct a diamond‐type topological network a permanent 3D porous structure and a five‐fold interpenetrated structure that could selectively adsorb and separate light hydrocarbons. It also exhibited high thermal and chemical stability in an aqueous solution.^[^ ^21a^ ^]^ Yang et al. constructed a tetracyanourazole building unit based on intermolecular CN⋯H─C hydrogen bonds through an interpenetration strategy to form a three‐layer interpenetrating framework of the microporous HOF‐FJU‐1, which was extremely stable in strongly acidic, alkaline, and highly polar solvents (Figure 11b).^[^ ^45^ ^]^

Chen et al. reported the assembly of *N,N,N*′*,N*′‐tetrakis(4‐carboxyphenyl)−1,4‐phenylenediamine in methanol, ethanol, and *n*‐propanol, respectively, to obtain a series of structures such as HOF‐30 with permanent microporosity.^[^ ^58^ ^]^ All three HOFs showed a 3D ten‐fold interpenetrating 2D network containing two hydrogen bonding modes: a carboxyl⋯carboxyl dimer and a two‐solvent bridged carboxyl dimer (Figure 11c). Recently, Chi's group developed a dynamically 2Dly braided, 1D chain interlocked structure.^[^ ^59^ ^]^ This structure exhibits mechanical stability while undergoing dynamic, reversible structural transformations in the presence of solvent vapors, such as methanol or ethyl acetate (Figure 11d). This finding provided a new idea for the use of HOFs in molecularly braided single‐crystal structures for designing structurally stable and controllable interspersed interlocking structures.

In short, structural intercalation and interlocking often improve the stability of HOFs, but this approach reduces their porosity and makes the structure of HOFs more difficult to predict and control. Although framework interpenetration reduces the pore size and pore voids of HOFs, it improves the framework's stability because the multiple‐interpenetrating framework is more thermodynamically favorable than its non‐interpenetrating counterpart. Therefore, framework interpenetration is a common strategy to strengthen HOFs.

#### 2.2.3 Charge‐Assisted Hydrogen Bonding

**Figure 12:**
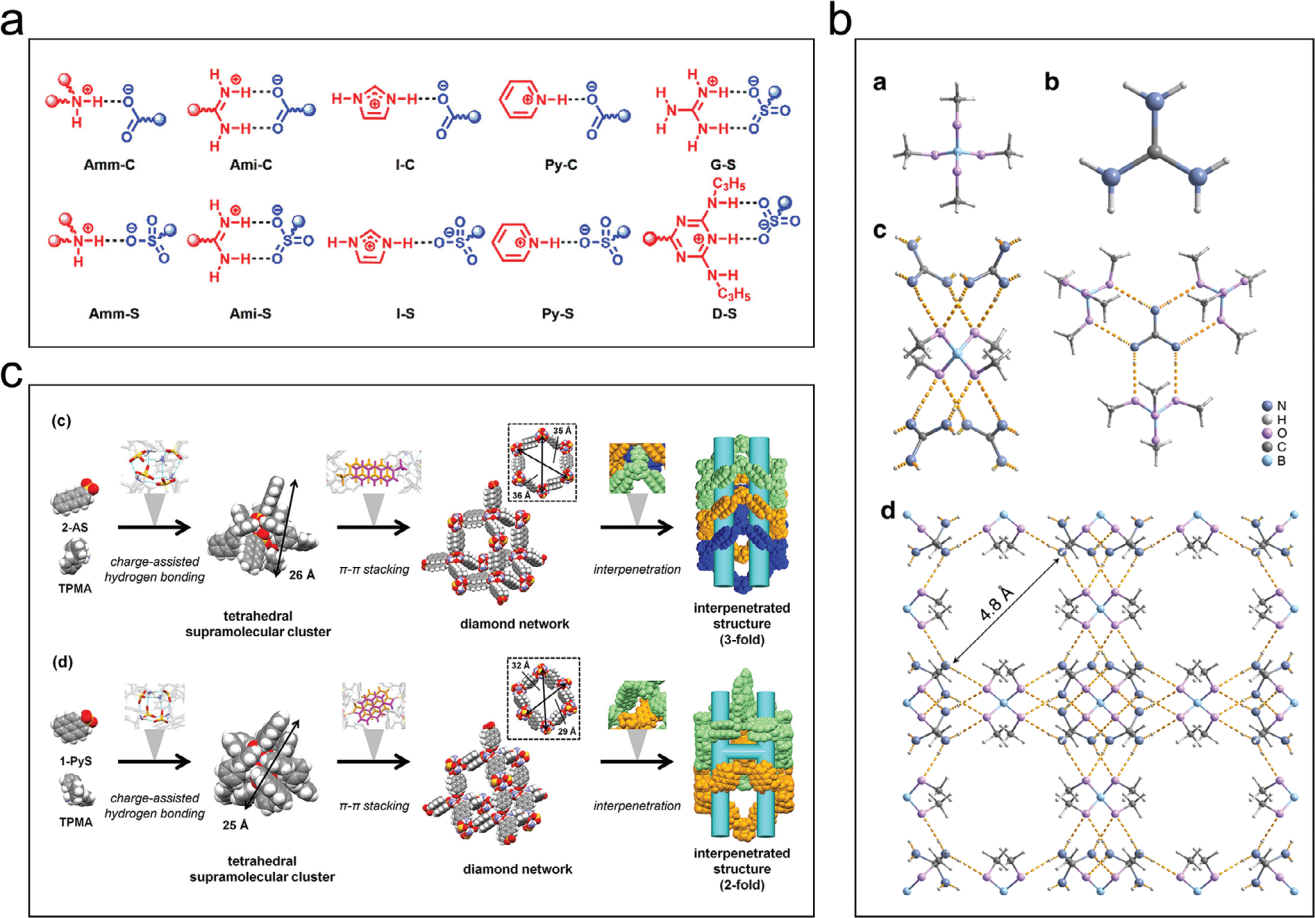
Examples of charge‐assisted hydrogen bonding. a) Various types of charge‐assisted hydrogen‐bonding motifs. Reproduced with permission.^[^ ^5a^ ^]^ Copyright 2022, American Chemical Society. b) Crystal structure of Gd‐B. Reproduced with permission.^[^ ^72^ ^]^ Copyright 2020, Springer Nature. c) Crystal structures of *d*‐POSs. Reproduced with permission.^[^ ^73^ ^]^ Copyright 2012, American Chemical Society.

Introducing electrostatic interactions is another viable strategy for improving the stability of HOFs by forming charge‐assisted hydrogen bonds through strongly acidic or basic components to enhance the bonding strength.^[^ ^29b^ ^]^ Many supramolecular synthetics containing charge‐assisted hydrogen bonding interactions are listed to synthesize robust and multifunctional HOFs (**Figure** **12**a),^[^ ^5a^ ^]^ including ammonium‐ carboxylate,^[^ ^60^ ^]^ amidinium‐carboxylate,^[^ ^47^, ^61^ ^]^ imidazolium‐carboxylate,^[^ ^62^ ^]^ pyridinium‐carboxylate,^[^ ^63^ ^]^ guanidium‐sulfonate,^[^ ^64^ ^]^ ammonium‐sulfonate,^[^ ^27b^ ^]^ amidinium‐sulfonate,^[^ ^65^ ^]^ imidazolium‐sulfonate,^[^ ^66^ ^]^ pyridinium‐sulfonate,^[^ ^67^ ^]^ or diaminotriazinium‐sulfonate.^[^ ^68^ ^]^

In 1994, Ward's group pioneered the assembly of supramolecules using rationally designed charge‐assisted hydrogen‐bonding interactions.^[^ ^69^ ^]^ Subsequently, it was found that novel 2D hydrogen‐bonded sheets^[^ ^70^ ^]^ and 3D hydrogen‐bonded cages^[^ ^71^ ^]^ could be constructed by utilizing interactions between guanidinium ions and sulfonate anions. The resulting HOFs exhibited permanent porosity during gas adsorption. Karmakar et al. used a hybrid ligand strategy of charge‐assisted hydrogen bonding to construct two types of porous 2D HOFs with aromatic sulfonate and guanidinium‐based cations as the building blocks.^[^ ^27a^ ^]^ Both exhibited excellent proton conductivity properties and stability in humid or high‐temperature environments. Recently, Wang et al. constructed a stable HOF (Figure 12b) constructed from guanidinium cations and borate anions that reversibly adsorbed and desorbed methanol at room temperature.^[^ ^72^ ^]^ The results suggested that charge‐assisted hydrogen bonding formed porous HOFs with potential applications for capturing and releasing target analytes.

Yamamoto et al. constructed porous structures with a rhombic network (d‐POSs (Amm‐S)) generated by combining triphenylmethylamine (TPMA) and sulfonic acid using a charge‐assisted hydrogen bonding strategy.^[^ ^73^ ^]^ In d‐POSs, TPMA and sulfonic acid served as the main building blocks and assembled into stable tetrahedral supramolecular clusters through charge‐assisted hydrogen bonding (Figure 12c). The organic salts of TPMA and 2‐AS produced polymorphic structures in response to varying the host‐guest ratio and the type of guest, demonstrating the stability and flexibility of d‐POS. Liu et al. used charge‐assisted hydrogen bonding to construct HOFCo*x*Fe1‐*x* with Co and Fe nanoparticles in its pores and verified the potential of charge‐assisted HOFs for electrocatalytic water splitting. They also remained stable and achieved excellent OER and HER performance in 1 mol^−1^L^−1^ potassium hydroxide solution.^[^ ^74^ ^]^

Employing a mixed‐ligand strategy is a novel but challenging method to increase the diversity of HOF‐based materials. The use of charge‐assisted hydrogen‐bonding interactions that can form through strong acidic or basic components, can also afford stable HOF structures with unique framework interactions. Such HOFs typically show improved thermal and chemical stabilities, but the combination of multiple hydrogen bonding modes increases the difficulty of predicting the structure of the final synthesized HOF. This also increases the synthetic difficulty because more variables affect the final structure.

#### 2.2.4 Chemical Cross–linking

**Figure 13:**
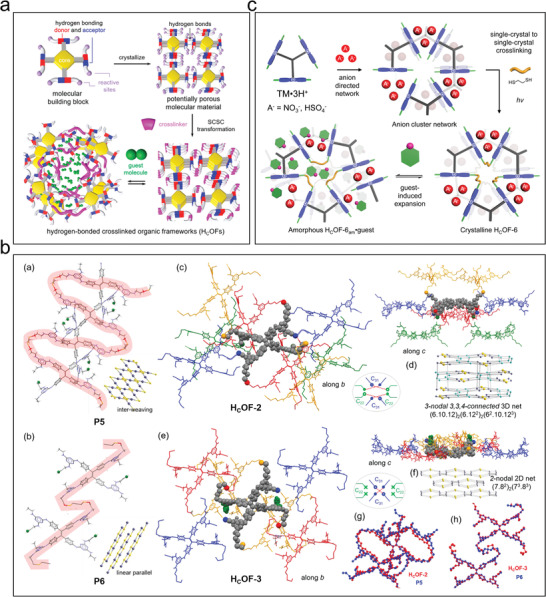
Examples of chemical cross–linking. a) Illustration of the design of HCOFs through SCSC transformation. Reproduced with permission.^[^ ^75^ ^]^ Copyright 2017, American Chemical Society. b) Schematic representation of P5 and P6 forming an HCOF by cross–linking. Reproduced with permission.^[^ ^76^ ^]^ Copyright 2019, American Chemical Society. c) Illustration of the anion‐cluster‐directed formation of a hexagonal hydrogen‐bonded network and the synthesis of HCOF‐6 through SCSC cross–linking. Reproduced with permission.^[^ ^68^ ^]^ Copyright 2021, Elsevier.

Connecting structural units using cross–linked structures can enhance the stability of HOFs. Ke et al. developed a series of hydrogen‐bonded cross–linked organic frameworks (HcOFs) by pre‐constructing organic structural units of HOFs via covalent photoinduced cross–linking.^[^ ^75^ ^]^ A crystalline HOF was first constructed using tetrastyrene‐functionalized building units, and then the photochemical reaction between alkynes and thiols was used to cross–link HOFs in the single‐crystalline state. Ultimately, the porous polymer HcOF‐1 was prepared (**Figure** **13**a), which showed a robust crystalline structure with good chemical stability. It remained structurally undamaged in acidic and alkaline aqueous solutions with a pH range of 0–14. HcOFs showed guest‐induced elastic expansion and contraction and were reversibly restored to their crystalline morphology and initial voids during adsorption and desorption.

Based on this work, Jiang et al. converted hydrogen‐bonded organic framework single crystals to cross–linked single crystals by a photocross–linking reaction, where disulfide cross–linking reagents with different alkane chain lengths were used to modulate the topology of the final H_C_OF (Figure 13b).^[^ ^76^ ^]^ Flexible disulfide linkages increased the volume of voids that could be activated by guest molecules, and the H_C_OF porosity increased to a maximum of 473% of its initial volume after cross–linking. Incorporating sulfhydryl groups also enabled photocross–linking, and the cross–linked structure facilitated dynamic adsorption (e.g., a high adsorption capacity and high selectivity), while the flexible linkages stabilized the structure. Recently, Samanta et al. synthesized a novel hydrogen‐bonded cross–linked H_C_OF‐6 (Figure 13c) using an ionic cross–linking reagent.^[^ ^68^ ^]^ Due to homo‐charge repulsion in the cross–linking reagent, the backbone extended up to two times its original length. Simultaneously, the alkane chains in the cross–linking linker restricted the infinite expansion of the porous framework, preserving its structural integrity. This expandable structure design facilitated guest adsorption, while the cross–linking chain strategy restricted the over‐expansion of the crystals, thus keeping the network structure intact and stable. The joints were composed of ions that repelled each other but were coaxed into place via interactions with other molecules in the scaffold. In the presence of a suitable chemical, however, these interactions were readily disrupted, and the ions repelled each other. These interactions forced the crystal to expand, while the cross–linkers limited the expansion and kept the porous network intact. In general, the applications of cross–linked structures in HOFs have mainly concentrated on dynamic sorption behavior, such as targeting a high adsorption capacity and high selectivity. These elastic junctions are very helpful for ensuring a HOF's stability during such processes.

## 3 Synthesis Methods of HOFs

### 3.1 Common Synthesis Methods of HOFs

**Figure 14:**
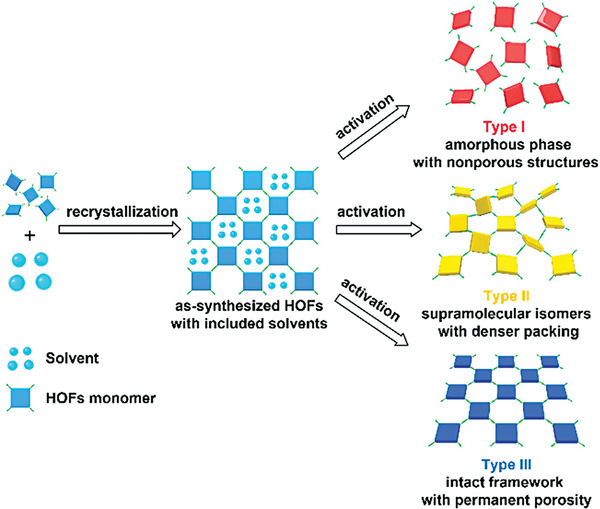
Schematic depiction of the recrystallization of HOF monomers from a solvent to generate solvent‐included HOFs, as well as three possible guest removal pathways: non‐porous amorphous phase (type I), supramolecular isomers with denser packing (type II), or intact frameworks with permanent porosity (type III). Reproduced with permission.^[^ ^5a^ ^]^ Copyright, 2020 American Chemical Society.

HOFs are often solvated crystals, and crystalline synthesis is susceptible to multiple factors.^[^ ^7c^ ^]^ The activation of HOFs to remove guest molecules from their pores usually forms three different material types (**Figure** **14**),^[^ ^5a^ ^]^ namely poreless amorphous phases, adaptive lattice rearrangement supramolecular polycrystals, and pristine arrangements that retain the molecular configuration. The activation of removing guest solvent molecules often leads to HOF collapse, making it challenging to prepare pore‐stabilized HOFs.^[^ ^77^ ^]^

It is crucial to develop and improve the synthetic strategies for HOFs. The main synthetic methods for HOFs include solvent volatilization, diffusion, solvothermal synthesis, recrystallization, and stirring methods.^[^ ^29^, ^30^, ^78^ ^]^ Recently, novel methods such as electrophoretic methods, template methods, and indirect methods with post‐synthesis modifications have also been reported.^[^ ^29^, ^79^ ^]^ Each method has its own advantages and shortcomings, and choosing different synthesis methods will affect the final material's structure. A comprehensive introduction to the basic principles and typical cases of synthesis methods is helpful for reference and selecting the appropriate method.

#### 3.1.1 Solvent Evaporation Method

Solvent evaporation uses a supersaturated solution, in which reactants are first dissolved in the solvent. Then, the solution is obtained at a specific temperature, and the solvent is slowly evaporated to obtain the crystal product. It usually requires the use of a lower‐boiling solvent which may result in a longer reaction time. Moreover, it is difficult to control the volatilization rate precisely, which may result in uneven crystal size or the formation of amorphous products. Yuan et al. prepared colorless crystals of HOF‐TCPB by dissolving the TCBP ligand in DMF, followed by allowing acetone to slowly evaporate at room temperature for 3 days. Then, it was placed at 50 °C for 2 days and finally degassed for 10 hours under a dynamic vacuum condition at 70 °C.^[^ ^21a^ ^]^ X‐ray crystallographic analysis showed that HOF‐TCBP crystallized in an orthogonal *Fddd* space group with a unique 3D framework and a 5‐layer interpenetrating hydrogen bond structure. In the crystal structure, the H4TCBP molecule had a distorted tetrahedral configuration, which was attributed to the rotation of the C─C bond between the two phenyl rings of the biphenyl molecule. Gao et al. placed tris(4‐carboxyphenyl)amine in anhydrous methanol and heated the mixture in an oven at 80 °C for 5 min to obtain a clarified solution. Then, the solution was cooled to room temperature to obtain yellowish crystals of HOFs (named HOF‐16) with single‐crystal morphologies.^[^ ^39^ ^]^ Single‐crystal X‐ray diffraction analysis showed that HOF‐16 crystallized in the trigonal Rc space group. The asymmetric unit of HOF‐16 consisted of one‐half of a TCA molecule and one methanol molecule. The two carboxylic acid groups of TCA formed hydrogen bonds with two adjacent TCA molecules via a carboxylic acid dimer. Luo et al. dissolved tetrakis(carboxyphenyl)‐porphyrin in *N*, *N*‐dimethylformamide, and the filtrate obtained by cooling from a high temperature was slowly evaporated under ambient conditions, and cubic crystals of purple HOFs were obtained after two weeks.^[^ ^80^ ^]^ In addition, because temperature affects crystal formation and properties, a series of 0D TCPP HOFs (the lattice number of DMF molecules ranged from 2 to 6) was prepared by precisely increasing the solution temperature from 120 to 140 °C. This improved the photocatalytic activity of TCPP.

#### 3.1.2 Vapor Phase/Liquid Phase Diffusion

The diffusion synthesis methods for HOF mainly include gas‐phase diffusion and liquid‐phase diffusion, typically requiring long reaction times (days, weeks, or even longer). The crystals obtained through these methods have larger particles and better crystallinity. The vapor phase diffusion method involves dissolving an organic ligand in a benign solvent with a high boiling point. Subsequently, another volatile and undesirable solvent (such as methanol, acetone, chloroform, ethanol, or ethyl acetate) is diffused into the initial solution through volatilization in a closed container. This decreases the solubility of the solute, thus forcing a crystalline product to precipitate. The most commonly used method is the vial‐over‐vial method, which utilizes the solubility of a substance to cultivate the crystalline products of HOFs.^[^ ^29a^ ^]^ The specific procedure involves choosing suitable organic ligands that will be a liquid at room temperature or close to room temperature when first dissolved and then filtered to remove impurities. The filtrate is then transferred to a vial, placed in a low‐boiling volatile poor solvent vapor atmosphere, and then sealed at atmospheric pressure. The poor solvent is slowly evaporated into the dissolved organic ligands in the process of good solvent. Due to molecular diffusion, organic ligands slowly precipitate in the form of crystals. The purported advantage of the diffusion method is that high‐quality HOF single crystals can be obtained through the slow diffusion reaction of two or more solutions at their interface. However, the diffusion process is slow, requires a long time to produce a sufficiently large crystal, and is sensitive to diffusion conditions (such as temperature and concentration gradient). For example, Zhang et al. dissolved 2,4,6‐trimethylbenzene‐1,3,5‐isophthalate triethylene (TMBTI) in a beaker containing tetrahydrofuran (THF), which was then placed in a sealed beaker containing ether.^[^ ^81^ ^]^ Solvent vapors were allowed to slowly diffuse for two weeks at room temperature to grow HOF crystals suitable for single‐crystal XRD analysis, which showed that JLU‐SOF3 crystallized in a hexagonal space group with a hydrogen‐bonded 3D network. Yang et al. dissolved 1,3,6,8‐tetrakis(*p*‐benzoic acid)pyrene (H4TBAPy) by heating it in a small beaker containing *N*, *N*‐dimethylacetamide (DMA), and then the small beaker was placed into a larger one containing chloroform.^[^ ^82^ ^]^ The large beaker was sealed with a sealing film. Vapor was allowed to slowly diffuse for two weeks at room temperature, and yellow lumpy HOF crystals were collected. The PXRD patterns of the prepared solid products were highly consistent with previous studies,^[^ ^52^ ^]^ further indicating that the prepared PFC‐1 had a high crystallinity and high phase purity. The synthesis of HOF materials by gas‐phase diffusion is simple, but it requires a long crystal growth period.

The liquid phase diffusion method requires that reactants be completely soluble at room temperature. One reaction solution is added to the device and then another reaction solution is placed over it, sealed, and left to stand at room temperature. The two reactants react at their interface by solvent diffusion, and a buffered solvent is generally added to control the rate of diffusion to obtain a better crystal quality and shape. Sarkar et al. used this method to synthesize a copper‐based complex [Cu_2_(PDA)_2_(Ald)_2_(H_2_O)_2_] by solvent diffusion using 2,4‐pyridinedicarboxylic acid (PDA) and 2,2′‐dithiodipyridine (Ald) as ligands.^[^ ^83^ ^]^ In the obtained structure, Ald groups were first interconnected to form a dimer, which then formed a hydrogen‐bonded lamellar structure via interlayer hydrogen bonding. Chen et al. used a slow diffusion method to synthesize HOF‐5^[^ ^40^ ^]^ and HOF‐10^[^ ^84^ ^]^ with different structures by slowly diffusing THF into 4 mL of DMF/DMSO (*v*: *v* = 1: 1) and 6 mL of DMF/DMSO (*v*: *v* = 2: 1) solutions containing the DAT‐TPE ligand, respectively. The hydrogen bond donor and acceptor should have limited solubility in each other's solvents, and the procedure should be slow to obtain a better crystal quality and morphology. The solution composition and DAT‐TPE concentration play important roles when using self‐assembly to generate different porous HOFs.

#### 3.1.3 Solvothermal Synthesis Method

Solvothermal synthesis uses an organic solvent as the reaction medium in a closed system when the basic organic compound ligand is insoluble at room temperature. The reaction temperature and concentration of organic ligands in the reaction medium greatly influence the synthesis. The potential advantage of solvothermal synthesis is that the reaction can be performed under a high temperature and pressure, which can accelerate the growth rate of crystals and obtain larger crystals. However, the reaction conditions are harsh, requiring special equipment and operating skills, and the high temperature and high pressure may lead to side reactions. In addition, the selection of suitable solvents helps obtain well‐orientated, perfect crystals. During a solvothermal reaction, solvents with low polarity and low boiling point should be chosen to reduce the possibility of solvent coordination. Commonly used solvents are DMSO, NMP, CH_3_CN, DMF, DMA, and anhydrous ethanol, which are compatible with the reaction mechanism of solvothermal synthesis. Moreover, using the same ligand in different solvents will affect the reaction thermodynamics and final products, which will change the hydrogen bonding mode of action, framework structure, and characteristics during the synthesis of HOFs.^[^ ^59^, ^85^ ^]^

Commonly used solvothermal methods include the vacuum tube sealing method and reactor method.^[^ ^29a^ ^]^ Due to the simplicity of the solvothermal method, faster crystal growth, higher crystal quality, and the ability to synthesize high‐weft structures, it is the most commonly used method to synthesize HOFs. The vacuum tube sealing method involves placing organic ligands and solvents into a reaction vessel, accelerating the dissolution by using ultrasonic equipment, and then sending the mixture into the oven to prepare porous HOF crystals under a high‐temperature and high‐pressure environment. The reactor method involves placing an organic ligand in a special reactor or pressure‐resistant glass vial and promoting the slow dissolution of the organic ligand and self‐assembly to complete the rearrangement by a high temperature to directly form a thermodynamically stable crystalline state. It may also involve exploiting the face that the organic ligand solubility decreases as it cools, thus causing the slow precipitation of crystals. Vaidhyanathan et al. prepared needle‐like colorless crystals of IISERP‐HOF1 by the solvothermal synthesis of a tricarboxytriphenylamine ligand dissolved in acetic acid and then transferred it to a PTFE‐lined reactor at 150 °C for 3 days.^[^ ^86^ ^]^ Single‐crystal XRD revealed that the IISERP‐HOF1 structure contained strong COOH⋯HOOC─ hydrogen bonds and *sp* ^3^‐nitrogen centers, giving it a non‐planar molecular shape. The presence of two types of hydrogen bonds between the carboxylic acid molecules allowed them to form a dimeric columnar structure, aligned along specific axes and stacked along the *bc* plane. This formed 1D channels through inter‐column hydrogen bonding, consisting mainly of carboxylic acid groups and phenyl rings with a triangular topology. TGA analysis showed that its thermal stability exceeded 280 °C. The loss of water and acetic acid and the lack of framework collapse at 90–150 °C demonstrated that acetic acid served as a template. The material was grown with acetic acid as the solvent and was thus stable to acid. The crystallinity was enhanced after immersion in 3N HCl, probably due to the recrystallization of the framework and a reduction in the number of molecular defects. In conclusion, IISERP‐HOF1 showed high thermal stability, acid stability, and well‐defined structure. Wang et al. dissolved 4,4′‐diamino‐2,2′‐stilbene disulfonic acid in DMF and then added aqueous tetramethylammonium hydroxide. This was followed by sonicating the mixture before heating it at 90 °C for 2 days to obtain yellow bulk HOFs (UPC‐H7).^[^ ^87^ ^]^ UPC‐H7 was a yellow crystal assembled from H2DAS and TMAOH. Single‐crystal X‐ray diffraction analysis showed that the asymmetric unit contained one DAS^2−^ anion and two TMA^+^ cations. The DAS^2−^ anion formed 2D anionic layers through short N─H⋯O hydrogen bonds, which were subsequently connected to each other to form a 3D structure by long N─H⋯O hydrogen bonds. The TMA^+^ cations were located between the 2D layers, which enhanced the stability of the structure via electrostatic interactions. PXRD and elemental analysis confirmed the phase purity of UPC‐H7. After being heated to 80 °C, the PXRD peaks of UPC‐H7 were slightly shifted, indicating the formation of a new phase, UPC‐H8. Single‐crystal XRD analysis showed that UPC‐H8 had the same space group and structural features as UPC‐H7 but with slight contraction or elongation along different axes.

#### 3.1.4 Simulated Annealing

The recrystallization method utilizes the different solubility of various components in the solvent to separate crystals. A simplified procedure involves dissolving an organic ligand in a solvent under heating and then filtering to remove impurities, cooling to room temperature, and then re‐filtering the unstable amorphous crystalline product that rapidly precipitates. The clarified solution is left to stand at room temperature to obtain a stable, more crystalline, and purer product. The potential advantages of recrystallization are as follows: a) the purity of HOF crystals can be increased through the process of dissolution and recrystallization, and b) the morphology and size can be improved. However, it is crucial to find the right solvent and crystallization conditions, otherwise the ideal crystal may not be obtained. Furthermore, part of the product may be lost during recrystallization, which can result in an impact on the synthetic yield. Chen et al. assembled tetracyanoorganic building blocks into HOF‐40 via hydrogen bonding with narrow 1D pores (4.15 Å × 3.85 Å).^[^ ^88^ ^]^ This was prepared by simple recrystallization from organic solvents, was easily recycled, and showed excellent chemical stability in 12 m HCl and 20 m NaOH. The organic building block 1,2,4,5‐tetrakis(4‐cyanophenyl)benzene (TCPB) was obtained from tetrabromobenzene and cyanophenylboronic acid by a coupling reaction. Single‐crystal X‐ray diffraction (SCXRD) analysis showed that HOF‐40 crystallized in a monoclinic *I2/c* space group, with half of the asymmetric unit being organic linkers and half solvent molecules. Each organic building block of HOF‐40 was connected to six adjacent organic linkers by two types of 12 intermolecular C─H···N≡C hydrogen bonds (the H···N distance ranged from 2.477 to 2.757 Å). The linkers formed a single *pcu* network and then formed a double‐interpenetrating 3D framework due to large gaps in the single network. HOFs were regenerated through a simple recrystallization process, and such materials showed good solvent repair/self‐healing ability, which may help reduce the costs of HOFs during practical applications. For example, Yuan's research group prepared a remarkably stable HOF‐based material (HOF‐TCBP) with a large specific surface area. After gas adsorption, HOF‐TCBP was dissolved in a small amount of DMF and was regenerated by spinning evaporation.^[^ ^21a^ ^]^ The regenerated HOF‐TCBP had almost the same specific surface area and properties as the original sample. Similarly, a UPC‐HOF‐6 film could also undergo structural self‐repair when treated with a small amount of solvent.^[^ ^21b^ ^]^

#### 3.1.5 Stirring Method

The stirring method can be used to rapidly synthesize HOFs, in which one reaction solution is added to a reaction apparatus, and then another reactant is added under rapid mixing and stirring, and then the crystal product is formed. The advantage of stirring method is that the reactants can be fully mixed, therefore it can accelerate the reaction rate and produce a more uniform product. However, there are some potential issues that may occur during the stirring process. For example, the stirring process may cause mechanical stress, which can result in crystal breakage or affect the formation of amorphous products. Besides, it is difficult to accurately control the stirring rate and time in practical operation. Hisaki et al. added a derivative of the organic ligand CPHAT to tetrahydrooromantan under alkaline conditions and reacted with stirring at 50 °C for 54 h. The product was then acidified and CPHAT was obtained.^[^ ^34^ ^]^ Xie et al. used a stirring method to rapidly synthesize HOF‐21, which was obtained by stepwise addition of Cu(NO_3_)_2_∙3HO and (NH_4_)_2_SiF_2_ to a mixture of acetonitrile/water in adenosine in an ethylene‐acid/water solution, which was then stirred for 3 h.^[^ ^89^ ^]^ Farha et al. dissolved H4TNAPy in DMF and acetone and stirred the solution to obtain HOF‐102 as a yellow powder after 12 h. The stirring method has a shorter synthesis time than the slow diffusion method, and the obtained crystals are more homogeneous but smaller.^[^ ^49^ ^]^

#### 3.1.6 Other Methods

Electrophoretic deposition (EPD) film‐forming is an emerging method for preparing charged‐particle films.^[^ ^79b^ ^]^ Hydrogen bonding on the surface of an HOF generates a surface charge on the donor, which can be exploited during EPD film‐forming. Cao and Liu's group chose pyrene‐based carboxylic acid ligands as the substrates for electrochromic materials and prepared rod‐like HOF nanomaterials (PFC‐1) at room temperature. They further prepared HOF films with a thickness of ≈500 nm on conductive glass by using electrophoretic film formation.^[^ ^90^ ^]^ The templating agent synthesis method also provides a novel mode of construction. Templating agents promote the self‐assembly of structures and achieve functionalization. Selecting suitable templating agents requires considering hydrogen bond donors, acceptors, and the desired features of self‐assembled structures. Liu et al. used the template method to prepare the first flexible polyHOF.^[^ ^91^ ^]^ The EPD method is still problematic in terms of the degree of thin‐film densification and the quality of the thin‐film crystals, whereas the template method has a cumbersome preparation procedure and uncontrollable thin‐film crystal quality.

In addition to the direct synthesis method mentioned above, indirect post‐synthesis can be used to form specific HOF structures by introducing specific functional groups and metal ions. Post‐synthesis modification of functionalized porous HOFs has been less studied due to the instability of hydrogen bonding. Chen's research team reported a 3D HOF‐19 and prepared HOF‐19⊃Pd(II) by post‐synthesis metal modification by adding HOF‐19 to an acetone solution containing the acetate target and immersing the HOF‐19 at room temperature for 12 h. The resulting HOF‐19⊃Pd(II) was then used as a catalyst.^[^ ^79a^ ^]^

### 3.2 Parameters and Challenges Related to Synthesis Methods

HOFs are solvated crystals that crystallize slowly in solvents, and this process is affected by factors such as the solvent, template, concentration, and temperature. It is challenging to optimize synthesis because it requires precise control over synthesis conditions (such as solvent polarity, concentration, temperature, and time). Common synthesis methods such as solvent volatilization, diffusion, solvothermal, recrystallization, and stirring methods have their own advantages and disadvantages. Obviously, all kinds of methods have their applicable scope and limitations, in practical application, it is necessary to choose the appropriate synthesis method according to the specific needs.

The factors affecting the synthesis of HOFs can be divided into intrinsic and extrinsic factors. Intrinsic factors involve the connection mode between a hydrogen bond donor and acceptor, and extrinsic factors include reaction temperature, concentration, template agent, pH value, etc. Slowing the nucleation rate generally promotes crystal growth, but liquid/vapor diffusion and evaporation/cooling methods are difficult to control. Solvothermal methods are the most commonly used synthesis methods and use a high temperature to promote the dissolution and self‐assembly of organic ligands to form thermodynamically stable crystals. The solubility can be improved and/or crystal precipitation can be accelerated by precisely controlling the temperature and pressure. In general, HOFs are self‐assembled from organic units with specific functional, wherein temperature and solvent are key factors determining their structure type and stability. Different synthesis methods may affect the synthesis precision and even form different configurations or transform structures by changing the extrinsic conditions, which makes it difficult to determine structure‐activity relationships.

Most existing synthetic methods for HOFs fall into the common category, and few studies have attempted to develop new methods adapted to multiple application scenarios and multifunctional modifications. Therefore, the synthesis of functionalized HOFs faces significant challenges given their unique nature and the limitations of synthetic methods. First, it is necessary to consider compatibility between functional groups and monomers to avoid side reactions or interference with backbone formation.^[^ ^86^ ^]^ Second, to maintain structural stability, the introduction of functional groups should not disrupt the solid backbone structure. Then, tailoring the pore environment and active sites is crucial to the applications of HOFs, but precisely controlling the distribution and orientation of functional groups presents a technical challenge. According to current research progress, compared with MOFs and COFs, HOFs have a significant shortcoming in terms of introducing functional sites.^[^ ^79^, ^92^ ^]^ Post‐modification methods are used in the catalytic field to introduce stronger coordination bonds into structures. Ensuring the accessibility and uniformity of the active sites is critical to achieving excellent performance. There are limitations in the applicability of current synthetic methods, such as solvent volatilization, diffusion, and solvothermal synthesis, which may not be compatible with specific functional groups and may lead to the decomposition of functional groups at high temperatures and pressures. Characterization and evaluation of functionalized HOFs are also more complex than those of non‐functionalized HOFs and require specific methods to confirm the introduction of functional groups and evaluate performance for specific applications.^[^ ^86^ ^]^ In addition, the scalability and industrial production of HOF synthesis methods is a major challenge involving multiple factors such as raw material costs, production equipment, reproducibility, and scalability. To overcome these challenges, it is necessary to explore new synthetic strategies, suitable functional groups, and advanced characterization techniques. It will also be necessary to strengthen interdisciplinary collaboration to promote the practical applications of functionalized HOFs in energy devices, environmental applications, and other fields.

## 4 Applications in the Field of Energy

Due to their easy synthesis, tunable structures, and high specific surface areas, HOFs play a unique role in energy storage, conversion, and generation, as well as separation.^[^ ^7b,c^ ^]^ In energy storage and conversion applications, HOFs can be used as electrodes in batteries and supercapacitors.^[^ ^93^ ^]^ HOFs’ high specific surface area and porosity facilitate electrolyte permeation and ion transport, thereby increasing the energy density and power density of the devices. The introduction of multiple hydrogen bonds and π–π conjugation improves the stability and conductivity of HOFs and enhances the performance of the resultant devices. HOF‐based materials can be used as catalysts for electrochemical energy conversion in various electrochemical processes, including the hydrogen evolution reaction (HER), oxygen evolution reaction (OER), and oxygen reduction reaction (ORR). In energy generation and separation applications, HOFs can be used as catalysts or adsorbents to generate or separate fuels such as CO, H_2_, NH_3_, formic acid, and alcohol.^[^ ^7^, ^94^ ^]^ Their porous structures and high specific surface area facilitate the adsorption of reactants and the separation of products, which ultimately improve catalytic/separation efficiencies. The porous structure can be tuned to achieve molecular size and polarity‐selective adsorption and separation. However, further research and optimization, such as improving the electrical conductivity, stability, and recyclability of HOFs, are needed to fully exploit the advantages of HOFs. Overall, the development of HOFs for electrochemical and energy applications will require an interdisciplinary approach. From the design and synthesis of materials and performance evaluation to the establishment of constitutive relationships and practical applications, HOF‐based materials will continue to develop rapidly. We expect to witness a revolution in the use of renewable energy led by HOFs.

### 4.1 Energy Storage and Conversion

#### 4.1.1 Energy Storage

##### Applications in Lithium‐Ion Batteries

**Figure 15:**
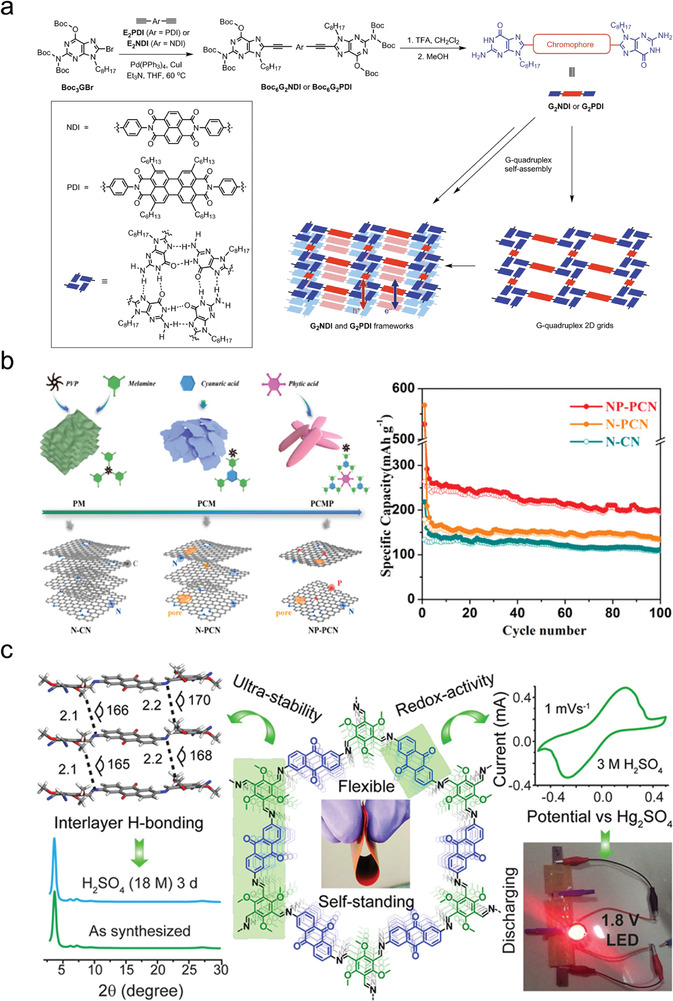
Examples of HOFs used for energy storage applications. a) Preparation of G‐quadruplex organic frameworks. Reproduced with permission.^[^ ^97^ ^]^ Copyright 2016, Springer Nature. b) Schematic of supermolecules assembled incrementally for the synthesis of SIB anode materials and the properties of the material. Reproduced with permission.^[^ ^100^ ^]^ Copyright 2022, American Chemical Society. c) Interlayer hydrogen‐bonded covalent organic frameworks as high‐performance supercapacitors. Reproduced with permission.^[^ ^104a^ ^]^ Copyright 2018, American Chemical Society.

Lithium‐ion (Li^+^) batteries are regarded as promising energy storage devices due to their high energy density and low costs.^[^ ^95^ ^]^ The high specific surface area of HOFs can provide more active sites for Li^+^ intercalation, but weak hydrogen bonding restricts their applications. Therefore, strategies such as multiple hydrogen bonding and π–π stacking have been used to enhance the backbone strength of HOFs to improve their solvent tolerance and electrochemical stability.^[^ ^96^ ^]^ Wu et al. designed two 2D organic frameworks materials (G2NDI and G2PDI) using the tetradentate G‐quartet as the electron donor, naphthalene‐1,4:5,8‐bis(dicarboximide) (NDI) or perylene diimide (2,5,8,11‐tetrahexylperylene‐3,4:9,10‐bis(dicarboximide) (PDI) as electron acceptors.^[^ ^97^ ^]^ As shown in **Figure** **15**a, G2NDI and G2PDI were synthesized through multiple hydrogen bonding and maintained high chemical stability in organic solvents due to multiple hydrogen bonding and intermolecular π–π stacking interactions. The battery capacity retention reached 99.97% per cycle. The unique lattice structure and multiple hydrogen bonding modes of HOFs are expected to improve the electrochemical stability of materials, which will contribute to the innovation of new energy technologies.

##### Applications in Sodium‐Ion Batteries

Sodium‐ion (Na^+^) batteries are produced at a lower rate than lithium‐ion batteries.^[^ ^98^ ^]^ The type and number of active sites by controlling HOFs synthesis can improve the adsorption of Na^+^, while the micro‐nanostructure and high porosity can achieve efficient Na^+^ transport. Wu et al. synthesized a 2D HOF‐DAT with good chemical stability based on multiple hydrogen bonds as a positive electrode for sodium‐ion batteries. It showed a high specific capacity and excellent cycling stability, and could stably cycle for 10 000 cycles at a current density of 1 A g^−1^.^[^ ^99^ ^]^ Density functional theory (DFT) calculations showed that the amino and oxygen atoms in DAT could stably adsorb Na^+^, which adsorbed and stored Na^+^ during charging and discharging. Zhu et al. obtained N and P co‐doped porous carbon nanosheets (Figure 15b) by the in situ self‐assembly and pyrolysis of HOFs precursors.^[^ ^100^ ^]^ By introducing defects and active sites, NP‐PCN showed a high specific capacity, improved performance, and excellent cycling stability. The current density at 100 mA g^−1^ reached 223 mAh g^−1^, and the capacity retention rate was 92.6% after 4000 cycles. Recently, Guo et al. designed structurally stable and aldehyde‐modified HOFs‐8 containing electronegative sites, which enabled the utilization of active sites and the rapid transport of sodium ions and electrons.^[^ ^101^ ^]^ HOFs‐8 electrodes could be recycled up to 5000 times at 3.66 A g^−1^ (20 °C). Since MOFs are formed by the self‐assembly of metal ions or metal clusters with organic ligands via coordination, their activity and stability can be improved by self‐fixing metal ions. In contrast, HOFs will require new composite strategies to improve the ionic mobility and cyclic stability of electrode materials.

##### Applications in Supercapacitors

Supercapacitors are high‐power, long‐life energy storage devices, with energy density typically lower than that of ion batteries but significantly higher power density. Supercapacitors can be used as complementary or in conjunction with batteries in the process of rapid charge, discharge, and transfer of energy.^[^ ^102^ ^]^ The porosity, specific surface area, and active sites of the electrode material play an important role in the capacitance, so selecting a suitable electrode material is crucial to ensuring the performance of a supercapacitor.^[^ ^103^ ^]^ Although HOFs have recently been used to develop supercapacitors due to their high porosity and structural tunability, their poor electrical conductivity and electrochemical stability need to be addressed.^[^ ^104^ ^]^ The hydrogen bonds in HOFs maintain appropriate spacing between two adjacent layers, avoid slipping between layers, maintain an ordered pore structure, and also facilitate rapid charge transfer. For example, Halder et al. constructed a REDOX‐active mesoporous HOF (TpOMe‐DAQ) with a high porosity (pore size of ≈2.3 nm and average specific surface area of 1531 m^2^ g^−1^) which exposed more charge‐adsorption sites and redox active sites. This gave the material an extremely high areal capacitance of 1600 mF cm^−2^ (Figure 15c).^[^ ^104a^ ^]^ The TpOMe‐DAQ layer was connected by C─H⋯N hydrogen bonds, which protected imine bonds in the structure from being cleaved in the acidic electrolyte, thus showing high stability. The Coulombic efficiency and capacitance of the obtained supercapacitor remained basically unchanged after 100 000 charge‐discharge cycles in 2 m H_2_SO_4_ electrolyte.

#### 4.1.2 Energy Conversion

##### Hydrogen Evolution Reaction

In the context of energy transformation, hydrogen production by electrolytic water splitting is an ideal way to produce green hydrogen. The development of low‐cost and highly active catalysts is the key to the large‐scale promotion of HER.^[^ ^105^ ^]^ HOFs contain a rich hierarchy of pore structures and active sites, and monomer regulation can introduce different heteroatoms as active centers to regulate Δ*G*_H_ and improve their HER performance.

**Figure 16:**
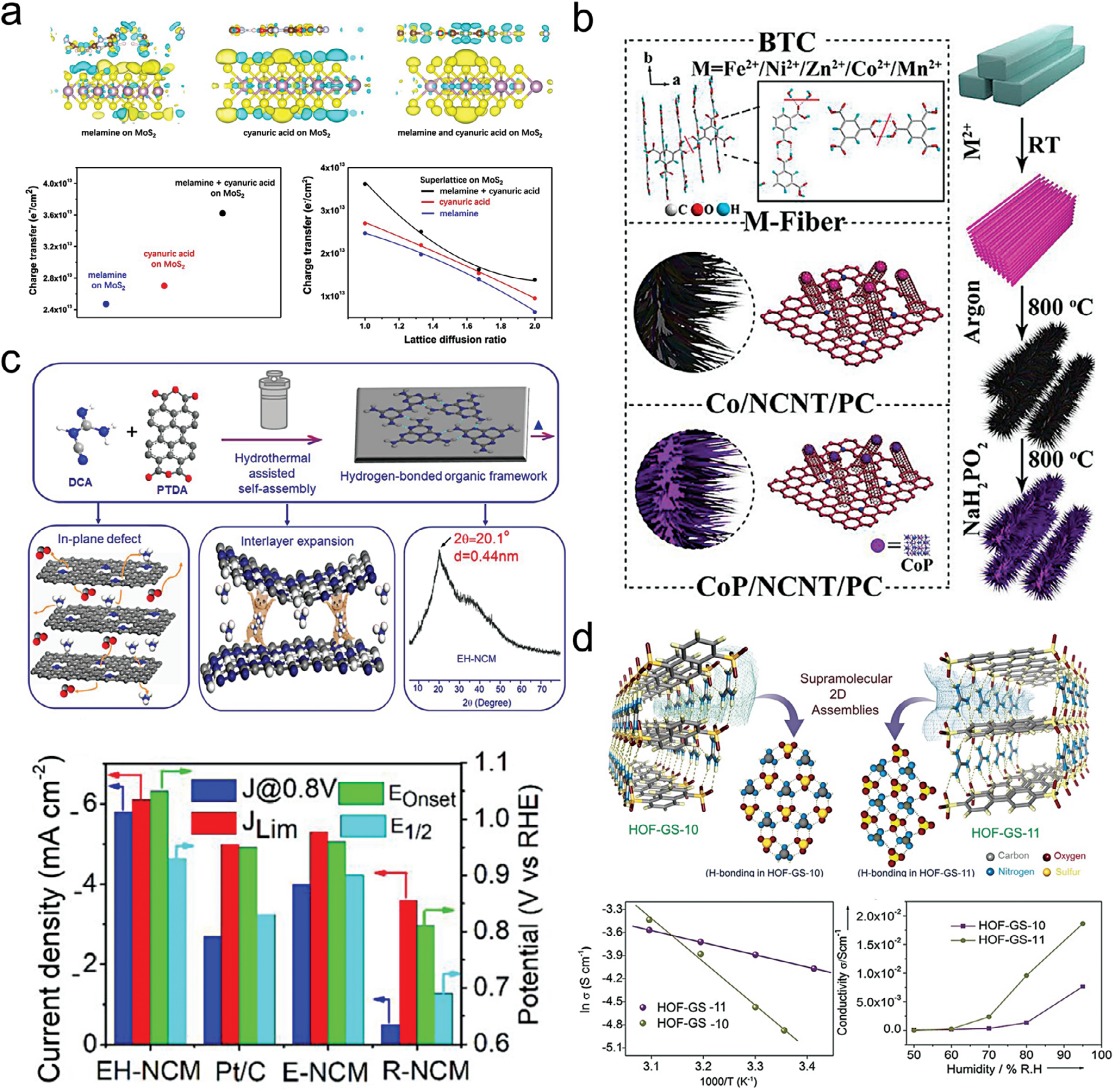
Examples of HOFs applications for energy conversion. a) Simulated charge transfer from melamine (M), cyanuric acid (CA), and a co‐assembled structure to a MoS_2_ monolayer. Reproduced with permission.^[^ ^107^ ^]^ Copyright 2023, Springer Nature. b) The schematic formation of CoP/NCNT/PC. Reproduced with permission.^[^ ^109^ ^]^ Copyright 2022, Elsevier. c) Synthesis route of EH‐NCM HOF and its properties. Reproduced with permission.^[^ ^111^ ^]^ Copyright 2021, Elsevier. d) Hydrogen‐bonded 2D frameworks of HOF‐GS‐10 and HOF‐GS‐11 and their properties. Reproduced with permission.^[^ ^27a^ ^]^ Copyright 2016, Wiley‐VCH.

Giri et al. prepared electrocatalysts (B.TC) from bipyridine b) and trithiocyanuric acid (TC) by hydrogen bond‐induced self‐assembly.^[^ ^106^ ^]^ The hydrogen‐bonded 3D multi‐channel structure and the S active sites introduced in TC decreased Δ*G*_H_ for B.TC and the free energy of the reaction 2H + 2e^−^. B.TC also possessed outstanding HER performance with a small overpotential of 80 mV, a Tafel slope of 78 mV/dec, an exchange current density of 0.047 mA/cm^2^, and long‐term durability. The weak conductivity of the HOF material itself could be improved by compounding with other materials. Wang et al. reported a stable non‐covalent architecture (CA·M) self‐assembled from melamine (M) and cyanuric acid (CA) to form a van der Waals heterostructure with MoS_2_ (CA·M/MoS_2_) (**Figure** **16**a).^[^ ^107^ ^]^ The n‐doping of the CA·M network and the transfer of electrons in the CA·M/MoS_2_ heterostructure increased the charge density on the surface of MoS_2_, which compensated for the poor conductivity of the HOF. The physisorption of a tightly‐packed 2D supramolecular network of CA·M onto the monolayer MoS_2_ channels produced an electron transfer up to 2.72 × 10^13^ cm^−2^. This was state‐of‐the‐art performance compared with molecule‐TMDCs hybrid devices.

##### Oxygen Evolution Reaction

The OER is a key half‐reaction during solar water decomposition, rechargeable metal‐air batteries, renewable fuel cells, electrolytic water hydrogen production, and other energy technologies. The design of efficient, durable, and low‐cost non‐precious metal catalysts is the focus of its large‐scale production. The unique pore channels and high specific surface area of HOFs, as well as tunable monomers, enable rich element doping and the introduction of more active sites, which help achieve rapid charge transfer processes and material transport. The results have shown that heteroatom doping can regulate the electronic structure of catalysts.

Wang et al. used ascorbic acid as a modulator to prepare S‐doped S@HOFH2BDC by a two‐step solvothermal method based on the hydrogen bonding of 1, 4‐benzenedicarboxylic acid (H2BDC).^[^ ^108^ ^]^ Comparing the Bode phase plot at 1.5 V before and after doping showed that the phase peak of S@HOF‐H2BDC appeared at a lower frequency and smaller phase angle. This confirmed that the S atom induced an electronic structure rearrangement, which accelerated the deprotonation of *OOH intermediates, thus promoting the overall OER kinetics. S@HOFH2BDC exhibited a low overpotential of 358 mV at a current density of 10 mA cm^−2^, which was superior to undoped HF‐H2BDC (406 mV) and almost comparable to that of commercial IrO_2_ (347 mV). The OER overpotential could be reduced by uniformly anchoring highly active metal ions in the macromolecular skeleton. Guo et al. added Co^2+^ ions to HOFs synthesized from homotriphthalic acid (1,3,5‐benzentric acid boxylicacid, H3BTC) (Figure 16b).^[^ ^109^ ^]^ After calcination and phosphating, Hough‐derived porous carbon‐coated particles obtained in CoP/NCNT/PC, and N‐doped multi‐walled carbon nanotubes were formed in situ. The material showed an overpotential of 282 mV at 10 cm^−2^, a small Tafel slope (103.7 mV dec^−1^), and long‐term durability of 90.5% over 10 h, with excellent OER properties. The method was simple, low cost, high efficiency, durable, and has the potential to replace the OER.

##### Oxygen Reduction Reaction

The ORR is the rate‐controlling step in reactions occurring in metal‐air batteries and fuel cells. The ORR includes the four‐electron reduction of O_2_ to H_2_O and the two‐electron reduction of H_2_O_2_. The reaction includes oxygen adsorption, dissociation, recombination, and desorption and involves the adsorption and coupling of various oxygen‐containing intermediates such as *O, *OH, and *OOH. Due to its slow kinetics, there is a need to develop efficient and stable ORR electrocatalysts. For example, some non‐metallic doped carbon materials with unique pore structures can accelerate electron transfer or facilitate the adsorption of oxygen atoms to promote the ORR.^[^ ^110^ ^]^

Using the hydrogen bond assembly‐pyrolysis strategy, Wang et al. synthesized an expanded interlayer and holey in‐plane nitrogen‐containing nanocarbon material^[^ ^111^ ^]^ that showed an initial potential of 1.05 V and a half‐wave potential of 0.93 V during ORR catalysis (Figure 16c). The calculations showed that the carbonization process of HOFs precursors produced abundant porous in‐plane defects. Combined with increased layer spacing, in‐plane defects combined with O_2_ to form a key oxygen‐containing intermediate OH, which was conducive to the four‐electron ORR. Zhang et al. constructed a novel hierarchical micro/mesoporous HOF via an assisted pyrolysis strategy and experimentally confirmed that the catalyst possessed excellent ORR performance, with a half‐wave potential as high as 0.895 V (vs RHE) and an overpotential as low as 356 mV under a current density of 10 mA cm^−2^.^[^ ^112^ ^]^ Molecular dynamics simulations further demonstrated that the microporous structure accelerated oxygen mass transfer and was determined to be the main reason for the enhanced ORR performance.

##### Fuel Cells

Fuel cells are promising clean energy conversion devices. The proton‐exchange membrane (PEM) is the main component of proton‐exchange membrane fuel cells (PEMFCs),^[^ ^113^ ^]^ but PEMs require complex preparation processes, are amorphous, and show low efficiencies. The donor‐acceptor structure of the building unit of HOFs can be used as either the proton source or proton carrier, and the hydrogen bond network provides a multi‐proton transport route. The flexibility and processability of HOFs open up new possibilities for thin‐film manufacturing.

Chen et al. prepared a porphyrin‐based polyporous HOF‐6 that showed excellent proton conductivity, whose 3D pore surface was exposed to functional porphyrin, which could be used as a proton donor–acceptor.^[^ ^114^ ^]^ At 97% RH and 300 K, HOF‐6a exhibited an excellent proton conductivity of 3.4 × 10^−6^ S cm^−1^. Recently, Karmakar et al. constructed HOF‐GS‐10 and HOF‐GS‐11 with a bilayer arrangement of biphenyl units and an extended hydrogen bond network of GS sheets. These HOFs showed multiple proton transport paths and were applied as proton conductors (Figure 16d).^[^ ^27a^ ^]^ At a low humidity (RH ≈ 60%), the proton conductivities of HOF‐GS‐10 and HOF‐GS‐11 were 1.78 × 10^−4^ and 2.6 × 10^−4^ S cm^−1^, respectively. Under high humidity (RH ≈98%), the maximum proton conductivity values of HOF‐GS‐10 and HOF‐GS‐11 were 0.75 × 10^−2^ and 1.8 × 10^−2^ S cm^−1^, respectively. The experimental results also showed that the activation energy (*E*_a_) values of HOF‐GS‐10 and HOF‐GS‐11 were 0.489 and 0.135 eV, respectively. This study confirmed that the proton conductivity of HOF‐based materials may exceed that of other porous crystal materials such as MOFs and COFs under certain humidity conditions. Recently, Chen et al. designed HOF‐FJU‐36 with proton‐electron transfer coupling. HOF‐FJU‐36 used zwitterionic 1,1′‐bis(3‐carboxybenzyl)−4,4′‐bipyridinium (H_2_L^2+^) as the acceptor and 2,7‐naphthalene disulfonate (NDS^2−^) as the acceptor to form a 2D layered structure by π–π stacking.^[^ ^115^ ^]^ Due to the coupled electron–proton transfer property, it showed photoresponsive electron and proton conductivity under 405 nm light irradiation.

### 4.2 Generation and Separation of Energy Materials

#### 4.2.1 Energy Material Generation

##### Conversion of CO_2_ to CO

Photocatalysis converts solar energy into chemical energy, but traditional photocatalysts have disadvantages such as a low utilization of visible light, high toxicity, and strong photo‐corrosivity.^[^ ^92^ ^]^ The structural tunability and functional diversity of HOFs give them potential applications for regulating photocatalytic performance, as well as for improving light capture and charge transfer and the integration of catalytically active sites. Recently, the unique properties of HOFs have been applied in the photocatalytic production of energy substances.^[^ ^92^, ^116^ ^]^

**Figure 17:**
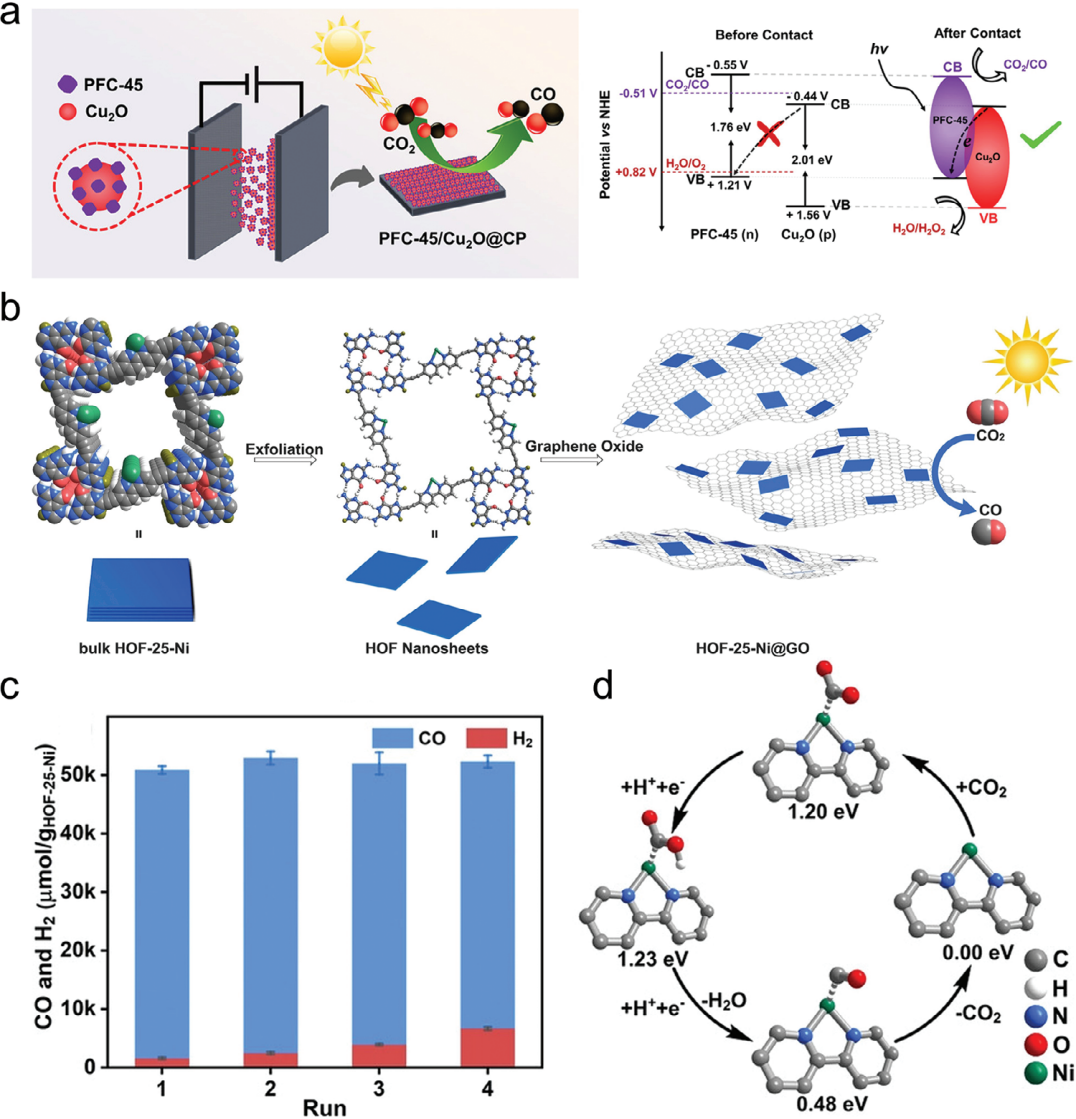
Examples of the conversion of CO_2_ to CO. a) Construction process of PFC‐45/Cu_2_O@Cp and schematic diagram of charge transfer between p‐type Cu_2_O and n‐type PFC‐45. Reproduced with permission.^[^ ^116a^ ^]^ Copyright 2022, American Chemical Society. b) Schematic diagram of the synthesis of HOF‐25‐Ni@GO for photocatalytic conversion of CO_2_ to CO. Reproduced with permission.^[^ ^116b^ ^]^ Copyright 2022, Wiley‐VCH. c) Circularity test of HOF‐25‐Ni@GO‐10 photocatalyst. Reproduced with permission.^[^ ^116b^ ^]^ Copyright 2022, Wiley‐VCH. d) Schematic of the mechanistic route for the HOF‐25‐Ni‐catalyzed CO_2_ reduction. Reproduced with permission.^[^ ^116b^ ^]^ Copyright 2022, Wiley‐VCH.

PFC‐45/Cu_2_O@CP films deposited on the electrodes of carbonate paper (CP) were synthesized by in situ electrophoretic deposition (EPD) by Cao et al.^[^ ^116a^ ^]^ A p‐n heterojunction was formed at the binding interface of PFC‐45 and Cu_2_O, which promoted the separation of electrons and holes and inhibited the recombination of excited charges, so the electrons in the CB of PFC‐45 with a sufficient negative potential efficiently reduced CO_2_ to CO (**Figure** **17**a). Chen et al. prepared HOF‐25‐Re loaded with Re(CO)_5_Cl for photocatalytic CO_2_ reduction. This material showed strong stability, high porosity, clear charge transfer pathways, and uniform metal sites.^[^ ^92^ ^]^ After adding the photosensitizer [Ru(bpy)_3_]Cl_2_∙6H_2_O to the system, the photocatalytic conversion of CO_2_ to CO by HOF‐25‐Re lasted for 16 hours (8 cycles). The conversion rate of CO_2_ to CO reached as high as 1448 µmol g^−1^ h^−1^ with a selectivity of 93%. After the deactivation of HOF‐25‐Re, its photocatalytic properties were recovered by recrystallization and post‐modification. Jiang's research group further improved the photocatalytic performance of HOF‐25 by preparing 2D nanomaterials (Figure 17b).^[^ ^116b^ ^]^ HOF‐25‐Ni 2D nanosheets showed a higher specific surface area, stronger charge conductivity, and size‐dependent properties, and adhered to GO through strong π–π stacking to accelerate internal electron transfer (Figure 17b,d). The CO_2_ reduction data showed that the photocatalytic CO_2_‐to‐CO conversion rate of HOF‐25‐Ni@GO nanosheets reached 24 323 µmol g^−1^ h^−1^, which was significantly higher than that of the above‐mentioned HOF‐25‐Re (Figure 17c). HOFs provide a unique choice for the development of efficient and reusable photocatalysts.^[^ ^116c^ ^]^ To enhance their electrochemical activity, MOFs are usually combined with metal/metal oxides, organic molecules, and polyoxometalates to construct MOF‐based composites.^[^ ^117^ ^]^ MOFs/COFs provide spaces to enable the confinement of greater concentrations of CO_2_ near encapsulated active sites to obtain a high activity, but HOFs still face great challenges in this regard.

##### Hydrogen Production

**Figure 18:**
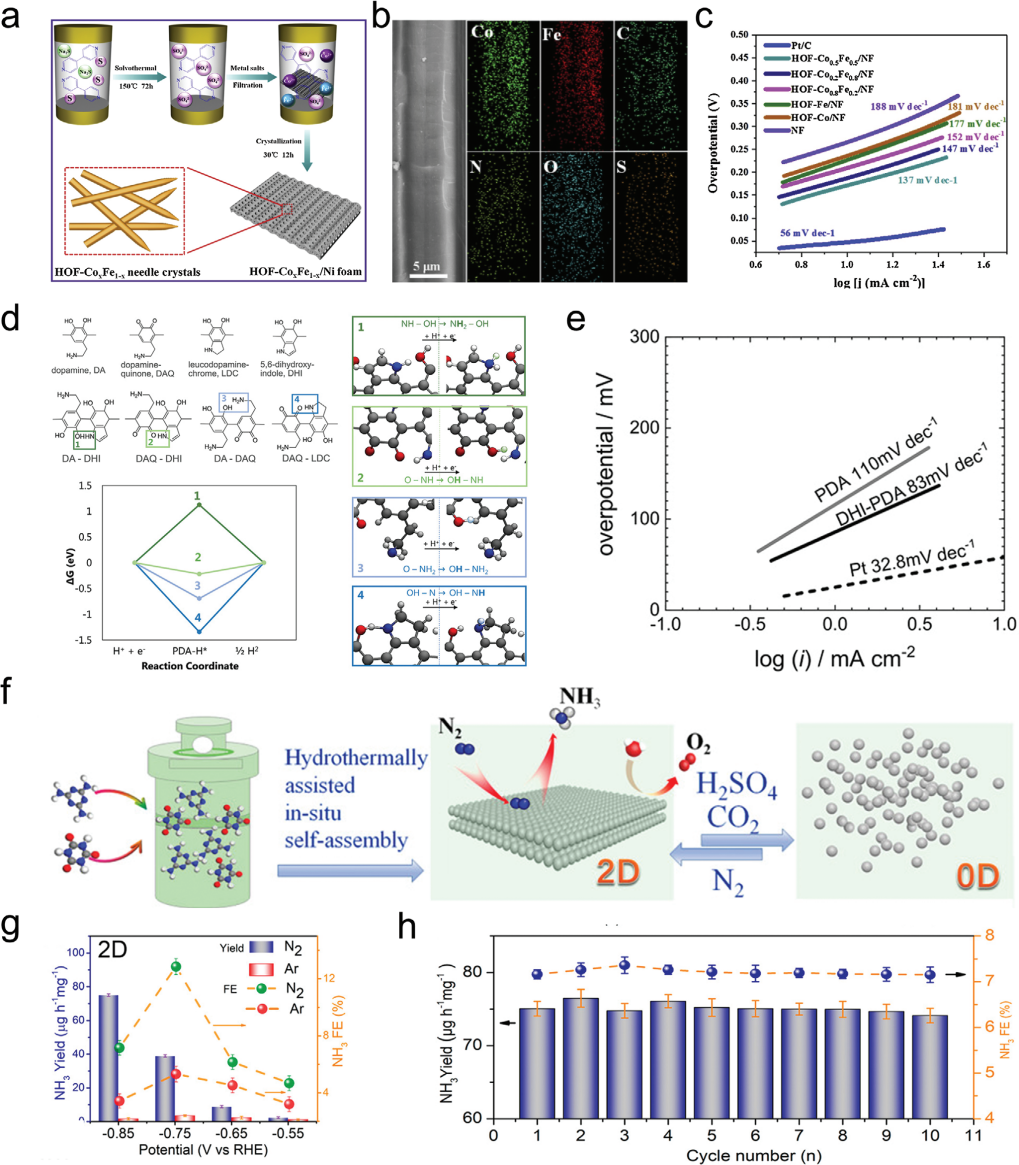
Examples of energy materials generation. a) A proposed draft of the step‐by‐step approach for in situ growing bimetal‐complex‐based HOF materials on Ni foam. Reproduced with permission.^[^ ^74^ ^]^ Copyright 2019, Elsevier. b) SEM‐EDS maps of HOF─Co_0.5_Fe_0.5_. Reproduced with permission.^[^ ^74^ ^]^ Copyright 2019, Elsevier. c) Polarization curves. Reproduced with permission.^[^ ^74^ ^]^ Copyright 2019, Elsevier. d) DFT calculations of the hydrogen affinity of hydrogen bonding. Reproduced with permission.^[^ ^119^ ^]^ Copyright 2019, Wiley‐VCH. e) Tafel plot (log i) vs η) with the slopes indicated in the graph. Reproduced with permission.^[^ ^119^ ^]^ Copyright 2019, Wiley‐VCH. f) Sketch of the preparation and properties of 2D CNQDs. Reproduced with permission.^[^ ^120^ ^]^ Copyright 2022, American Chemical Society. g) Averaged ammonia yield rate and Faradaic efficiency of 2D CNQDs. Reproduced with permission.^[^ ^120^ ^]^ Copyright 2022, American Chemical Society. h) NH_3_ yield rates at −0.85 V in N_2_‐saturated electrolyte. Reproduced with permission.^[^ ^120^ ^]^ Copyright 2022, American Chemical Society.

Electrochemical hydrogen production via the HER is a common method to obtain clean energy. Due to the difficulty of large‐scale application of platinum catalysts, it is urgent to explore high‐performance and low‐cost electrocatalysts.^[^ ^118^ ^]^ The special structural properties of HOFs allow them to be precisely regulated (loading metal atoms to increase catalytic active sites or increasing functional groups to improve catalytic selectivity). Liu et al. first synthesized HOFs (HOF─CO) based on metal complexes by a stepwise method and then crystallized bimetal HOF (HOF─Co_x_Fe_1‐x_) on nickel foam (**Figure** **18**a,b).^[^ ^74^ ^]^ The bimetal HOF─Co_0.5_Fe_0.5_ grown in situ on nickel foam showed excellent HER properties under alkaline conditions. After 1000 CV cycles, the polarization curves of HOF─Co_0.5_Fe_0.5_/NF were measured again and the curves nearly completely coincided, thus verifying its stability (Figure 18c). Recently, Coskun et al. reported a metal‐free hydrogen‐bonded polymer that mimicked a precious metal electrocatalyst.^[^ ^119^ ^]^ This study explored conductive polydopamine (PDA) as an electrocatalyst whose organic surface provided excellent catalytic activity through structural design and adjustment of its electrical conductivity. First, the atomic number calculation method was used to analyze the different types of H bonds in PDA. The DFT calculations showed that ketoindoline functional groups could be introduced several times into the PDA structure to improve its HER selectivity (Figure 18d). When the current density was 10 mA cm^−2^, the potential required for DHI‐PDA was lower than that of standard PDA*, and the Tafel slope (80 mVdec^−1^) of DHI‐PDA was lower than that of PDA* (110 mVdec^−1^), indicating better electrocatalytic performance (Figure 18e). Although the electrocatalytic system broadened the application platform, its related mechanism and acid stability still need to be further studied.

##### Ammonia Production

Ammonia has a very high energy density, and its combustion releases a lot of heat. The only products are water vapor and nitrogen, giving it zero carbon emissions. Driven by climate change, energy transition, and carbon neutrality, NH_3_ is an ideal clean energy substance. Wang et al. prepared carbon nitride‐based quantum dots (CNQDs) using the HOF precursor system through an in situ hydrothermal supramolecular self‐assembly approach (Figure 18f).^[^ ^120^ ^]^ CNQDs were constructed by the in situ arrangement of transverse hydrogen bonds along two orthogonal directions to construct free‐floating 2D CNQD films. Using the stimulus‐responsiveness and reversibility of hydrogen bonds, the controlled assembly–disassembly of 2D CNQD films was achieved by injecting external stimuli such as CO_2_/N_2_, which gave the assembled 2D CNQD films better electrochemical performance than other 2D films and 0D quantum dots. The 2D CNQD films showed excellent bi‐functional activity in nitrogen reduction reaction (NRR) and OER. During the nitrogen reduction reaction, the NH_3_ yield of the film reached 75.07 µg h^−1^ mg^−1^ at −0.85 V (Figure 18g). 2D CNQDs promoted the adsorption of N_2_ and the stabilization of NRR intermediates under the coordination of polyhydrogen bond interactions, which was the main reason for the electrocatalytic performance of NRR. Recently, Wang et al. pre‐screened 36 HOFs with different metals as active sites through high‐throughput calculations for the nitrate reduction reaction (NO_3_RR).^[^ ^121^ ^]^ Their results confirmed that the electrocatalyst (HOF‐M1) reduced nitrate to ammonia, which can be used to treat wastewater pollution while also synthesizing ammonia.

##### Formic Acid Production

Formic acid is an important organic compound that is widely used in the chemical industry, medicine, and agriculture industry.^[^ ^122^ ^]^ However, it has low catalyst activity, poor selectivity, and environmental pollution during its preparation. Thus, new preparation methods for formic acid should be developed.

**Figure 19:**
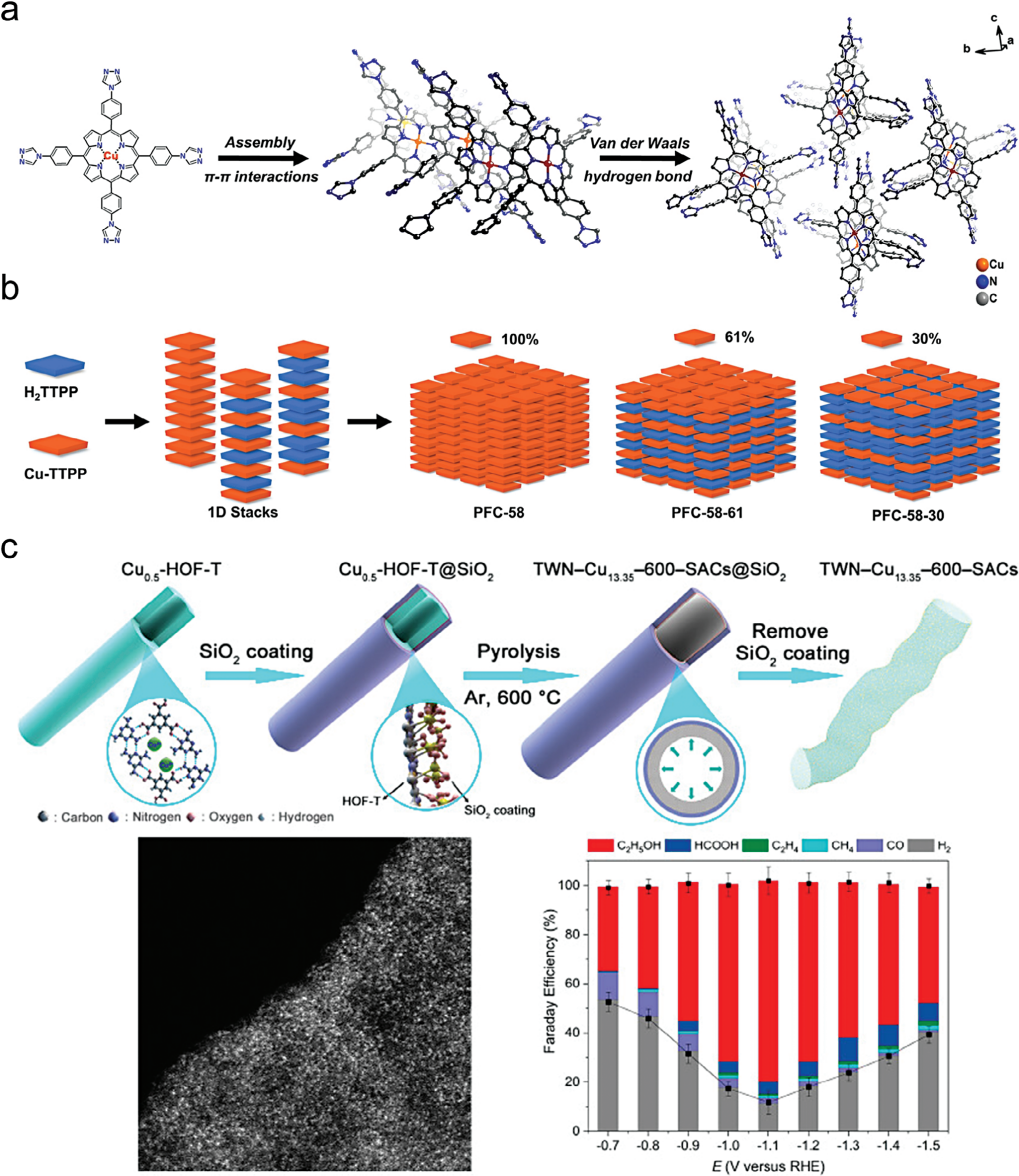
Examples of energy material generation. a) Crystal structure of PFC‐58 is composed of porphyrin building blocks with the AA packing mode. Reproduced with permission.^[^ ^123^ ^]^ Copyright 2022, Wiley‐VCH. b) Schematic diagram of the structures of PFC‐58 series HOFs with different metalloporphyrin contents. Reproduced with permission.^[^ ^123^ ^]^ Copyright 2022, Wiley‐VCH. c) Preparation and characterization of catalysts. Reproduced with permission.^[^ ^125^ ^]^ Copyright 2023, American Chemical Society.

Recently, Cao et al. developed a bionic heterogeneous catalyst by self‐assembling metal porphyrin monomers into a high‐porosity HOF (**Figure** **19**a).^[^ ^123^ ^]^ In this prototype structure, the photosensitizer‐to‐catalyst ratio could be adjusted only by changing the ratio of metalized porphyrin centers. By controlling the ratio of the loaded Cu‐TTPP and metal‐free H2TTPP in the reaction mixture, a series of isostructural HOFs of porphyrin‐centered metalized Cu (100%, 61%, and 30%) was prepared and named PFC‐58, PFC‐58‐61, and PFC‐58‐30 (Figure 19b). Changing the metal content of porphyrins fine‐tuned the photosensitizer‐to‐catalyst ratio and significantly changed the microenvironment around the active sites, such as the electron density, base‐frame interaction, and redox potential, which changed the separation efficiency. As a result, the catalytic activity of these HOFs during the reduction of CO_2_ in the presence of H_2_O vapor varied greatly. Among them, PFC‐58‐30 with the lowest metal content had the best performance, and the HCOOH production rate reached 29.8 µmol g^−1^ h^−1^ without using any sacrificial agent or cocatalyst, which has obvious advantages under similar conditions.

##### Production of Alcohols

Alcohol energy substances are mainly obtained through biomass production or fossil fuel conversion, the latter of which has limited reserves and produces serious environmental problems. Thus, the use of renewable resources to produce alcohol energy sources has become a research hotspot.^[^ ^124^ ^]^

Wang et al. reported an example of an ionic hydrogen‐bond organic framework (iHOF) constructed from guanidine cations and boric acid anions ([B(OCH_3_)_4_]_3_[C(NH_2_)_3_]4Cl‐4CH_3_OH, Gd‐B) that reversible and automatically adsorbed/released methanol (MeOH) under ambient conditions.^[^ ^72^ ^]^ The metastable Gd‐B automatically released all sixteen MeOH molecules (63.4 wt.%) via desorption and tetra‐methyl borate hydrolysis in an ambient environment. The structure was recovered when re‐exposed to MeOH vapor or liquid, thus mimicking combustible ice behavior but at ambient conditions. The reversible capture/release of four guest MeOH molecules was also realized without destroying its crystal structure. Xia et al. recently proposed a silicon dioxide (SiO_2_)‐mediated HOF template method to prepare ultra‐high‐density Cu single‐atom catalysts (SACs) on thin‐walled N‐doped carbon nanotubes with a Cu content of up to 13.35 wt.% (Figure 19c).^[^ ^125^ ^]^ The catalyst showed excellent performance for the electrochemical conversion of CO_2_RR to ethanol (with an H‐type battery, current density 35.6 mA cm^−2^, and the FE of ethanol reached 81.9%).

#### 4.2.2 Separation of Energy Materials

##### Carbon Monoxide Separation

Industrial CO production by steam reforming or water vapor conversion is not only energy intensive but also requires challenging CO_2_/CO separation. Recently, MOFs containing metal ions can achieve CO_2_─CO separation, but MOFs are often very sensitive to humidity, and show a sudden drop in their gas adsorption capacity or structural disintegration under high humidity or acidic gas conditions.^[^ ^126^ ^]^ HOFs are generally more stable in water or humid environments due to the hydrophobicity of pure organic molecules. Regulating the pore size and hydrophobicity of HOFs is expected to help identify an ideal adsorbent for industrial CO_2_/CO separation.

**Figure 20:**
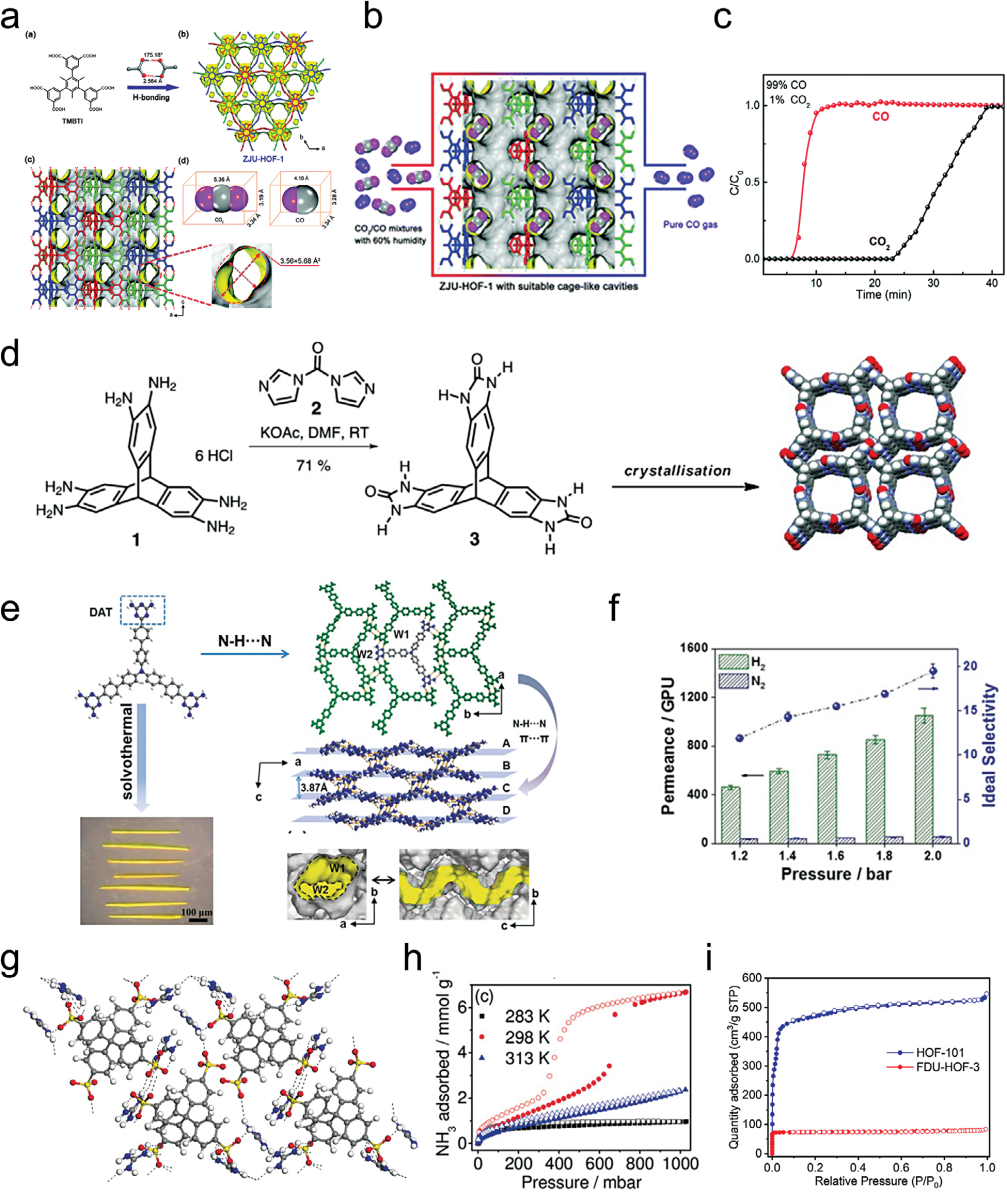
Examples of gas separation. a) Crystal structure of ZJU‐HOF‐1 and a comparison of the molecular size of CO_2_ and CO. Reproduced with permission.^[^ ^127^ ^]^ Copyright 2021, Royal Society of Chemistry. b) Schematic diagram of CO_2_ and CO separation. Reproduced with permission.^[^ ^127^ ^]^ Copyright 2021, Royal Society of Chemistry. c). Experimental breakthrough curve for a dry CO_2_/CO (1/99) mixture. Reproduced with permission.^[^ ^127^ ^]^ Copyright 2021, Royal Society of Chemistry. d) Synthesis of triptycene trisbenzimidazolone 3 (TTBI). Reproduced with permission.^[^ ^20a^ ^]^ Copyright 2012, Wiley‐VCH. e) Structure of UPC‐HOF‐6. Reproduced with permission.^[^ ^21b^ ^]^ Copyright 2019, Wiley‐VCH. f) Single‐gas permeation of a UPC‐HOF‐6‐120 membrane at 25 °C and variable transmembrane pressure drops. Reproduced with permission.^[^ ^21b^ ^]^ Copyright 2019, Wiley‐VCH. g) Network structure showing multiple hydrogen bonds (dotted lines) between the ‐NH groups of GuaH^+^ and the O atoms of SPM4^−^ in KUF‐1a. Reproduced with permission.^[^ ^64a^ ^]^ Copyright 2019, Wiley‐VCH. h) NH_3_ isotherms of KUF‐1 at the indicated temperatures.^[^ ^64a^ ^]^ Reproduced with permission.^[^ ^64a^ ^]^ Copyright 2019, Wiley‐VCH. i) Comparison of the ammonia capture performance of FDU‐HOF‐3 and HOF‐101. Reproduced with permission.^[^ ^131^ ^]^ Copyright 2024, American Chemical Society.

Qian's research group reported the first microporous HOF material (ZJU‐HOF‐1) with an appropriate pore size under dry and wet conditions, which showed suitable pore closure, high chemical stability, and ultra‐low water absorption, as well as efficient CO_2_/CO separation (**Figure** **20**a,b).^[^ ^127^ ^]^ A ZJU‐HOF‐1 framework was constructed from TMBTI ligands through hydrogen bonding interactions to form a triple‐interpenetrating 3D network. The triple‐interpenetrating and rod‐like stacking configuration produced cage pockets between adjacent interpenetrating networks. The oval window of this pocket showed a pore size (3.56 Å × 5.68 Å) that better matched the size of CO_2_ (3.19 Å × 3.34 Å × 5.36 Å) and CO molecules (3.28 Å × 3.34 Å × 4.18 Å). As expected, this HOF exhibited highly efficient CO_2_/CO separation under dry and wet conditions (60% RH), with a maximum CO_2_/CO selectivity of 8.7 (Figure 20c).

##### Hydrogen Separation

Hydrogen is at risk of leakage and explosion during storage and transportation, so the development of efficient and safe hydrogen separation technology is important for its applications.^[^ ^128^ ^]^ In 2012, Mastalerz et al. prepared a HOF material (TTBI) with an ultra‐high specific surface area based on U1 building units (Figure 20d).^[^ ^20a^ ^]^ The TTBI material showed two kinds of channels, one of which was circular (≈ 1.45 nm in diameter) and the other of which was narrow (0.38 nm × 0.58 nm). The TTBI material formed a permanent channel, and its frame did not collapse after the solvent in the channel was removed. The TTBI material's porosity reached 60%, its pore volume reached 1.02 cm^3^ g^−1^, and its BET‐specific surface area reached 2796 m^2^ g^−1^. Because the TTBI material had a very high specific surface area, it was used for adsorption and storage of H_2_ and CH_4_. At 77 K and 1 × 10^5^ Pa, TTBI had a hydrogen adsorption capacity of 10.8 mL g^−1^ (a mass adsorption capacity of 2.2%), which was the largest hydrogen adsorption capacity ever reported for a HOF.

HOFs combine the solubility of polymers with the porosity of ordered porous materials and can be made into membranes such as polymers due to their solution processability while retaining excellent gas/liquid permeability and selectivity.^[^ ^129^ ^]^ Recently, Sun et al. reported the first example of a microporous HOF(UPC‐HOF‐6) (Figure 20e) membrane constructed from 4′,4′,4″‐nitrilotris(((1,1′‐biphenyl]−4‐diaminotriazine)) (NBP – DAT).^[^ ^21b^ ^]^ The narrowest channel of the UPC‐HOF‐6 was ≈2.8 Å and was suitable for separating H_2_ from other gases. At 80 °C, the UPC‐HOF‐6 dimethyl sulfoxide (DMSO) solution (120 g L^−1^) was coated on the porous Al_2_O_3_ substrate surface and slowly vaporized to prepare a continuous UPHOF‐6‐120 film. The selectivity of H_2_/N_2_ reached 19.5 (Figure 20f). The UPCHOF‐6‐120 film also showed excellent mechanical and thermal stability, and the scratched film healed easily after being treated with DMSO steam at 120 °C.

##### Ammonia Separation

Ammonia is an important industrial raw material and toxic gas, so it is important to develop porous materials that can selectively adsorb ammonia. Porous materials such as MOFs, COFs, and CMPs have been used as NH_3_ adsorbents.^[^ ^130^ ^]^ However, the development of ideal adsorbents that can be regenerated under mild conditions remains an important task. Hong et al. reported the first HOF material (KUF‐1) with NH_3_ adsorption properties,^[^ ^64a^ ^]^ which was constructed from S3+G to form a 3D framework (Figure 20g). It did not adsorb N_2_, H_2_, or O_2_, but it adsorbed NH_3_. At 283 K and 1 × 10^5^ Pa, KUF‐1a showed a typical type I adsorption isotherm, and its adsorption capacity reached 0.97 mmol g^−1^. At 298 K, the isotherm became S‐shaped, and its adsorption capacity rapidly increased from 3.41 mmol g^−1^ at 6.5 × 10^4^ Pa to 6.67 mmol g^−1^ at 1 × 10^5^ Pa (Figure 20h). This S‐shaped isotherm gave KUF‐1a a significant working capacity and was easily regenerated and recyclable at room temperature. Upon increasing the NH_3_ adsorption capacity, the cell volume of KUF‐1a decreased from 2.0057 to 1.9891 nm^3^, and the volume was recovered after desorption. It is speculated that NH_3_ was mainly adsorbed on the surface of the KUF‐1a crystal at 283 K. At 298 K, NH_3_ entered the KUF‐1a crystal and formed multiple hydrogen bonds with the framework, eventually exhibiting a V‐shaped adsorption isotherm. After five consecutive adsorption cycles at room temperature, the adsorption capacity of NH_3_ did not decrease, thus giving KUF‐1a the potential to be applied for NH_3_ adsorption. Recently, Song et al. reported that a novel and self‐healing microporous HOF could trap NH_3_ efficiently.^[^ ^131^ ^]^ Compared with existing mesoporous HOF‐101, FDU‐HOF‐3 showed a record‐breaking ability to trap low concentrations of NH_3_ (8.13 mmol g^−1^ at 25 mbar) (Figure 20i). This self‐healing HOF trapped and released NH_3_ for more than ten cycles, showing stable adsorption.

##### Methane Separation

Natural gas is mainly composed of CH_4_ and is a clean energy alternative to fossil fuels due to its lower carbon emissions and higher energy density. Common impurities in natural gas (C_2_H_2_ and CO_2_) reduce its energy level and also seriously corrode transportation pipelines. The use of energy‐saving separation technologies to purify CH_4_ is important for economic and safe natural gas extraction. Acetylene is a raw material used to produce organic products, but the efficient purification of methane and C_2_H_2_ is challenging.

**Figure 21:**
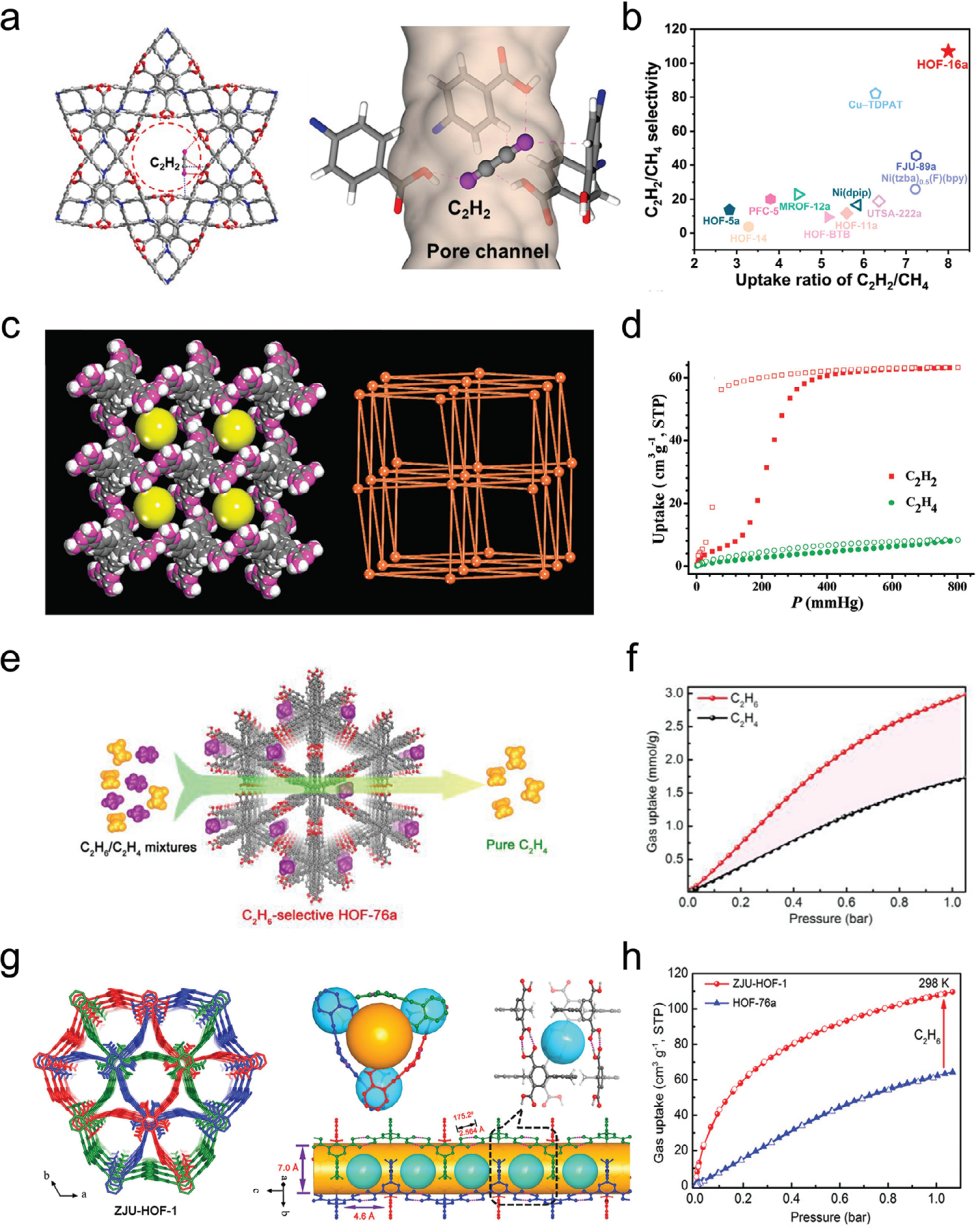
Examples of gas separation. a) The binding site of C_2_H_2_ in the HOF‐16a channel (left) and interaction with the adjacent ─COOH group on the channel surface (right). Reproduced with permission.^[^ ^132^ ^]^ Copyright 2023, Elsevier. b) CO_2_/CH_4_ selectivity of HOF‐16a and HOF‐11a at 298 K (50/50, v/v). Reproduced with permission.^[^ ^132^ ^]^ Copyright 2023, Elsevier. c) 1D channel of HOF‐1, with a size of 8.2 Å (yellow sphere) and a 3D network topology. Reproduced with permission.^[^ ^19^ ^]^ Copyright 2011, American Chemical Society. d) C_2_H_2_ and C_2_H_4_ sorption isotherms at 273 K. Reproduced with permission.^[^ ^19^ ^]^ Copyright 2011, American Chemical Society. e) Crystal structure of HOF‐76 and f) Adsorption isotherms of C_2_H_6_ (red) and C_2_H_4_ (black) for HOF‐76a at 296 K. Reproduced with permission.^[^ ^138^ ^]^ Copyright 2020, American Chemical Society. g) Detailed structural information of ZJU‐HOF‐1. Reproduced with permission.^[^ ^81^ ^]^ Copyright 2021, Wiley‐VCH. h) Comparison of C_2_H_6_ adsorption isotherms for ZJU‐HOF‐1 and HOF‐76a at 298 K. Reproduced with permission.^[^ ^81^ ^]^ Copyright 2021, Wiley‐VCH.

Yang et al. reported the synthesis of porous HOF SOF 1a whose BET surface area was 474 cm^2^ g^−1^. It absorbed 69 cm^3^ g^−1^ CH_4_ at 195 K and 1 bar.^[^ ^26a^ ^]^ Mastalerz et al. reported a HOF called TTBI (also named T2‐α by Pulido et al. ^31^) with a BET surface area of 2796 m^2^ g^−1^ that could adsorb 21 cm^3^ g^−1^ CH_4_ at 1 bar and 273 K.^[^ ^20a^ ^]^ Following the ESF maps, Pulido et al. predicted that T2 could form another porous phase (T2‐γ) with a larger BET surface area, lower density, and higher CH_4_ delivery capacity than TTBI.^[^ ^20b^ ^]^ T2‐γ exhibited a record‐high BET surface area of 3425 m^2^ g^−1^ among HOF materials to date. The saturation CH_4_ uptake capacity at 115 K for T2‐γ was 47.4 mol kg^−1^. Yang et al. introduced a 3D HOF‐11 created by a slow diffusion method with a surface area of 687 m^2^ g^−1^.^[^ ^28^ ^]^ At 1 atm and 296 K, the absorption of CH_4_ by HOF‐11a was 8 cm^3^ g^−1^, while At 273 K, the CH_4_ absorption increased to 13 cm^3^ g^−1^. Recently, Cai et al. demonstrated that HOF‐16 could separate for C_2_H_2_/CH_4_ and CO_2_/CH_4_ mixtures. HOF‐16 showed an affinity for C_2_H_2_ due to its exposed free ─COOH sites in its pore channels (**Figure** **21**a).^[^ ^132^ ^]^ The gas sorption isotherms revealed that HOF‐16a created a high C_2_H_2_/CH_4_ uptake ratio of 7.9 at 298 K and 1 bar, resulting in a C_2_H_2_/CH_4_ separation selectivity of 107 (50/50, v/v) under ambient conditions, which is a record among HOFs and even exceeded that of many MOFs (Figure 21b). HOF‐16a efficiently extracted C_2_H_2_ from the adsorption bed and purified CH_4_ (purity > 99.9%) with a low energy consumption. Liu et al. reported the first CMS membrane derived from crystalline porous HOF, named HCMS.^[^ ^133^ ^]^ The high selectivity and stability were experimentally confirmed, where the CO_2_/CH_4_ selectivity of the HCMS membrane after pyrolysis at 600 °C was 128, and the stable separation performance was maintained after physical aging. The results of this work open the door for the construction of CMS membranes using crystalline porous membrane precursors.

##### Hydrocarbon Separation

Light hydrocarbons (C_2_H_2_, C_2_H_4_, C_2_H_6_, etc.) are important energy substances and chemical raw materials. HOFs are green separation materials that may be suitable for the adsorption/separation of C2 light hydrocarbons. Low concentrations of C_2_H_2_ can poison catalysts and form explosive metal acetylides, so removing trace amounts of acetylene from ethylene is important for the production of ethylene downstream products.^[^ ^5^, ^134^ ^]^ Chen and collaborators synthesized a 3D microporous HOF with permanent pores that could selectively separate C_2_H_2_/C_2_H_4_ under ambient conditions (Figure 21c).^[^ ^19^ ^]^ At 196 K, the CO_2_ adsorption isotherm showed that HOF‐1a exhibited permanent porosity. At 273 K, the adsorption capacity of HOF‐1a for C_2_H_2_ was 63.2 cm^3^g^−1^ (STP) (Figure 21d), while the adsorption capacity for C_2_H_4_ was only 8.3 cm^3^g^−1^ (STP), and the selectivity of C_2_H_2_/C_2_H_4_ was 7.6. Chen et al. synthesized a microporous hydrogen‐bonded metal complex with permanent porosity or a metal conduit framework called HOF‐21.^[^ ^135^ ^]^ HOF‐21 showed permanent porosity and excellent C_2_H_2_/C_2_H_4_ selective separation at room temperature attributed to the hydrogen bonding interactions of C_2_H_2_ and SiF_6_^2−^ superimposed at both ends. More importantly, HOF‐21 was restored to its original structure in water or anionic aqueous solution. Due to its superior water stability and recoverability, HOF‐21 shows potential real‐world industrial applications. Recently, a sticked‐layer strategy was proposed to construct a flexible‐robust HOF, HOF‐FJU‐8, from a donor d)–π–acceptor a) molecule 4,4′,4″,4‴‐(pyrrolo[3,2‐b]pyrrole‐1,2,4,5‐tetrayl)tetrabenzonitrile (DP‐4CN).^[^ ^136^ ^]^ HOF‐FJU‐8a was flexible and robust and also featured a high C_2_H_2_/CO_2_ selectivity (3.9) for equimolar gas mixtures, which was superior to HOF‐15 (1.8) and many MOFs, including FJU‐6‐TATB (3.1) and JXNU‐11 (Fe2Ni) (2.7).

The C_2_H_4_ production also produces the impurity C_2_H_6_, and the separation of C_2_H_4_/C_2_H_6_ is a necessary but challenging process in the petrochemical industry.^[^ ^137^ ^]^ Chen et al. designed HOF‐76 with 1D triangular channel‐like pores with a diameter of 7.0 Å (Figure 21e).^[^ ^138^ ^]^ The non‐polar/inert pore surface of activated HOF‐76a enabled it to adsorb more C_2_H_6_ than C_2_H_4_, with a separation selectivity of up to 1.4 (Figure 21f), which was superior to that of most reported MOFs. During the breakthrough process of HOF‐76a, the pure C_2_H_4_ production from the effluent for a given cycle was calculated to be 7.2 L kg^−1^, which outperformed most top‐performing MOFs such as MAF‐49 (6.2 L kg^−1^),^[^ ^139^ ^]^ Cu(Qc)2 (4.4 L kg^−1^),^[^ ^140^ ^]^ PCN‐250 (3.36 L kg^−1^).^[^ ^141^ ^]^ Recently, Chen et al. constructed ZJU‐HOF‐1 with a unique pore structure and (a specific surface area of 1465 m^2^ g^−1^; cavity size of 4.6 Å) using TMBTI as the monomer. The resulting material efficiently and selectively separated C_2_H_4_ and C_2_H_6_ (Figure 21g).^[^ ^81^ ^]^ The single‐component adsorption isotherm showed that the adsorption of C_2_H_6_ was better than that of C_2_H_4_ at 273 or 298 K. When comparing ZJU‐HOF‐1 and HOF‐76a, ZJU‐HOF‐1 showed a steeper adsorption isotherm for C_2_H_6_, indicating that ZJU‐HOF‐1 absorbed more C_2_H_6_ than HOF‐76a. However, when the C_2_H_6_/C_2_H_4_ (50/50, v/v) mixture was separated, the adsorption capacity of ZJU‐HOF‐1 for C_2_H_6_ (88 cm^3^ g^−1^ at 298 K) was 2.3 times that of HOF‐76 (38 cm^3^ g^−1^) (Figure 21h). The unique pore structure of ZJU‐HOF‐1 gave it a high absorption rate for C_2_H_6_ and a high (2.25) C_2_H_6_/C_2_H_4_ selectivity.

**Figure 22:**
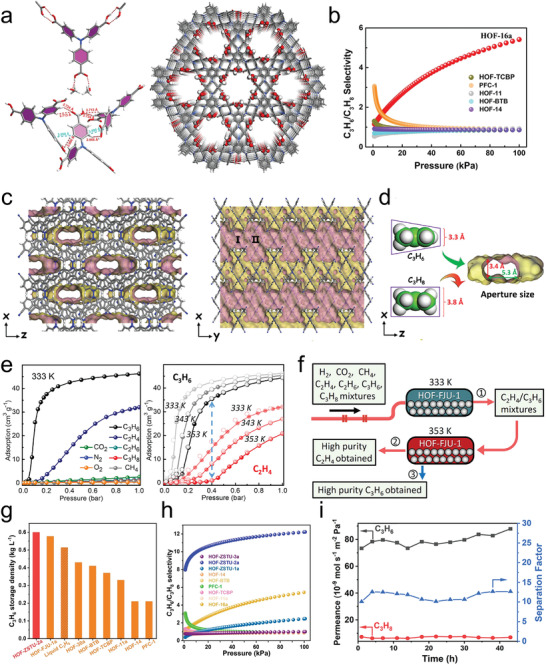
Examples of gas separation. a) Interconnection of different TCAs in HOF‐16 and a view of a 1D pore with exposed carboxylic acid groups in HOF‐16. Reproduced with permission.^[^ ^39^ ^]^ Copyright 2021, Wiley‐VCH. b) The IAST selectivity of HOF‐16a, HOF‐11, and other HOFs at 298 K. Reproduced with permission.^[^ ^39^ ^]^ Copyright 2021, Wiley‐VCH. c) Framework with pore channels along the *y*‐direction, with alternating small necks i) and large cages (II). Reproduced with permission.^[^ ^142^ ^]^ Copyright 2022, American Chemical Society. d) Schematic diagram of the molecular sieving of C_3_H_6_. Reproduced with permission.^[^ ^142^ ^]^ Copyright 2022, American Chemical Society. e) Adsorption isotherms of CH_4_, C_2_H_4_, C_2_H_6_, C_3_H_6_, C_3_H_8_, CO_2_, N_2_, and O_2_ of HOF‐FJU‐1 at 333 K and C_2_H_4_ and C_3_H_6_ sorption isotherms at 333, 343, and 353 K. Reproduced with permission.^[^ ^142^ ^]^ Copyright 2022, American Chemical Society. f) Schematic diagram of the simultaneous separation of C_2_H_4_ and C_3_H_6_ by HOF‐FJU‐1. Reproduced with permission.^[^ ^142^ ^]^ Copyright 2022, American Chemical Society. g) C_3_H_6_ packing densities under ambient conditions. Reproduced with permission.^[^ ^143^ ^]^ Copyright 2023, Wiley‐VCH. h) Selectivity of HOF‐ZSTU‐M compared with carboxylic acid‐based HOFs at 298 K. Reproduced with permission.^[^ ^143^ ^]^ Copyright 2023, Wiley‐VCH. i) Long‐term durability of the HOF‐BTB@AAO membrane for C_3_H_6_/C_3_H_8_ separation at RT and 100 kPa. Reproduced with permission.^[^ ^144^ ^]^ Copyright 2024, Springer Nature.

Propylene (C_3_H_6_) is another important olefin feedstock used to produce chemicals and is mainly derived from the steam cracking of naphtha or propane (C_3_H_8_). Chen et al. constructed a free carboxylic acid‐functionalized HOF (HOF‐16) through crystal engineering and used it for the efficient separation of C_3_H_6_/C_3_H_8_ mixtures.^[^ ^39^ ^]^ With its suitable pores and abundant free ─COOH sites, the absorption capacity of HF‐16A at 298 K (58.8 cm^3^ g^−1^) was significantly higher than that of C_3_H_8_ (33.3 cm^3^ g^−1^). Its IAST selectivity reached 5.4, which was better than that of other HOFs with ─COOH groups (**Figure** **22**a,b). In addition, HOF‐16a was easy to scale up and synthesize and showed excellent water and acid stability.

Considering that the pore size of HOF‐FJU‐1a (3.4 Å × 5.3 Å) was slightly larger than the minimum cross‐sectional area of C_3_H_6_ (3.3 Å× 4.2 Å), but smaller than the minimum cross‐sectional area of C_3_H_8_ (3.8 Å × 4.1 Å), Chen et al. further applied this unique HOF to the more challenging C_3_H_6_/C_3_H_8_ separation (Figure 22c,d).^[^ ^142^ ^]^ At 333 K, the adsorption isotherm showed that HOF‐FJU‐1a completely blocked C_3_H_8_ but adsorbed more C_3_H_6_ (43.6 cm^3^ g^−1^), thus achieving an ultra‐high C_3_H_6_/C_3_H_8_ selectivity of 616 (Figure 22e) and setting a new benchmark for porous adsorbents. This unique HOF efficiently captured C_3_H_6_ from binary C_3_H_6_/C_3_H_8_ mixtures and other gas mixtures at 333 K but also produced high‐purity propylene (99.5%) and ethylene (98.3%) through subsequent column separation (Figure 22f). Recently, Cai et al. obtained a series of HOF‐ZSTU‐M (*M* = 1, 2, and 3) materials with different pore structures by introducing structure‐directing agents (SDAs) into the hydrogen bond network of tetrakis(4‐carboxyphenyl) porphyrin (TCPP).^[^ ^143^ ^]^ HOF‐ZSTU‐2 formed a strong layered hydrogen bond network with ammonium (NH_4_^+^) via multiple carboxyl groups, with a 1D “pearl‐chain” channel that could selectively capture propylene (C_3_H_6_). At 298 K and 1 bar, HOF‐ZSTU‐2 (Figure 22g,h) had a C_3_H_6_ storage capacity of 0.6 kg L^−1^, representing one of the best storage materials reported to date. It also selectively separated propylene/propane (C_3_H_6_/C_3_H_8_). Highly crystalline and continuous HOF films (HOF‐BTB@AAO) have been prepared on large‐pore anodic alumina (AAO) disks by Yin's group and have shown good pore structures and a selective sieving effect. This provided a new strategy for preparing crystalline HOF films with similar functions.^[^ ^144^ ^]^ The mixing and separation capacity of the HOF‐BTB@AAO membrane for an isomeric C_3_H_6_/C_3_H_8_ mixture remained unchanged for 44 h. The permeability of C_3_H_8_ remained low throughout the whole process, thus confirming the excellent molecular sieve separation effect of C_3_H_8_. In general, HOF‐BTB@AAO membranes have demonstrated the robustness, high selectivity, and long‐term stability required to separate C_3_H_6_/C_3_H_8_ (Figure 22i). Compared with MOF‐based membranes or COF‐based membranes, HOF‐based membranes have been studied more intensively for the separation of small‐molecule olefins. Some conventionally prepared membrane materials with pore sizes larger than small gas molecules such as C2 and C3 hydrocarbons cannot be selectively separated via a traditional physical sieving mechanism. For small‐molecule gas separation, HOF‐based membranes have shown advantages or have played an auxiliary role in improving membrane permeability, optimizing separation efficiency, and coping with complex chemical environments.

## 5 Environmental Applications

As novel porous materials, HOFs show great potential for environmental treatment applications due to their high specific surface area, easily adjustable pore size, easy regeneration and recyclability, metal‐free chemical compositions, and adjustable stability. The fluorescence sensing and chiral recognition properties of HOFs can be used to detect heavy metal ions and environmental organic contaminants, providing sensitivity and selectivity superior to those of traditional detection techniques.^[^ ^78^ ^]^

The porosity and high specific surface area of HOFs allow them to separate and adsorb gases, ions, and organic pollutants. HOF‐based materials can be applied to mediate the transformation of environmental substances, especially the removal or degradation of pollutants. For example, HOFs can be used to degrade dyes and reduce and convert CO_2_. Most current HOF‐based materials do not contain metal ions, giving them low density, low cytotoxicity, and good biocompatibility. HOFs are used in bio‐related fields such as enzyme binding/drug delivery/antibacterial applications, to catalyze/treat/inhibit microbial growth in specific environmental areas, or reduce biological or environmental risks.^[^ ^145^ ^]^ Moreover, the applications of HOFs for environmental remediation still need to be explored, such as refining the types of HOFs acting with pollutants, their pathways, and their effects, and expanding their applications for remediating air and water pollution to the remediation of contaminated land. The recent research progress of HOF as a unique research platform for the detection, adsorption, and separation of environmental substances has been systematically reviewed.^[^ ^5^, ^146^ ^]^

### 5.1 Detection of Environmental Substances

#### 5.1.1 Fluorescence Sensing

HOFs with stable permanent pores typically have rigid molecules with aromatic groups as their skeleton, with rigid π‐conjugated molecules acting both as scaffolds for pore structures and as luminescent centers (fluorescence and phosphorescence effects). These types of HOFs show great potential in a variety of optical applications.^[^ ^147^ ^]^

**Figure 23:**
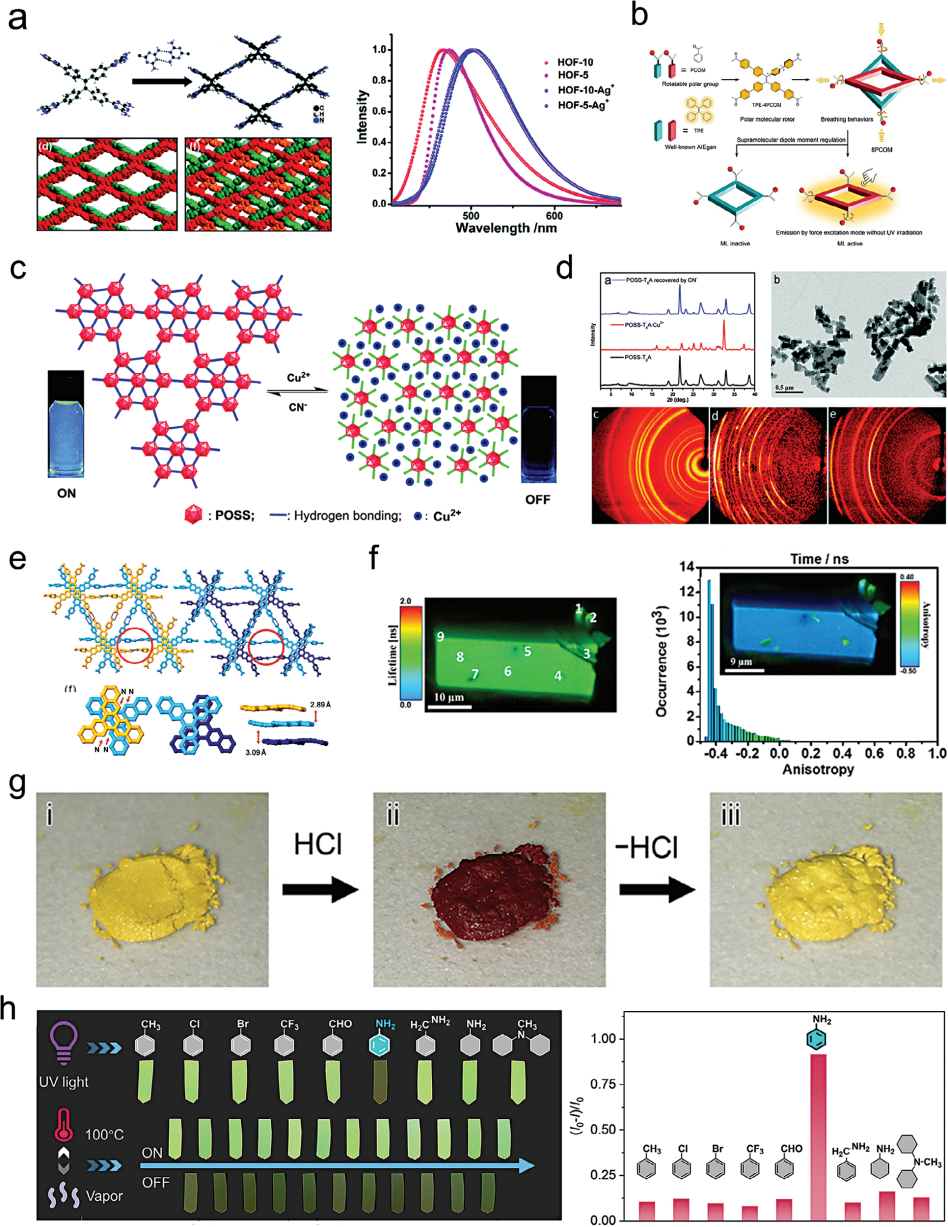
Fluorescence‐sensing properties of HOFs. a) Crystal structures of HOF‐10 and HOF‐5 and room‐temperature solid‐state emission spectra (λ_ex_ = 360 nm) of HOFs and samples after treatment with a THF/water solution of AgNO_3_. Reproduced with permission.^[^ ^84^ ^]^ Copyright 2017, Royal Society of Chemistry. b) Design strategy for the ML‐active flexible 8PCOM framework using a polar molecular rotor as the organic building block. Reproduced with permission.^[^ ^149^ ^]^ Copyright 2021, Chinese Chemical Society. c) POSS‐T8A, POSS‐T8B, and an illustration of the structure of POSS‐T8A used for Cu^2+^ sensing and d) Information about POSS‐T8A, POSS‐T8ACu^2+^, and POSS‐T8A recovered by CNÿ. Reproduced with permission.^[^ ^151^ ^]^ Copyright 2015, Royal Society of Chemistry. e) Crystal structures of the activated form of CPHATN‐1a. Reproduced with permission.^[^ ^54^ ^]^ Copyright 2019, American Chemical Society. f) Fluorescence image of a single CPHATN‐1a crystal. Reproduced with permission.^[^ ^54^ ^]^ Copyright 2019, American Chemical Society. g) HCl‐responsive color changes of bulk crystals of CPHATN‐1a. Reproduced with permission.^[^ ^54^ ^]^ Copyright 2019, American Chemical Society. h) Photographs of spiro‐4N test strips in response to various guest vapors and the recycling process under 365 nm UV illumination. Reproduced with permission.^[^ ^154^ ^]^ Copyright 2023, Wiley‐VCH.

Tetraphenylvinyl (TPE) is a prototype aggregation‐induced emission (AIE) fluorophore that provides excellent solid‐state luminescence and has been widely used as an essential component of luminous HOFs.^[^ ^148^ ^]^ In 2015, Chen et al. reported a flexible microporous HF‐5 for gas adsorption and separation.^[^ ^40^ ^]^ Sun et al. found a polymorph of HOF‐5 and constructed HOF‐1111 capable of fluorescence emission^[^ ^26b^ ^]^ with a peak at 524 nm and fluorescence lifetimes of 4.72 and 2.56 ns. The fluorescence emission of HOF‐1111 was quenched by small‐molecule organic compounds, which shows potential for chemical sensors. In 2017, Chen et al. constructed the polymorphs HF‐10 and HF‐5 which contained a residue that could bind to solvent guest molecules via N─H⋯O interactions.^[^ ^84^ ^]^ These served as binding sites for metal ions during fluorescence sensing. After soaking in 5.0 mM AgNO_3_ solution, HOF‐5 and HOF‐10 showed yellow‐green emission under 360 nm irradiation, with emissions maxima at 502 and 504 nm, respectively. However, for Ca^2+^, Cd^2+^, CO^2+^, Cu^2+^, Mn^2+^, Na^+^, Ni^2+^, and Zn^2+^, almost no emission shift was observed, confirming its high selectivity toward Ag^+^ (**Figure** **23**a).

HOFs with mechanoluminescent (ML) properties are attractive for advanced optical materials. Chi et al. first reported a flexible HOF (8PCOM) with ML and photoluminescence properties (Figure 23b).^[^ ^149^ ^]^ Under UV irradiation, 8PCOM‐DCM in the crystal emitted blue fluorescence with an efficiency of 81% (ΦF). Under ambient conditions, when 8PCOM‐DCM was scraped with a stainless steel spoon, its bright blue color could be observed by the naked eye. The porous organic framework minimized the ACQ effect and showed customizable excited‐state properties, giving it potential applications for tunable laser systems. Zhao et al. used laser‐responsive HOF microcrystals.^[^ ^150^ ^]^ In the microcrystals, HOF‐FJU‐4 and HOF‐FJU‐5 emitted blue light (483 nm; a lifetime of 1.69 ns; quantum yield of 61.7%) and green light (506 nm; a lifetime of 2.32 ns; quantum yield of 45.7%) and displayed color‐adjustable performance.

Host‐guest interactions in the HOFs channels provided the conditions for electronic interactions (such as charge transfer) between the luminophores, and the resulting luminosity changes can be used for sensing.^[^ ^39^ ^]^ Zhou et al. used TPE to construct HOFs (POSS‐T8A) for luminescence/fluorescence sensing by introducing cross–linked groups.^[^ ^151^ ^]^ This material showed AIE emission and strong selective fluorescence quenching for Cu^2+^ (KSV = 30 305 L mol^−1^), and no significant fluorescence response toward other metal ions such as Mn^2+^, Co^2+^, Ni^2+^, and Fe^3+^ (Figure 23c,d). This was attributed to the conformation change of the TPE unit in POSS‐T8A caused by the binding of an amide to Cu^2+^. In addition, the fluorescence of POSS‐T8A was restored by adding cyanide to remove Cu^2+^ without affecting the subsequent Cu^2+^ sensing activity. Recently, Hisaki et al. constructed a 1,2,4‐trichlorobenzene derivative containing carboxybenzene (CPHATN) using π–π stacking to construct an acid‐responsive HOF (CPHATN‐1A) that was used as a sensor to detect acidic atmospheres via a reversible color change (Figure 23e).^[^ ^54^ ^]^ The intramolecular charge transfer and interunit proton transfer reactions occurred in CPHATN‐1a, showing rich photochemical properties (Figure 23f). Its color changed from yellow to reddish‐brown after encountering 37% HCl solution. By heating the reddish‐brown substance, its color gradually recovered as the HCl evaporated (Figure 23g). The reversible color change of CPHATN‐1a was attributed to the protonation/deprotonation of pyridinyl N atoms embedded in its π‐conjugated core. Lin et al. also constructed a hydroxy‐modified mesoporous HOF (HOF‐FAFU‐1) using π–π stacking.^[^ ^51^ ^]^ HOF‐FAFU‐1 was used as a fluorescence sensor to detect hypochlorite in water by fluorescence quenching, and it showed a wide linear range (0–0.45 mmol L^−1^), fast response time (15 s), and low limit of detection (1.32 µmol L^−1^). Recently, an aggregation‐induced emission (AIE)‐active HOF (called HOF‐TPE‐CN) constructed from a cyano‐modified tetraphene linker has been reported for the fluorescence sensing of mononitrophenol isomers.^[^ ^152^ ^]^ Due to AIE, the fluorescence emission performance of HOF‐TPE‐CN was superior, and its quantum yield was 21% higher than that (2.89%) of the TPE‐CN ligand in DMF. Most importantly, the HOF exhibited the highly efficient fluorescence quenching of mononitrophenol isomers in water with a very low detection limit of 0.65 µm. In addition, HOF‐TPE‐CN was more responsive and selective to mononitrophenol than a series of congeners.

The photoelectrochemical properties of HOFs can be used for photoelectrochemical detection. Wang et al. prepared AgNPs@HOF‐101 as a photoactive material for photoelectrochemical sensing, and its photocurrent (0.85–1.91 µA) was significantly higher than that of other common MOFs and metal oxides.^[^ ^153^ ^]^ The sensor selectively identified 13 different simulated mustard gases with an extremely narrow detection range (e.g., 2‐chloroethyl ethyl sulfide with a CEES of 15.8 nmol L^−1^), a reusability of 30 cycles, and a stability of 30 days. Huang et al. reported the first non‐contact, high‐contrast sensing of aromatic amines using a HOF based on nitro‐modified stereostructural units.^[^ ^154^ ^]^ The non‐contact sensing of aniline and diphenylamine was realized, with quenching efficiencies of up to 91.7% and 97.0% (Figure 23h).

To date, the development of various types of organic framework materials (HOFs, MOFs, and COFs) for fluorescence sensing still faces great challenges. It is difficult to accurately control the pore and structure of MOFs, which directly affects their sensing performance and poor stability in water and adverse environments.^[^ ^155^ ^]^ Although HOFs and COFs have recently been used in fluorescence sensing systems, there are still challenges that need to be systematically investigated, such as unclear mechanisms, limited variety of materials, and solution processability and suitability.^[^ ^147^, ^156^ ^]^ For example, the introduction of metal nanoparticles into the pores of COFs or HOFs may broaden their applications, but it may also affect their phase purity. Therefore, the modification of functional groups and the related fluorescence sensing mechanisms should be thoroughly investigated. The most important optimization direction is to improve the conductivity of HOF transmitters to facilitate electron transfer during reactions. Although fluorescence sensing based on HOFs is still in the laboratory stage, with a large number of studies, it is believed that HOFs will open up another valuable path for future research and applications.

#### 5.1.2 Chiral Recognition

Molecular recognition plays an important role in biological systems involving specific intermolecular interactions via non‐covalent bonding such as H bonding, π interactions, and van der Waals forces. HOFs play a key role in biomolecule recognition, due to their characteristics: 1) functional sites with specific recognition ability; 2) flexible and adaptable structures; 3) high crystallinity, which allows host‐guest interactions.^[^ ^5a^ ^]^ Chiral centers provide asymmetric pores for selective separation, while additional H‐bond donors/acceptors provide binding sites for complementary analytes.

**Figure 24:**
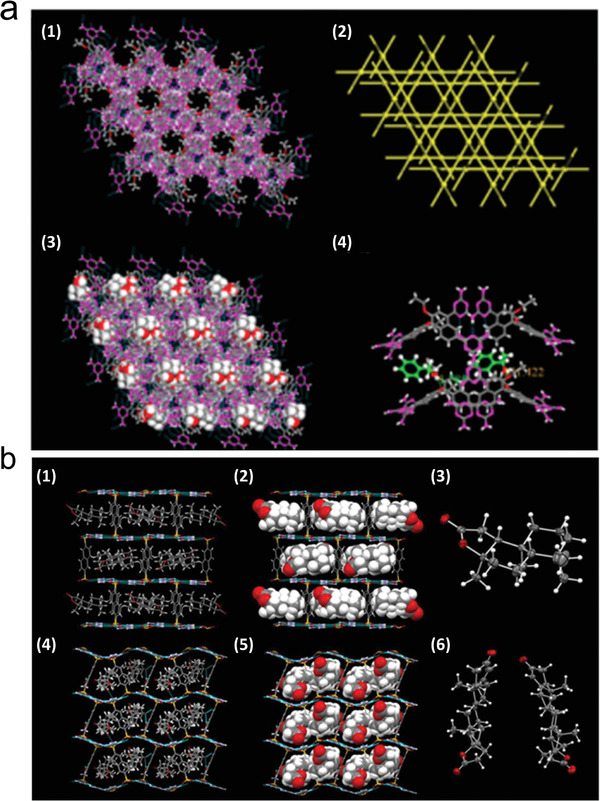
Chiral recognition properties of HOFs. a) X‐ray crystal structure of HOF‐2 featuring: 1) 3D hydrogen‐bonded organic framework with 1D channels and 2) simplified as a 6‐connected network. 3) X‐ray crystal structure of HOF‐2⊃R‐1‐PEA and 4) highlight of the chiral cavities for the specific recognition of R‐1‐PEA. Reproduced with permission.^[^ ^43^ ^]^ Copyright 2014, American Chemical Society. b) Illustrative crystal structures of GS inclusion compounds. (1–3) (G2NDS)⊃(3aR)‐(+)‐sclareolide (5); (4–6) (G2NDS) ⊃ (drospirenone)(methanol)_0.84_(H2O)_0.1_(6). Reproduced with permission.^[^ ^159^ ^]^ Copyright 2019, Springer Nature.

Enantioselective separation of chiral objects can be achieved by introducing chiral centers into an HOF. In 2014, Chen et al. showed that 1,1′‐bi‐2‐naphthol (BINOL) is a very powerful organic backbone for asymmetric induction, and they incorporated the (R)‐BINOL scaffold into a 2,4‐diaminotriazinyl hydrogen‐bonding motif to synthesize a new chiral organic building block used to self‐assemble a HOF.^[^ ^43^ ^]^ The constructed chiral HOF‐2 showed a 3D open framework and 1D pores (size 4.8 Å) and remained stable due to multiple hydrogen bonds and π–π interactions. It showed a specific surface area of 237.6 m^2^ g^−1^, and its chiral center was exposed to the pore surface, which facilitated enantioselective recognition of small molecules (**Figure** **24**a). Introducing functional sites into porous materials is an effective method for the selective identification of small molecules. Due to its chiral pore, HOF‐2 has been used for the adsorption and resolution of various secondary alcohols, such as phenyl ethanol (up to 92% enantiomeric excess). Recently, Chen et al. reported that HOF‐9 has a higher recognition selectivity for pyridine (Py) than BTX aromatic compounds (benzene, toluene, and *o*, *m*, and *p*‐xylene).^[^ ^44^ ^]^ The inner surface of the HF‐9 channel (6.9 Å × 8.8 Å) has a free amino group, which is key for selective recognition of Py molecules. The single crystal structure analysis of HOF‐9‐2Py showed the presence of strong N─H⋯N (3.20 Å^2^) interactions between the unbonded amino group and Py molecule, which confirmed the recognition of Py by HOF‐9. The flexibility of the HOF allowed it to undergo adaptive framework transitions in different guest inclusion processes, thereby preserving its monocrystalline properties. The corresponding host‐guest interactions were directly observed through single‐crystal XRD analysis.

Ward et al. reported guest resolution for different pore channels based on single‐crystal XRD analysis of various guest inclusion compounds of 1, 2, 4, 5‐tetrad(4‐sulfonic phenyl)benzene (G4TSPB) guanyl sulfonate HOF.^[^ ^157^ ^]^ Crystal characterization is challenging for the structural determination of organic molecules with chiral structures. The solution is to adsorb molecules into their pores or co‐crystallize them with molecules that are easy to crystallize, and then test the spatial configuration by single‐crystal XRD.^[^ ^158^ ^]^ Ward et al. encapsulated small molecules in GS‐HOFs by a one‐step crystallization method and then analyzed their molecular structure (including, (3aR)‐(+)‐sclareolide, drospirenone, progesterone) (Figure 24b).^[^ ^159^ ^]^ The structural diversity and flexibility of GS‐HOFs enable the encapsulation of guest molecules of various sizes and shapes and reduce structural disorder and solvent effects. Additionally, the substantial atomic weight of S plays a crucial role in determining the single‐crystal structure. Therefore, it is anticipated that GS‐HOFs can be employed for determining the structures of organic molecules that are challenging to crystallize or possess complex structures.

### 5.2 Adsorption/Separation of Environmental Substances

#### 5.2.1 Adsorption/Separation of Gases

**Figure 25:**
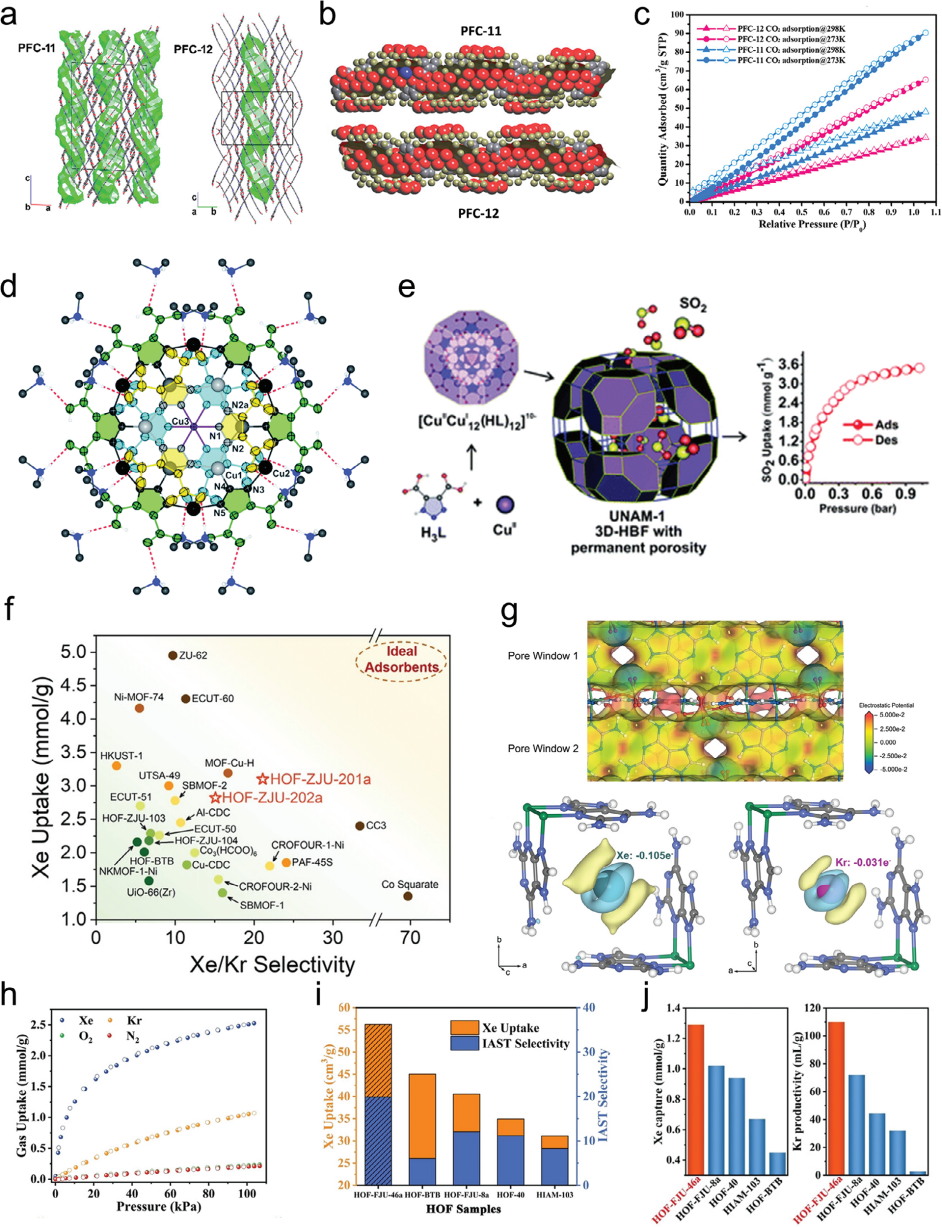
Gas adsorption/separation properties of HOFs. a,b) Composition of channels in PFC‐11 and PFC‐12. Reproduced with permission.^[^ ^24b^ ^]^ Copyright 2020, American Chemical Society. c) CO_2_ adsorption isotherm of PFC‐11 and PFC‐12. Reproduced with permission.^[^ ^24b^ ^]^ Copyright 2020, American Chemical Society. d) Molecular structure of the [Cu_13_(HL)_12_]^10−^ anion (building block of the UNAM‐1 framework) and e) mechanism of action and adsorption properties of SO_2_. Reproduced with permission.^[^ ^161^ ^]^ Copyright 2019, Royal Society of Chemistry. f) Comparison of the Xe uptake and Xe/Kr (20/80, *v/v*) IAST selectivity of various materials at 298 K. Reproduced with permission.^[^ ^163^ ^]^ Copyright 2022, Wiley‐VCH. g) The surface electrostatic potential of HOF‐ZJU‐201 mapped onto the 0.02 Hartree/e density isosurface with a scale spanning −0.05 Hartree/e (blue) through 0 to 0.05 Hartree/e (red). Reproduced with permission.^[^ ^163^ ^]^ Copyright 2022, Wiley‐VCH. h) Single‐component Xe, Kr, O_2_, and N_2_ adsorption isotherms of HOF‐FJU‐46a at 296 K. Reproduced with permission.^[^ ^164^ ^]^ 2023 Wiley‐VCH. i) Comparison of Xe uptake and IAST selectivity of HOF‐FJU‐46a with other reported HOF materials. Reproduced with permission.^[^ ^164^ ^]^ 2023 Wiley‐VCH. j) Xe capture and high‐purity Kr productivity. Reproduced with permission.^[^ ^164^ ^]^ 2023 Wiley‐VCH.

The pore shape, size, surface area, and active sites of HOFs are adjustable, giving HOFs large mass adsorption capacities, which allows them to adsorb and separate gases.^[^ ^7^, ^29^ ^]^ The development of CO_2_ adsorbents with a high adsorption capacity and selectivity is the key to mitigating the greenhouse effect. Interactions between HOFs and CO_2_ are stronger than those between nitrogen, giving HOFs excellent CO_2_/N_2_ selective adsorption properties. In 2020, Li et al. synthesized PFC‐11 and PFC‐12 with hexagonal honeycomb structures to efficiently adsorb CO_2_ (**Figure** **25**a,b).^[^ ^24b^ ^]^ At a pressure of 1 bar, the CO_2_ absorption capacities of PFC‐11 and PFC‐12 were 90.3 and 65.1 cm^3^ g^−1^, respectively, which were higher than those of other HOFs (Figure 25c).

Sulfur dioxide (SO_2_) is a pollutant that can poison some catalysts, but it cannot be removed using commonly used adsorption methods.^[^ ^160^ ^]^ UNAM‐1, reported by Jancik et al. showed a very high adsorption selectivity for SO_2_ and it exhibited reversible SO_2_ adsorption properties.^[^ ^161^ ^]^ At a pressure of 0.05 bar, the adsorption capacity of SO_2_ was 1.1 mmol g^−1^, which increased with pressure (1 bar, 3.5 mmol g^−1^) (Figure 25d,e).

Xenon (Xe) and Krypton (Kr) are commonly utilized in applications such as lighting, lasers, and medical equipment. Therefore, it is crucial to separate them through adsorption. Bao's research team achieved this separation using two ionic HOFs, namely HOF‐ZJU‐103 and HOF‐ZJU‐104 189.^[^ ^162^ ^]^ The abundant polar anions and amino groups strongly adsorbed Xe, and the Xe capacity and Xe/Kr selectivity of HOF‐ZJU‐103 at 298 K and 1 bar were 2.29 and 6.9 mmol g^−1^, respectively. Bao et al. built two isostructural hydrogen‐bonded metal‐nucleobase frameworks (HOF‐ZJU‐201 and HOF‐ZJU‐202) that could also separate Xe/Kr under ambient conditions to strike a balance between capacity and selectivity.^[^ ^163^ ^]^ The Xe adsorption capacity of HOF‐ZJU‐201a reached 3.01 mmol g^−1^ at 298 K and 1.0 bar, while the IAST selectivity and Henry's selectivity were 21.0 and 21.6, respectively (Figure 25f). Direct breakthrough experiments showed an Xe capacity of 25.8 mmol kg^−1^ from a dilute Xe/Kr gas mixture. DFT calculations revealed that the selective binding arose from the enhanced polarization in the confined electric field produced by the electron‐rich anions and the electron‐deficient purine heterocyclic rings (Figure 25g). Recently, Gong et al. reported that HOF‐40 could also separate Xe/Kr gas mixtures.^[^ ^88^ ^]^ Huang et al. report the first hydrogen‐bonded tetramer‐based microporous HOF (HOF‐FJU‐46), self‐assembled from tetrakis(4‐(1H‐pyrazol‐4‐yl)phenyl) silane.^[^ ^164^ ^]^ The experimental results confirmed that activated HOF‐FJU‐46 had a xenon (Xe) absorption rate of 2.51 mmol g^−1^ under ambient environmental conditions (Figure 25h) and a Xe/Kr selectivity of 19.9, which was the highest among HOFs reported so far. Dynamic tests confirmed that HOF‐FJU‐46a had excellent Kr productivity (110 mL g^−1^) and Xe absorption rate (1.29 mmol g^−1^) (Figure 25i,g), as well as good recoverability. Yuan et al. devised a method to optimize pore engineering by substituting the benzene ring in the core of HOF‐40 with a larger dipyrrole ring to prepare a larger HOF‐FJU‐8a with a pore diameter of 4.2 Å × 4.6 Å.^[^ ^165^ ^]^ HOF‐FJU‐8a showed the highest Xe/Kr separation performance reported so far, reaching 8.5, and the Kr productivity exceeding 72 L kg^−1^. The custom aperture of the HOF‐FJU‐8a played a key role in achieving significantly different interactions and binding affinities between host and guest.

#### 5.2.2 Adsorption/Separation of Ions

**Figure 26:**
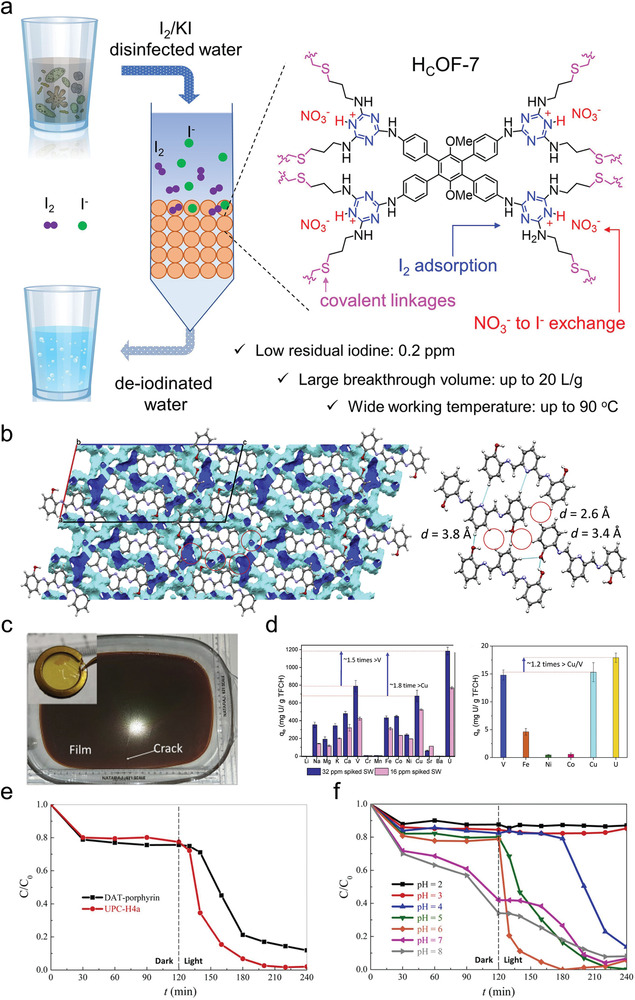
a) Schematic representation of the synergistic removal of I_2_/I^−^ from water using ionic HCOF‐7. Reproduced with permission.^[^ ^168^ ^]^ Copyright 2022, Wiley‐VCH. b) ^CSMCRI^HOF‐1 molecular arrangement showing the continuous channels along the *b*‐axis and the pore channel openings (*d* = diameter). Reproduced with permission.^[^ ^171^ ^]^ Copyright 2022, Elsevier. c) Scalable preparation of a large‐area free‐standing thin film of ^CSMCRI^HOF‐1. Reproduced with permission.^[^ ^171^ ^]^ Copyright 2022, Elsevier. d) Selectivity profile of 16 ppm in simulated seawater and 32 ppm in natural seawater. Reproduced with permission.^[^ ^171^ ^]^ Copyright 2022, Elsevier. e) Photocatalytic removal of U(VI) by DAT‐porphyrin and UPC‐H4a. Reproduced with permission.^[^ ^173^ ^]^ Copyright 2023, Elsevier. f) Effect of pH on the photocatalytic removal of U(VI) by UPC‐H4a. Reproduced with permission.^[^ ^173^ ^]^ Copyright 2023, Elsevier.

HOFs can also selectively adsorb ions due to their suitable pore size and pore structure regulation. Iodine is a broad‐spectrum antibacterial disinfection agent, but low residual concentrations of iodine and iodide need to be removed over a wide temperature range using high‐affinity porous adsorbents.^[^ ^166^ ^]^ While most porous materials show substantial gas‐phase iodine adsorption only at high iodine concentrations, progress in iodine removal at low concentrations has been limited.^[^ ^167^ ^]^ Ke et al. reported HCOF‐7 that could adsorb iodine via halogen bonding interactions and iodide via anion exchange, achieving the best‐known iodine adsorption performance (**Figure** **26**a).^[^ ^168^ ^]^ When the concentration of I_2_/I^−^ was 5 ppm, HCOF‐7 reduced the residual concentration of I_2_/I^−^ to 0.22 ppm at 23 °C and 0.45 ppm at 90 °C, indicating a high removal rate of iodine/iodide over a wide temperature range. Recently, Li et al. synthesized a variety of HOFs with one‐, two‐, or 3D hydrogen‐bond frameworks using benzene‐1,3,5‐tricarboxamide and amide‐containing tribenzocyclynes as the basic building blocks.^[^ ^169^ ^]^ Among them, HOF_B‐Hex, HOF_T‐Pr, and HOF_T‐Hex showed permanent porosity, with HOF_T‐Hex showing a porosity of 42% and an iodine capture efficiency of 6.4 g g^−1^.

Heavy metal ions generally do not biodegrade and can accumulate via the food chain.^[^ ^170^ ^]^ The ordered pores of HOFs can accelerate mass transfer to adsorb heavy metal ions. In 2022, Kaushik et al. reported a single‐component HOF (^CSMCRI^HOF‐1) adsorbent that achieved the most efficient extraction of uranium from seawater.^[^ ^171^ ^] CSMCRI^HOF‐1 showed excellent stability (stable in water, acid, alkali, salt, and high ionic strength media), thermal stability (maximum tolerated temperature of 340 °C), and a fixed pore structure (pore size of 3.6–3.8 Å, BET surface area of 328 m^2^ g^−1^) (Figure 26b). The researchers demonstrated for the first time that porous ^CSMCRI^HOF‐1 could be treated in solution to form a large‐area crystalline self‐supporting film with an adjustable thickness (Figure 26c). This film showed excellent adsorption properties (17.80 mg U g^−1^) and a long service life, demonstrating the potential of HOFs for metal ion adsorption (Figure 26d). Recently, a ^CSMCRI^HOF2‐P thin film constructed by Maurya et al. extracted ≈14.8 mg g^−1^ in 4 weeks from natural seawater, with a uranium‐to‐vanadium selectivity ratio (U/V) of > 1.7 U/V.^[^ ^172^ ^]^ Wu et al. synthesized HOFs via the intermolecular self‐assembly of 5,10,15,20‐tetra(4‐(2,4‐diaminotriazine)phenyl) porphyrin to remove U(VI) from aqueous solutions.^[^ ^173^ ^]^ The synthesized UPC‐H4a had a high crystallinity and permanent porosity and removed 98.18% of U(VI) after illumination for 120 min (Figure 26e). UPC‐H4a was used to treat actual low‐radioactivity wastewater, and its removal rate reached 66.14% in the presence of competing redox‐active metal ions (Figure 26f). Chen et al. constructed a fluorescence sensor from an HOF using a pore engineering approach to realize the ultra‐sensitive and selective detection of free Cu^2+^.^[^ ^174^ ^]^ Fluorescence quenching enabled this novel HOF sensor to quantify Cu^2+^ in human serum matrix with a linearity of 50–20 000 nm, a detection limit of 10 nm, and a recovery rate of 89.5–115%, which was superior to the performance of other similar materials.

#### 5.2.3 Screening and Removal of Organic Matter

Due to the wide variety of organic pollutants, HOFs can be modified to contain high‐affinity active sites to improve their adsorption capabilities.^[^ ^78^ ^]^ The HOFs adsorption process mainly depends on hydrogen bonding, electrostatic interactions, π–π interactions, and Lewis acid‐base interactions.^[^ ^175^ ^]^

*p*‐Xylene is a commonly used chemical, and its improper disposal will also cause harm to the environment and body. FDM‐15 was constructed by Li et al. to selectively adsorb *p*‐xylene.^[^ ^38^ ^]^ In a mixed solution of benzene‐type molecules, only *p*‐xylene was adsorbed into the channels of FDM‐15. The primary reason for this selective adsorption was not the volume exclusion effect, but rather weak interactions between the framework and *p*‐xylene. FDM‐15 selectively adsorbed *p*‐xylene/*o*‐xylene with a separation coefficient of 1.87:1 and *p*‐xylene/ethylbenzene with a separation coefficient of 1.41: 1.

**Figure 27:**
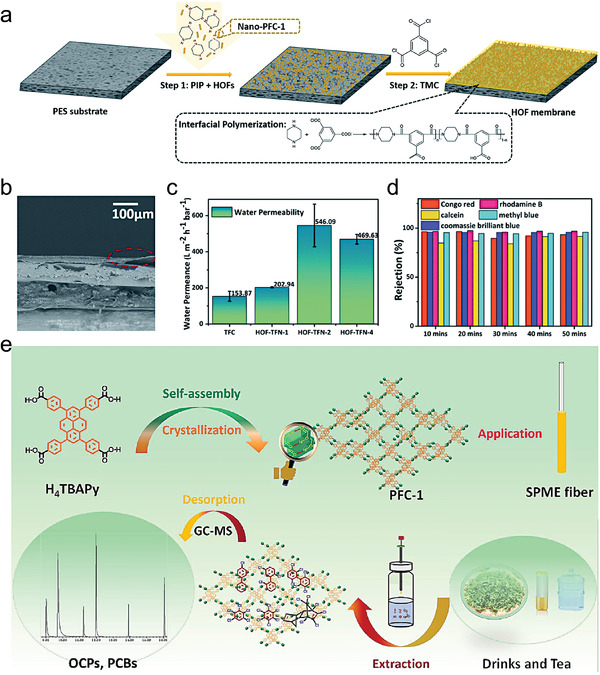
Screening and removal of organic matter by HOFs. a) Preparation process of a HOF‐TFN membrane containing PFC‐1 nanoparticles via interfacial polymerization; b) SEM images of the cross‐section of the HOF‐TNF‐2 substrate; c) Water permeability of HOF‐TNF‐2; d) Dye separation performance of the HOF‐TFN‐2 membrane. Reproduced with permission.^[^ ^177^ ^]^ Copyright 2021, Royal Society of Chemistry. e) Illustration of the fabrication of PFC‐1 fiber and its application for analyzing OCPs and PCBs when coupled with GC‐MS. Reproduced with permission.^[^ ^181^ ^]^ Copyright 2023, Elsevier.

Yang et al. reported that PFC‐1 assembled by hydrogen bonding and π–π interactions strongly adsorbed Rhodamine B (RhB) and methyl orange (MO), with a maximum adsorption capacity of 317 and 252 mg g^−1^ at 298.15 K, respectively.^[^ ^82^ ^]^ These values were better than those of commercially available activated carbon and some representative MOFs and COFs.^[^ ^176^ ^]^ Electrostatic attraction, hydrogen bonds, and π–π interactions are the main reasons for the adsorption of RhB by PFC‐1, while van der Waals forces played an important role in the adsorption of MO. PFC‐1 showed high stability and structural integrity during adsorption. Liu et al. prepared a thin‐film nanocomposite (TFN) permeable film containing porous HOF (PFC‐1) nanoparticles by interfacial polymerization (**Figure** **27**a).^[^ ^177^ ^]^ The membrane showed a highly ordered porous structure and direct molecular separation channels. Due to the ultra‐thin properties of TFN films and the highly‐ordered porous structure of PFC‐1 nanoparticles (Figure 27b), these flexible HOF films showed both an ultra‐high water permeability (546.09 L m^−2^ h^−1^ bar^−1^) (Figure 27c) and excellent dye molecular barrier properties (Figure 27d). The retention rate of RhB was >97%. They also demonstrated long‐term operational stability and excellent cycling performance, providing a reference for the development of HOF membranes for liquid‐phase molecular separation. Recently, Zhang et al. prepared a complex (TiO_2_@HOF) of titanium dioxide (TiO_2_) and a hydrogen‐bonded organic skeleton (HOF‐TCPB‐373).^[^ ^178^ ^]^ Compared with HOF‐TCPB‐373, 40% TiO_2_@HOF showed a greater BET (26.68 m^2^ g^−1^). Compared with TiO_2_, 40% TiO_2_@HOF was more hydrophilic, with a water contact angle of 51°. Briefly, 40% TiO_2_@HOF combined photodegradation (69%) and adsorption (30%), removing up to 99% of RhB.

In 2017, Yao et al. prepared HOIF‐1, which contained lanthanide (pore size of 0.75 nm), and prepared alumina‐supported HOIF‐1 film via immobilization.^[^ ^179^ ^]^ The film selectively filtered dyes larger than 0.75 nm × 0.75 nm, such as RhB, trypan blue (TB), and reactive blue 19 (RB 19). Lv et al. synthesized negatively‐charged MP‐HOF with abundant oxygen‐containing groups, a π–π conjugated structure, large specific surface area, and high stability, which enhanced its adsorption efficiency for paraquat (PQ) and chlormequat (CQ).^[^ ^180^ ^]^ The method has been applied to the adsorption of paraquat in tap water and river water, thus demonstrating its potential practical applications. In conclusion, HOFs show great potential as efficient green adsorbents for organic dyes. PFC‐1 (self‐assembled by 1,3,6,8‐tetra (4‐carboxylphenyl)pyrene) fiber has shown an excellent adsorption capacity for nitroaromatic compounds (NACs) and persistent organic pollutants.^[^ ^181^ ^]^ PFC‐1 fibers were combined with gas chromatography‐mass spectrometry (GC‐MS) (Figure 27e) to develop an ultra‐sensitive practical analytical method. For organochlorine pesticides (OCP) (0.070–0.082 ng‐L^−1^) and polychlorinated biphenyls (PCB) (0.030–0.084 ng‐L^−1^), it showed a low detection range, good repeatability (6.7–9.9%), and satisfactory reproducibility (4.1–8.2%). MOFs are linked by organic ligands and inorganic metal ions, leading to a lower stability compared with typical adsorbents such as activated carbon, zeolites, or silica. The presence of a large number of reversible bonds in COFs and their chemical stability limits their further development and applications. HOFs are also relatively unstable due to the weak flexibility of hydrogen bonding, making it difficult to maintain a permanent porosity. Therefore, the development of stabilized materials is crucial for adsorption applications, as they can maintain their performance under various environmental conditions, thus ensuring the efficiency and reliability of the adsorption process. Therefore, future HOFs‐based materials should improve both stability and performance to be suitable for practical applications.

### 5.3 Degradation and Transformation of Environmental Substances

#### 5.3.1 Degradation of Pollutants

**Figure 28:**
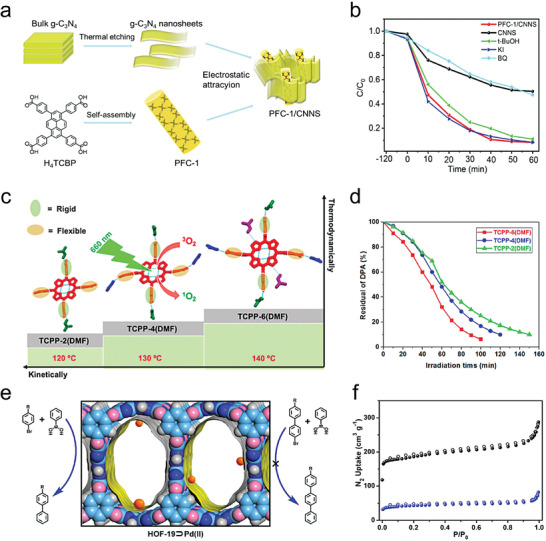
Degradation and transformation of pollutants. a) The synthesis process of CNNS, PFC‐1, and PFC‐1/CNNS heterojunction. Reproduced with permission.^[^ ^182a^ ^]^ Copyright 2022, Elsevier. b) MO degradation by PFC‐1/CNS. Reproduced with permission.^[^ ^182a^ ^]^ Copyright 2022, Elsevier. c) Different HOF products obtained at different temperatures. Reproduced with permission.^[^ ^116c^ ^]^ Copyright 2019, American Chemical Society. d) Plots of the DPA residual concentration versus irradiation time in the presence of three HOFs based on the absorbance peak at 371 nm. Reproduced with permission.^[^ ^116c^ ^]^ Copyright 2019, American Chemical Society. e) Packing diagram of HOF‐19 showing the 1D channel surfaces highlighted as yellow/gray (inner/outer) curved planes. Reproduced with permission.^[^ ^79a^ ^]^ Copyright 2019, American Chemical Society. f) N_2_ sorption isotherms of HOF‐19a (black) and HOF‐19⊃Pd(II) (blue) at 77 K (solid symbols = adsorption; open symbols = desorption). Reproduced with permission.^[^ ^79a^ ^]^ Copyright 2019, American Chemical Society.

HOFs can potentially be used to degrade common pollutants found in contaminated wastewater to reduce environmental pollution.^[^ ^116^, ^182^ ^]^ Shi et al. synthesized a new type of Z PFC‐1/CNNS heterojunction photocatalyst by an in situ electrostatic method.^[^ ^182a^ ^]^ The photocatalyst reduced the recombination rate of holes and electrons and produced highly active substances to photocatalytically degrade MO (**Figure** **28**a).^[^ ^182a^ ^]^ The strong surface hydrophilicity of PFC‐1/CNNS weakened the interactions between water molecules and MO, thus significantly improving the catalytic performance. After 30 min of light exposure, PFC‐1/CNS degraded 80% of MO and completely degraded MO after 60 min (Figure 28b). He et al. investigated the photocatalytic activity of HOFs during the degradation of 9, 10‐diphenyl anthracene (DPA) based on meso‐tetrakis(carboxyphenyl)porphyrin (TCPP).^[^ ^116c^ ^]^ TCPP‐2 (DMF), TCPP‐4 (DMF), and TCPP‐6 (DMF) (Figure 28c) were obtained by blocking the “reverse strategy” of the strong hydrogen bond building unit on the main chain of TCPP and controlling the synthesis of the HOF derived from TCPP. The results showed that TCPP‐6 (DMF) degraded 99% of DPA (Figure 28d) after 100 min of illumination, while TCPP‐2 (DMF) and TCPP‐4 (DMF) required 120 and 150 min, respectively. More importantly, TCPP‐6 (DMF) showed excellent reusability as a photocatalyst, showing that as more DMF molecules were bound, the van der Waals forces around TCPP molecules increased. This increased the photostability and photocatalytic activity of TCPP‐based HOF.

#### 5.3.2 Transformation of Pollutants

The research of HOF‐based catalysts for the transformation of environmental pollutants has gradually become a hot topic.^[^ ^183^ ^]^ The general strategies for constructing HOF‐based catalysts are: 1) encapsulating a catalyst in the HOF pores; 2) using the catalytic unit as a building block for the HOF; (3) modifying the pore surface to expose more active sites on the pore walls. Using the advantages of HOFs to design and prepare new catalysts with high efficiency and easy recovery will provide a new idea for the transformation of pollutants.

In 2019, Han et al. prepared HOF‐19 (Figure 28e) using triazine organic molecular cages as the building units.^[^ ^79a^ ^]^ HOF‐19 contained 1D open and permanent pores with dimensions of 0.80 nm × 1.36 nm and a specific surface area of 685 m^2^ g^−1^. The N_2_ sorption measurement at 77 K reveals its type I adsorption curve with an N_2_ uptake of 287 cm^3^ g^−1^ at 1.0 bar (Figure 28f). Supported Pd(II)@HOF‐19 was prepared by a post‐synthesis method and showed better catalytic activity than palladium acetate or commercially available palladium carbon during Suzuki‐Miyaura coupling. When the amount of catalyst was 0.260 mmol, the recovery of the Suzuki‐Miyaura coupling reaction catalyzed by Pd(II)@HOF‐19 was 96–98%. Pd(II)@HOF‐19 could be regenerated and reused, and the catalytic activity remained unchanged after recrystallization. The catalytic activity of deactivated species could be recovered from an isolated yield of 46–92% for 4‐bromobenzonitrile conversion under the same conditions, which demonstrates the application potential of HOF‐based catalysts.

### 5.4 Environmental Biology Applications

#### 5.4.1 Combination with Enzymes

HOFs can be employed to fabricate composites with tailored pore shapes, sizes, and functions. Recently, they have found applications in enzyme immobilization for environmental catalytic applications.^[^ ^145^ ^]^ The main advantages include biocompatibility, recyclability, and reusability. HOFs can also be used as a hydrogen bond‐biological interface between enzymes and material molecules to maintain the structural stability and activity of enzymes.

**Figure 29:**
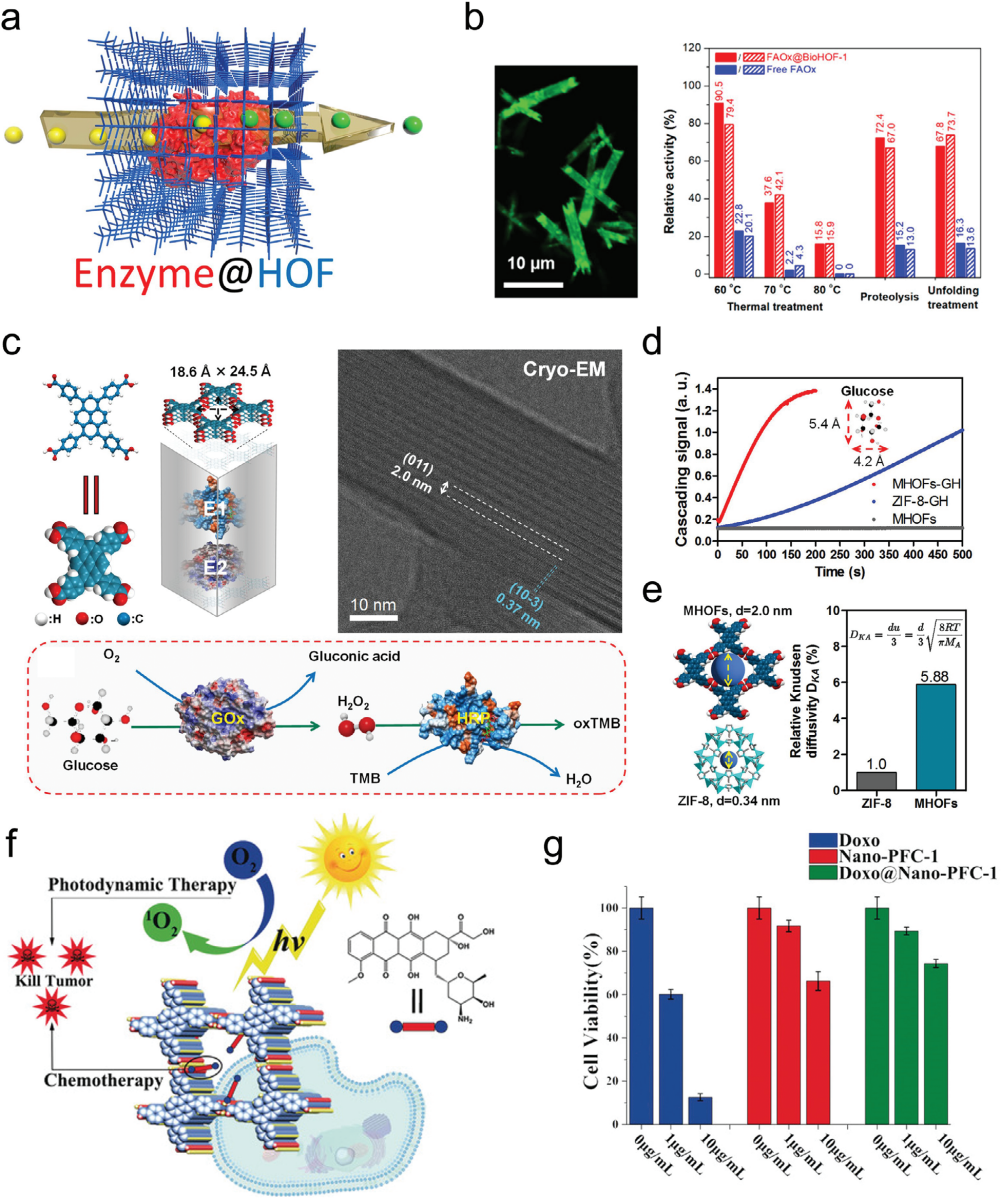
Environmental biology‐related applications of HOFs. a) A schematic representation of the synthesis of enzyme@BioHOF‐1 composites. Reproduced with permission.^[^ ^27c^ ^]^ Copyright 2019, American Chemical Society. b) CLSM image and relative activity (%) of FAOx@BioHOF‐1. Reproduced with permission.^[^ ^27c^ ^]^ Copyright 2019, American Chemical Society. c) Structural illustration of an MHOF cascade nanoreactor and Cryo‐EM structure of an MHOF cascade nanoreactor. Reproduced with permission.^[^ ^185^ ^]^ Copyright 2021, Wiley‐VCH. d) The chemical transformation of GOx‐HRP bi‐enzyme cascade. Reproduced with permission.^[^ ^185^ ^]^ Copyright 2021, Wiley‐VCH. e) The cascade activities of MHOFs‐GH and ZIF‐8‐GH (10 mM glucose). Reproduced with permission.^[^ ^185^ ^]^ Copyright 2021, Wiley‐VCH. f) Mechanism of action of PFC‐1. Reproduced with permission.^[^ ^52^ ^]^ Copyright 2018, Wiley‐VCH. g) In vitro cytotoxicity of Doxo (blue), Nano‐PFC‐1 (red), and Doxo@Nano‐PFC‐1 (green) at various concentrations in HeLa cells. Reproduced with permission.^[^ ^52^ ^]^ Copyright 2018, Wiley‐VCH.

Liang et al. reported the protective effect of an HOF coating on two enzymes and synthesized a water‐stable BioHOF‐1 based on amidine/carboxylate, which consisted of tetramidine and tetra‐carboxylate building blocks.^[^ ^27c^ ^]^ This HOF (with a pore size of ≈0.64 nm) was used to encapsulate CAT (9.7 nm × 9.2 nm × 6.7 nm) to obtain an Enzym@bioHOF‐1 composite (**Figure** **29**a). The composite was stable over a wide pH range of 5–10 and in polar organic solvents and phosphate buffer. To further investigate the effect of this encapsulation strategy, alcohol oxidase (AOx) was encapsulated within BioHOF‐1, and its enzymatic activity was determined. The results showed that the obtained fluorescein‐labeled AOx@BioHOF‐1 maintained 85% of its original activity after being heated to 60 °C (Figure 29b). This was higher than the activity of free enzymes under the same conditions (20%), showing that HOFs can be used to ensure an enzyme's activity and stability. Li et al. reported the first nano‐scale mesoporous HOFs (nmHOFs) for the in situ fixation of enzymes.^[^ ^184^ ^]^ The researchers assembled tetrakis(4‐amidiniumphenyl) methane (TAM) and 1,3,6,8‐tetrakis(*p*‐benzoic acid) pyrene (H4TBAPy) in aqueous solution with enzyme induction into enzyme‐nMHOF (named enzyme@TaTb). Experimental studies showed that lactate dehydrogenase (LDH) could convert pyruvate into lactic acid in the presence of NADH. Compared with LDH@ZIF‐8 and LDH@Bio‐HOF‐1, LDH@TaTb accelerated the diffusion rate of NADH and pyruvate, thus exhibiting an ultra‐high activity close to that of free LDH. TaTb nmHOF could also be used to fix alpha‐amylase and horseradish peroxidase. This research may help develop HOFs as platform vectors for fixing enzymes.

Ouyang et al. developed a biocatalytic cascade nanoplatform (Figure 29c–e) using mesoporous HOFs (MHOFs) as the scaffold.^[^ ^185^ ^]^ They used 1, 3, 6, 8‐tetra(*p*‐benzoate) pyrene (H4TBAPy), a planar molecule with a large π‐conjugated system and four symmetric carboxylic acid groups, to organize two or three enzymes within an ordered mesoporous framework. The ultra‐stable framework and mesoporous transport channels of MHOFs promoted the efficient diffusion of substrates between active sites and accelerated the cascade reaction. The MHOFs cascade biosensor showed promising development potential in clinical diagnosis and required only 1 min to diagnose diabetes. The diagnostic results were completely consistent with the detection values of standard blood glucose measurements. Additionally, Ouyang et al. constructed a novel external HOF to regulate the conformation of cytochrome c, thereby achieving the enzyme's non‐native bioactivity. This robust HOF contained neatly arranged carboxylic acid internal cavities that could be directly assembled onto natural cytochrome c.^[^ ^186^ ^]^ Based on this, Ouyang et al. proposed an in situ encapsulation method to preserve the hydrogen bond biological interface between the enzyme and structure of HOF‐101.^[^ ^187^ ^]^ In this way, the HOF grew in situ around the enzyme's surface. The method was faster (30 min) than the in situ encapsulation of ZIF‐8 (several hours to 1 day). It also showed a high enzyme loading (24.3–47.9% wt.), wide range (stable at pH 2–11), and recyclable (in the case of cytochrome c@HOF, after 10 cycles, the conversion rate of biological activity still remained above 90%). The bioactivity of this enzyme @HOF biocomposite was superior to that of the ZIF‐8 biocomposite. This study provides a conceptual nanotechnology for maintaining the flexible conformation and activity of enzymes in situ, while also highlighting the superiority of hydrogen‐bonding scaffolds for regulating enzymatic activity. Recently, Yang et al. constructed an enzyme framework assembled by a biomimetic bottom‐up strategy.^[^ ^188^ ^]^ The biomimetic approach based on the HOF skeleton provided an ultra‐high encapsulation efficiency of CAT while retaining the high crystallinity of the HOF and periodically arranged mesoporous channels.

Although the synthetic design principles of HOFs are similar to those of MOFs and COFs, they are significantly different from other types of framework materials in that they are constructed through hydrogen bonding interactions between organic molecules. The stability of the framework can be enhanced by including other weak interactions such as van der Waals interactions or π–π interactions. Compared with MOFs and COFs, HOFs may have better biocompatibility as carriers for immobilized enzymes because their structures do not contain metal ions and are structurally similar to amino acid chains that make up enzymes.^[^ ^134^, ^189^ ^]^ In addition, HOFs are more easily recoverable and recyclable because they can be repaired by recrystallization strategies.^[^ ^134^ ^]^

#### 5.4.2 Drug Delivery

Drug delivery is an important process in biomedicine and plays an important role in animal health and in vivo therapies. Due to the low cytotoxicity and biocompatibility of HOFs, their unique structural properties allow them to be applied in biomedicine. The porosity of HOFs allows them to encapsulate guest molecules and then controllably release them upon applying an external stimulus. The reversible hydrogen bonds within HOFs can be gradually degraded in specific physiological environments to prevent additional damage.^[^ ^190^ ^]^

Cao et al. self‐assembled a porous drug carrier PFC‐1 with a 1D rhomboid channel (pore size of 18 Å × 23 Å).^[^ ^52^ ^]^ PFC‐1 produced singlet oxygen under visible light illumination, and the anticancer drug doxorubicin (Doxo) was easily loaded into PFC‐1 (26.5 wt.%) and was slowly released in a weakly acidic environment. In vitro experiments showed that Doxo@Nano‐PFC‐1 had very low cytotoxicity and showed excellent chemical‐photodynamic combination therapy in cancer therapy (Hela cells) (Figure 29f,g). Sun et al. also used ultrasonic‐assisted liquid stripping technology to obtain nanoribbons with atomic thickness (nr‐HOF), which showed good dispersion and large surface area ratios in aqueous solutions.^[^ ^190b^ ^]^ nr‐HOF efficiently supported doxorubicin to obtain an nr‐HOF@Doxo drug carrier. Because porphyrin produces singlet oxygen in an oxygen‐containing environment, nr‐HOF@Doxo showed a synergistic effect of chemotherapy‐photodynamic therapy‐photothermal therapy. In vitro cell experiments have shown that this approach is more effective than commercial doxorubicin and shows better anti‐cancer effects, with a cell survival rate as low as 1.3%. Recently, Qu's group designed and synthesized a dual prodrug activation platform (Apt@E‐F@PHOF‐1) based on a biocompatible hydrogen‐bonded organic skeleton. This platform achieved tumor‐selective prodrug activation and prevented the metabolic inactivation of drug molecules synthesized in situ, thereby further enhancing the efficacy of tumor therapy.^[^ ^191^ ^]^ Apt@E‐F@PHOF‐1 was composed of an iron porphyrin‐based biologic orthogonal precatalyst (PHOF‐1), a 5‐fluorouracil prodrug (pro‐5FU), a 5‐acetylidene uracil prodrug (pro‐5EU), and an AS1411 aptamer. A high concentration of glutathione (GSH) at the tumor site reduced iron porphyrins to ferrous porphyrins and then catalyzed the bond cleavage reaction to synthesize 5FU and 5EU. 5FU played an anti‐tumor role, and 5EU inhibited the metabolic enzyme 5FU to inhibit the activity of dihydropyrimidine dehydrogenase (DPD). This prevented the metabolic inactivation of 5FU and improved the bioavailability of 5FU, thereby maintaining a long‐term high concentration of 5FU in the tumor area, and further enhancing tumor inhibition. Apt@E‐F@PHOF‐1 has the characteristics of tumor‐selective dual prodrug activation, which can improve the bioavailability of chemotherapy drugs and reduce their toxic effects on normal tissues. This work provided a new strategy for the use of bioorthogonal chemistry to prevent the metabolic inactivation of drugs. Tang et al. used a combinatorial strategy to self‐assemble proteins with catalytically active HOFs to form biocomposites (RuB‐HOFs), which showed high photocatalytic reduction activity and could enter cells to promote bioorthogonal photoreduction reactions.^[^ ^192^ ^]^ More importantly, RuB‐HOFs encapsulated with catalase generated hydrogen sulfide (H_2_S) in mitochondria via the photocatalytic reduction of Pro‐H_2_S and simultaneously degraded hydrogen peroxide via enzyme catalysis. This provided significant neuroprotective effects against oxidative stress, as well as a multifunctional chemical tool for mitochondria‐targeted bioorthogonal catalysis of pro‐drug activation. The results laid the foundation for therapeutic applications for the treatment of diseases associated with cellular oxidative stress. Yin et al. exploited the cholesterol metabolism environment in the central nervous system (CNS) to design a HOF regulator that could regulate cholesterol metabolism, block the PD‐1/PD‐L1 pathway, and reduce 2B4 expression. It also disrupted the immunosuppressive environment of glioblastoma (GBM) and restored CD8^+^ T cell viability.^[^ ^193^ ^]^ The regulation of cholesterol metabolism was also beneficial for the invasive treatment of GBM, which provided a new strategy for treating GBM and also explored the feasibility of a “metabolic checkpoint” for the treatment of GBM.

#### 5.4.3 Environmental Antibacterial Applications

HOFs also play a role in environmental sterilization/antibacterial by absorbing bacteria, viruses, and other microorganisms, and then catalytically producing strongly‐oxidizing free radicals that kill these microorganisms. Additionally, HOFs exhibit good biocompatibility and can play a long‐term role in organisms without causing harm.^[^ ^29^, ^30^ ^]^

**Figure 30:**
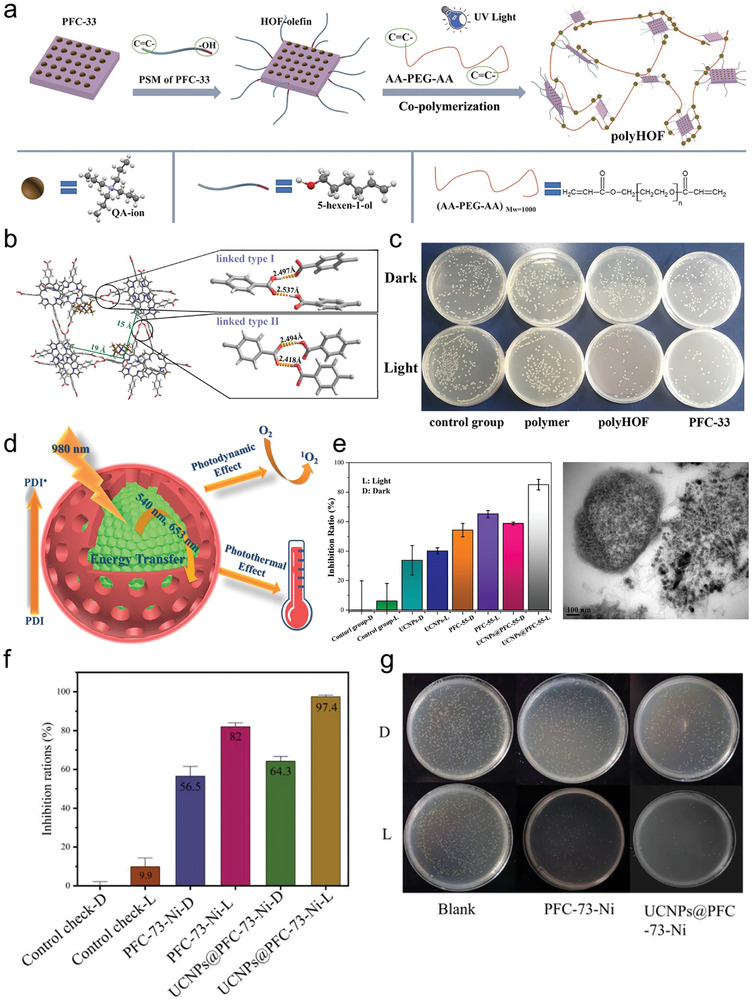
a) Preparation of polyHOF through PSM and subsequent polymerization; b) Two different connection modes between adjacent building blocks and the size of an open channel in PFC‐33; c) Images of *E. coli* on LB agar plates. Reproduced with permission.^[^ ^91^ ^]^ Copyright 2020, Wiley‐VCH. d) RET process from the UCNPs core to the PFC‐55 shell to achieve NIR‐responsive photothermal and photodynamic effects. Reproduced with permission.^[^ ^194^ ^]^ Copyright 2021, Wiley‐VCH. e) Inhibition ratios corresponding to different treatments and a TEM image of *E. coli* cultured with UCNPs@PFC‐55 for 3 h under NIR irradiation (980 nm, 1.5 W cm^−2^). Reproduced with permission.^[^ ^194^ ^]^ Copyright 2021, Wiley‐VCH. f) Inhibition ratios under different treatments. Reproduced with permission.^[^ ^197^ ^]^ Copyright 2024, Wiley‐VCH. g) *Escherichia coli* medium after treatment at 980 nm and 0.5 W cm^−2^. Reproduced with permission.^[^ ^197^ ^]^ Copyright 2024, Wiley‐VCH.

Liu et al. proposed a method to integrate functional substances into a HOF by constructing an anionic skeleton.^[^ ^91^ ^]^ With porphyrin photosensitizers as porous scaffolds and commercial fungicides (quaternary ammonium, QA ions) integrated into the structure as counterbalance ions, PFC‐33 combined photodynamics and chemical antibacterial effects. Due to the presence of porphyrin photosensitizers and quaternary ammonium, PFC‐33 produced ROS and controlled the release of fungicides via the porphyrin skeleton under various physiological conditions. This demonstrated synergistic photodynamic and chemical antibacterial efficiency (**Figure** **30**a–c). Due to interfacial interactions between the free carboxyl groups on the surface of PFC‐33 and the polymer matrix, the polyHOF membrane prepared by PFC‐33 showed high permeability, good stability, and excellent antibacterial activity. This provides a model for expanding the potential applications of HOF membranes. Liu et al. constructed a photothermal and photodynamic HOF (PFC‐55) for sterilization.^[^ ^194^ ^]^ Due to its core‐shell structure, the composite used a near‐infrared (980 nm) light source to initiate resonant energy transfer between the core and shell, thus inhibiting *E. coli* (Figure 30d,e). Ye et al. prepared a series of hydrophilic antibacterial polymers by cross–linking chitosan and polyvinyl alcohol with lauramidopropyl betaine and a hydrogen‐bonded organic skeleton (CS/PVA/LPB/2D‐HOF).^[^ ^195^ ^]^ The antibacterial activity of the mixed membrane reached 95.76%. In vitro and in vivo experiments showed that the mixed membrane promoted tissue regeneration and wound healing. The application of HOFs for bactericidal/antibacterial research is still in the initial stage, and both the mechanism and application prospects still require exploration. Wang et al. prepared broad‐spectrum antibacterial nanofibers (HOF@PVDF‐HFP) by an electrospinning method and then embedded them in photoactive HOF nanocrystals.^[^ ^196^ ^]^ The HOF@PVDF‐HFP nanofibers maintained excellent tensile properties and breathability while protecting the HOF from acid and alkali corrosion. Through optimization experiments, the singlet oxygen generation efficiency of 0.5 wt.% HOF‐101‐F@PVDF‐HFP nanofibers were increased by nearly 2 times and killed viruses, bacteria, and fungi within 30 min. Xiao et al. coated a porous photosensitive HOF onto conversion nanoparticles (UCNPs) to construct a core‐shell structure named UNCPs@PFC‐73‐Ni (Figure 30f,g).^[^ ^197^ ^]^ The Er and Tm co‐doped UCNPs were employed to enhance the spectral overlap and energy conversion efficiency of infrared light into visible light to excite the porphyrin shell. The composites exhibited significant photodynamic and photothermal effects under infrared light and showed significant antimicrobial effects against *Escherichia coli*, highlighting their potential as antimicrobial agents under infrared light irradiation.

## 6 Combining Environmental and Energy Applications

The porosity, stability, and modifiability of HOFs allow them to be used to identify, detect, and even adsorb and convert many complex environmental pollutants. HOFs can also be applied as energy storage and conversion materials (such as battery catalysts, electrode materials, and supercapacitors) to improve their efficiency.

**Figure 31:**
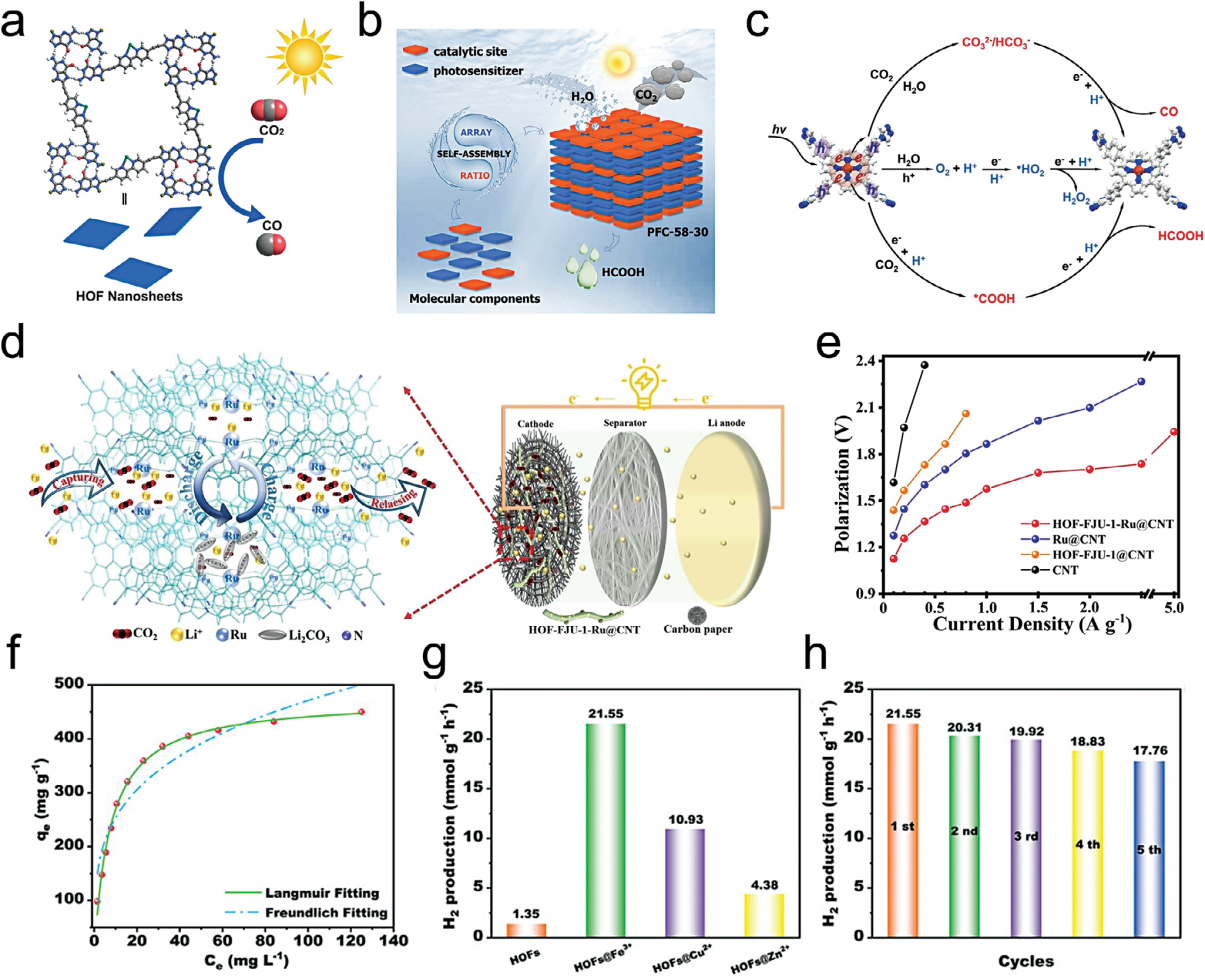
a) Photocatalytic reduction of CO_2_ to CO. Reproduced with permission.^[^ ^116b^ ^]^ Copyright 2023, Wiley‐VCH. b) Photocatalytic CO_2_ reduction to produce HCOOH. Reproduced with permission.^[^ ^123^ ^]^ Copyright 2023, Wiley‐VCH. c) Proposed CO_2_ photoreduction mechanism using PFC‐58‐30. Reproduced with permission.^[^ ^123^ ^]^ Copyright 2023, Wiley‐VCH. d) Schematic diagram of a Li─CO_2_ battery and e) battery overpotentials at various current densities. Reproduced with permission.^[^ ^199^ ^]^ Copyright 2023, Wiley‐VCH. f) Adsorption isotherms of BPA onto HOFs@Fe^3+^ and non‐linear fits of Langmuir and Freundlich models, g) apparent photocatalytic H_2_ evolution rates, and h) H_2_ generation rate over five successive cycles by HOFs@Fe^3+^. Reproduced with permission.^[^ ^200^ ^]^ Copyright 2023, Wiley‐VCH.

**Figure 32:**
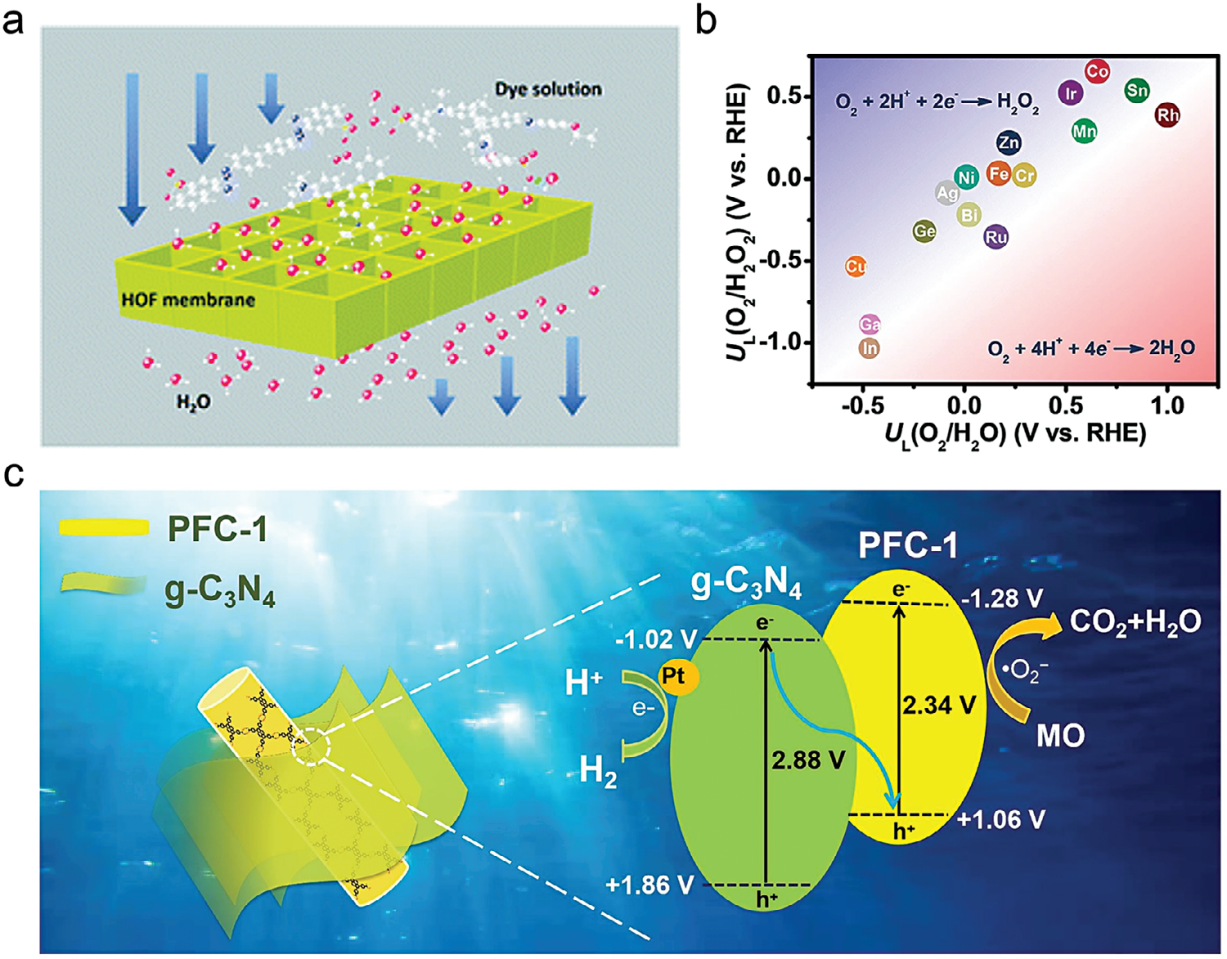
Coupling of energy conversion and pollutant degradation applications of HOFs. a) Constructed membranes of HOFs for dye separation to obtain pure water. Reproduced with permission.^[^ ^177^ ^]^ Copyright 2021, Royal Society of Chemistry. b) 2D map showing the thermodynamic limiting potential of different metalloporphyrins for the 2e‐ORR and 4e‐ORR. Reproduced with permission.^[^ ^201^ ^]^ Copyright 2022, Springer Nature. c) Photocatalytic degradation of methyl orange by PFC‐1/CNNS. Reproduced with permission.^[^ ^182a^ ^]^ Copyright 2022, Elsevier.

However, it is unclear how HOFs combine environmental and energy applications to reduce environmental pollution and ecological damage, promote the sustainable development of energy, optimize energy structures, use and recycle resources, and provide economic and social benefits. Giving play to the bridge role and dual characteristics of HOFs for environmental and energy applications and coupling explorations of the two is expected to improve energy utilization and reduce environmental pollution. To this end, we summarized the latest HOF‐based material research at the intersection of these two broad fields (**Figures** **31** and **32**).

### 6.1 Materials for Converting Pollutants into Energy

In the past, HOF‐based materials were designed for only a single application such as pollutant conversion or energy storage and conversion.^[^ ^7^, ^29^, ^134^ ^]^ The idea of converting pollutants into energy or resources has not been widely investigated, but this has better advantages for improving the efficiency of pollutant conversion and energy production. In the following case studies, we show HOF‐based materials for the co‐conversion of pollutants and the production of energy substances.

HOFs containing metal porphyrins designed by Qi et al. photocatalyzed the reduction of CO_2_ to CO and also converted greenhouse gases into energy substances.^[^ ^56^ ^]^ Yu et al. prepared Ni‐doped HOF nanosheets (HOF‐25‐Ni), which showed selective CO_2_‐to‐CO conversion under visible‐light irradiation (Figure 31a).^[^ ^116b^ ^]^ Zhou et al. proposed a photocatalytic process dominated by micropore‐restricted exciton transfer.^[^ ^198^ ^]^ In this study, the photocatalytic H_2_ yield of HOF‐H4TBAPy reached 358 mmol h^−1^ g^−1^, and the molar hydrogen production reached in a 0.5 m^2^ plate reactor. This provided an important idea for exploring environmentally benign photocatalytic hydrogen production. In addition, Cao's research group found that a novel HOF (PFC‐58‐30) catalytically converted CO_2_ to produce energy‐related HCOOH (Figure 31b,c).^[^ ^123^ ^]^ By designing a stable HOF‐based multi‐functional electrocatalyst, Zhang's research group achieved the long‐term stable cyclic operation of a Li─CO_2_ battery at a high rate.^[^ ^199^ ^]^ The HOF‐based multifunctional catalyst was composed of a highly stable HOF (HOF‐FJU‐1) and ruthenium nanoparticle‐modified carbon nanotubes (Figure 31d). The introduction of HOF‐FJU‐1 improved the transmission capacity of the catalyst for CO_2_ and Li^+^ so that the efficient catalytic conversion of CO_2_ was achieved. HOF‐FJU‐1 maintained a low overpotential of 1.96 V at a high current density of 5 A g^−1^ (Figure 31e). This work provides an important reference for the development of Li─CO_2_ battery catalysts based on HOFs and also broadens the applications of HOF in the field of environmental pollution transformation and energy storage. Yang et al. prepared a porous nanomaterial HOFs@Fe^3+^, in which Fe^3+^ was anchored to HOFs by electrostatic interactions and coordination.^[^ ^200^ ^]^ Its high specific surface area and hydrogen bonding sites strongly interacted with the OH groups of bisphenol A (BPA), resulting in a high adsorption capacity (452 mg g^−1^) (Figure 31f). Its ordered structure and Fe^3+^ promoted photogenerated carrier separation. HOFs@Fe^3+^ adsorbed and photodegraded 50 ppm of BPA within 20 min via a combination of adsorption and photodegradation. With Fe^3+^ as the cocatalyst, the photocatalytic hydrogen production efficiency reached 21.55 mmol g^−1^ h^−1^ (Figure 31g). This tri‐functional material with a high adsorption capacity, photodegradation efficiency, and photocatalytic H_2_ production activity may solve the long‐standing trade‐off between environment and energy (Figure 31h).^[^ ^200^ ^]^

### 6.2 Efficient Use of Energy for Pollutant Disposal

The simultaneous realization of efficient and environmentally friendly energy utilization and pollutant degradation has become a popular research topic. However, there is little research on the use of HOFs to degrade the coupled pollutants for energy use. It is of great practical significance to stimulate this innovative idea to solve the energy crisis and ensure environmental protection.

Jiang et al. prepared a thin‐film nanocomposite membrane (TFN) based on porous HOF (PFC‐1) nanoparticles.^[^ ^177^ ^]^ Due to the ultra‐thin and highly‐ordered porous structure of PFC‐1 nanoparticles, the TFN could separate dyes and also showed ultra‐high water permeability (Figure 32a).^[^ ^177^ ^]^ This idea can be used to develop materials with combined dye separation and degradation functions. Li et al. reported the catalytic reduction of O_2_ to H_2_O_2_ by a cobaltoporphyrinic HOF.^[^ ^201^ ^]^ Several hydrogen‐bonding metal porphyrin frameworks with similar topologies but different metal centers were prepared by high‐throughput screening. Cobalt porphyrin showed the best 2e‐ORR activity and selectivity, with an H_2_O_2_ selectivity of > 90% over a wide potential window, and a TOF of 10.9 s^−1^ at 0.55 V in an acidic environment, which exceeded the noble metal benchmark. The produced H_2_O_2_ is a common oxidizer in traditional advanced oxidation processes and could be used for the oxidative removal of various pollutants (Figure 32b). Shi et al. synthesized a novel all‐organic Z‐scheme HOF/g‐C_3_N_4_ nanosheets (CNNS) heterojunction photocatalyst through an in situ electrostatic method.^[^ ^182a^ ^]^ Combing the complementary advantages of HOFs and CNNS, the fabricated Z‐scheme HOFs/CNNS heterojunction inhibited photoinduced electron‐hole recombination, and more charge carriers were accumulated to produce highly reactive substances (•O^2−^, •OH, and h^+^). It also exhibited a high photocatalytic hydrogen evolution rate of 4450 µmol h^−1^g^−1^ with an apparent quantum efficiency (AQY) of 22% at 450 nm. More importantly, this heterojunction degraded 100% of MO within 60 min (Figure 32c).

## 7 Future Challenges and Development Strategies

**Figure 33:**
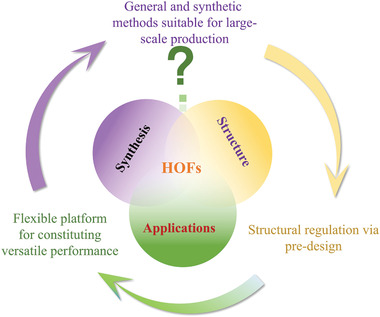
HOFs in the application of structural and functional challenges, and its expected direction of development concepts.

**Figure 34:**
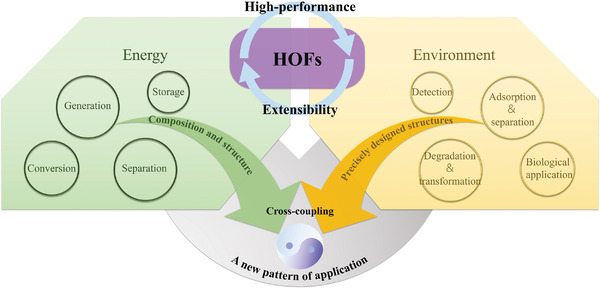
Overview of the application of HOFs in energy and environment and its coupling development ideas.

This section reviews the major challenges faced by HOF‐based materials in structural regulation and environment‐energy dual applications and potential development strategies to aid in the structural design and cross‐applications of HOFs. (**Figures** **33** and **34**).

### 7.1 Structural and Functional Challenges and Development Opportunities

The unique structural characteristics of HOFs have spurred rapid advancements in their synthesis and preparation strategies. However, when compared with COFs and MOFs, HOFs still encounter several challenges. The biggest challenge is the weak force and poor directionality of hydrogen bonds, which makes it challenging to construct stable structures and functions.^[^ ^7^, ^179^ ^]^ The structure of HOFs quickly collapses after removing guest solvent molecules. Additionally, most HOFs are synthesized using a single organic unit, but changing the synthesis conditions or solvents can change the structure, posing challenges for the precise synthesis of target structures.^[^ ^7^, ^29^ ^]^ Furthermore, the formation process of HOF‐based materials and their composition/structural properties lack in‐depth understanding. Theoretical calculation methods will play an important role in future explorations.^[^ ^20^, ^25^ ^]^ Therefore, a systematic review of the difficulties faced by HOFs in terms of their structural and functional applications will play a key role in helping overcome these technical barriers and promoting the development of HOFs.

#### 7.1.1 Exploring the Feasibility of General Adaptations to Achieve Large‐Scale Production

Due to the poor directionality and weak forces of hydrogen bonds, the precise synthesis of HOFs remains challenging. Due to the poor directionality and weak forces of hydrogen bonds, the precise synthesis of HOFs remains challenging. In theory, adjustments to the pore shape and size, as well as modifications to the pore surface, are possible. However, these modifications are still in the development stage as extending building units or introducing other functional groups easily leads to structural changes.^[^ ^27a^ ^]^ HOFs can obtain different structures under different synthesis conditions (different solvents), which affects the precise synthesis of target structures.^[^ ^30^ ^]^ Different structures can even be formed by changing the external conditions, which prevents understanding the structure‐performance relationships of HOFs.

The large‐scale preparation of HOFs with single‐crystal morphologies remains a significant challenge.^[^ ^202^ ^]^ The identification and characterization of HOF single‐crystal structures, along with the development of related methods, are imperative. Current synthesis efforts are often in the laboratory stage, plagued by drawbacks such as low yields and the generation of waste gas and liquids. It is important to effectively reuse ligands, solvents, and by‐products during synthesis. Existing synthesis methods are often complex and require multiple steps and are un‐optimized. Synthesis strategies similar to one‐pot self‐assembly methods and universal methods need to be developed. Although the preparation process of self‐templated HOFs is expected to enable their large‐scale production, existing strategies are still limited to single small systems. Additionally, there is also a lack of understanding of the formation process of derivative materials and their composition.^[^ ^203^ ^]^ Multiple synthesis strategies may be an effective method to solve this challenge, but related research is still lacking.

#### 7.1.2 Pre‐design Control to Accurately Construct Multi‐Functional HOFs

The pre‐design and site control of HOFs are necessary to accurately and rationally construct HOFs. The selection of supramolecular materials and construction modes is important for the construction of HOFs. Although network synthetic chemistry has been used to predict and design HOFs with specific structures, the precise preparation of HOFs with target networks remains challenging due to the low bond energy of hydrogen bonds.^[^ ^7^, ^29^ ^]^ The reasonable selection of structures with rigid skeletons, spatial conformations, and cooperative non‐covalent interactions is one solution.^[^ ^7^, ^204^ ^]^ In the design and control of structure‐oriented synthesis, the stiffness of the main chain, the strength and number of hydrogen bonding sites, extending the direction of hydrogen bonds, and interactions between structural units and guest molecules should be comprehensively considered. This will facilitate the synthesis of highly stable multi‐functional applied HOFs.

Many synthesized HOFs still lack functional groups, such as unsaturated coordination sites, Lewis acids, and Brønsted acid sites.^[^ ^202^ ^]^ This deficiency makes it challenging to modify and control HOFs after their synthesis. Therefore, pre‐engineering to introduce strong binding sites, such as metal sites or Lewis acid/base sites, into the pores of HOFs may provide an early‐stage molecular design strategy. In addition, the combination of machine learning and high‐throughput crystal screening will undoubtedly accelerate the construction of precisely designed, functionally‐specific HOFs. There is a need to develop simple and reliable algorithms to reduce computational costs and make them practical and universal.

Most HOFs have very small pores, potentially limiting the mass transport of molecules through the channels. Additionally, the lack of metals in most HOFs hinders their applications in heterogeneous catalysis.^[^ ^202^ ^]^ To address these challenges, pre‐engineering and encapsulating catalytically active groups into stable porous HOFs could offer solutions and open up new avenues for exploring functional materials.^[^ ^30^, ^146^ ^]^ Fluorination can increase the hydrophobicity and stability of HOFs, so pre‐designing fluorinated structures between building units or guest molecules may promote the development of functional HOFs.^[^ ^5a^ ^]^ A balance between stability and flexibility plays an important role in the development of dual‐performance materials. The stronger the rigidity of the molecule, the more difficult it is to change its directionality. More benzene rings can be pre‐selected to enhance the rigidity of the main chain of a molecule to design a sliding or scalable structure with multiple applications. Looking ahead, overcoming the challenges of preparing HOFs with high porosity, large specific surface area, and good stability is expected through pre‐structural design or site control engineering.

#### 7.1.3 The Unique Properties of HOFs Provide a Platform for Multi‐Structure Functionalization

HOFs provide a design platform for the preparation of mild, easy‐to‐purify, and multi‐functional crystalline materials. There have been many attempts to construct hybrid HOFs, such as entities with robust, high‐dimensional configurations, and sufficient non‐coplanar directional extension. These are likely to create stable porous hydrogen‐bonded networks. In addition, the functionalization of hybrid HOFs requires sufficient metal coordination and hydrogen bonding sites.^[^ ^205^ ^]^ More attempts are being made to construct hybrid HOFs using a metal core (purines and their derivatives) whose diverse metal complexation modes produce abundant high‐dimensional stereostructures and sufficient secondary building units extending in non‐coplanar directions. These are ideal building units for hybrid HOFs. Multi‐functional groups and unique structural properties leave space for the functionalization of metal cores. The controllable design and synthesis, directional modification, and functionalization of hybrid HOFs are worthy of study. The structure and applications of hybrid HOFs provide an attractive platform for designing novel functional crystalline materials.

HOFs have been developed from material design synthesis and performance evaluation to the establishment of structure‐activity relationships and multi‐functional composite development.^[^ ^206^ ^]^ They have gradually become a powerful platform for developing more interesting and attractive application‐oriented multi‐functional materials. With the continuous development of building units, the structures of HOFs are becoming continuously enriched, and their stability and diversification of functions are being gradually realized.

### 7.2 Application Challenges of Energy and Environment and their Coupled Development Directions

HOFs have a wide range of applications in energy or environment‐related fields, such as electrochemical energy storage, the production and separation of energy substances, gas adsorption and separation, ion screening, sensing, and fluorescence detection, photocatalysis, pollutant identification and transformation, bioenzymology, and medical applications.^[^ ^7^, ^29^, ^202^ ^]^ As HOFs continue to undergo development, we are likely to witness a revolution in the coupling between environmental protection and renewable energy generation. However, it's crucial to note that the development and applications of HOFs are still in their early stage compared with more mature MOFs and COFs.^[^ ^183^ ^]^ Despite the existing challenges, numerous potential opportunities exist. In the future, it is necessary to strengthen research on the coupling of environment and energy applications to fully explore the advantages of HOF‐based construction materials and their cross‐applications.

#### 7.2.1 The Development of Application‐oriented HOFs with High Performance and Extensibility is Still the Focus of Future Work

Although HOF membranes have many advantages, the development of HOF membranes is greatly constrained by weak hydrogen bond forces and poor directionality.^[^ ^207^ ^]^ Additionally, the challenging synthesis process and high synthesis costs have hindered large‐scale commercial applications, relegating them primarily to scientific research.^[^ ^7d^ ^]^ However, as production costs decrease, HOF membranes hold promising development prospects, especially HOF films that can maintain their crystalline structures under strong acid, strong alkali, high temperature, boiling water, and other conditions.^[^ ^206^ ^]^ With the anticipated reduction of manufacturing costs, HOF films are expected to see expanded applications for energy storage, especially in the production, separation, and transformation of environmental pollutants. This, in turn, can contribute to the realization of carbon neutrality.

Coupling the structure‐function relationship and applications is the future research direction of HOFs. HOFs/polymer hybrid materials show versatility, but the instability of hydrogen bonds poses a challenge to their precise manufacturing.^[^ ^30^ ^]^ Therefore, there is a need to expand the design and synthesis of functional HOFs to develop multi‐component HOFs. It is worth considering that integrating the respective advantages of MOFs and COFs and assisting the collaborative construction of HOF‐based materials, can be used to develop synergistic porous network materials to achieve versatile applications in complex environments. For example, networks with multiple topologies create diverse pathways and coupling opportunities for building combined COFs and HOFs. In general, the accessibility of chelated metal sites to reactants in organic ligands can be enhanced in 3D topologies due to their open porous structure. In contrast, the high overlap of planar conjugates with 2D topologies can promote interlayer charge transfer, but this masks metal active sites. While there is limited research on methods to control their topology, it is believed that properly constructing COFs and HOFs with 3D structures with high connectivity, strong stability, and unburied active sites can improve their photocatalytic efficiency.

Analysis of the synthesis process and internal mechanism of HOF structures is important for synthesizing materials for specific environment and energy applications.^[^ ^183^ ^]^ Interactions between the adsorbent and adsorption sites on the inner surface of adsorption channels were elucidated by theoretical calculation and molecular simulations. Microscopic information that was difficult to obtain experimentally was obtained, which facilitated the synthesis and applications of actual materials. Therefore, these new techniques are expected to screen which HOFs have adsorption properties for specific pollutants and explain their adsorption mechanism at the molecular level. In practical applications, a balance between the stability and functionality of HOF characteristics needs to be modulated, and structure‐performance‐application relationships need to be further explored to better serve efficient applications in energy, environment, and other fields.

#### 7.2.2 HOFs Present Many Challenges and Opportunities in the Energy Sector

By adjusting their composition and structure, HOFs can form large frameworks with adjustable compositions and abundant functional groups, making them excellent electrochemical catalysts.^[^ ^206^ ^]^ These properties allow HOF‐based catalysts to optimize the utilization of atoms and molecules, facilitating storage and transport of charges and substances during electrochemical processes. However, the insulating properties of pure HOFs currently limit their applicability as electrode materials and electrocatalysts. Future research should prioritize the design of HOFs with high electron conductivity. In addition, the structural stability of HOFs during ion embedding and transformation is a key concern, which requires an in‐depth analysis of the structure‐activity relationship combined with theoretical calculations and electrochemical characterization. Self‐templating HOF derivatives can be used to synthesize porous/hollow microstructures/nanostructures to couple metallic parts and carbon nanomaterials for electrochemical applications. Nevertheless, research on HOFs for electrocatalysis applications is still in its early stages and requires further development. The progress of HOF‐related materials for electrochemistry is an interdisciplinary process with both opportunities and challenges. There is still a long way to go from material design synthesis, performance evaluation, and structure‐activity relationship establishment to final practical applications. Currently, research has only been performed at the laboratory scale, with no reports on the synthesis of materials at industrial scales or the development of functional devices. With the continuous deepening of research in related fields, we are likely to witness a revolution in the utilization of renewable energy in the near future.

#### 7.2.3 HOFs for Environmental Applications are Still in Their Infancy

With the periodic network chemical structure of HOFs, different functional groups can be selected to obtain different functions. The structure of HOFs can be pre‐designed by constructing structures such as aromatics, aliphatic groups, esters, carboxylic acids, and alcohols, to precisely regulate their structural and functional properties.^[^ ^42^ ^]^ Different functional groups allow HOFs to be used in specific areas, such as increasing interactions between HOFs and pollutants, such as the selective separation or removal of pollutants from gases or solutions.^[^ ^78^ ^]^ Although HOFs show great potential for heterophase catalysis, their morphologies have not been systematically studied from the nanoscale to the microscale. The introduction of metal nanoparticles into the nanopores of HOFs can greatly broaden and enhance their applications. However, the coordination interactions between metal ions and hydrogen bonding sites may form metal‐ligand complexes that affect the phase purity of HOFs. Exploring this trade‐off still requires research.

Because of the effect of modification on photocatalytic efficiency and product selectivity, photocatalysis needs to be studied, especially HOFs for molecular engineering, such as D‐A systems and π–π interactions.^[^ ^208^ ^]^ To this end, more comprehensive data from computational simulations and machine learning models will aid in the structural and molecular design and synthesis of multifunctional HOFs. For example, molecular‐level design can improve the light collection, charge transfer, and catalysis of the resulting materials.^[^ ^209^ ^]^ HOFs need to expand beyond traditional water and air pollution applications into new areas, such as the remediation of contaminated soil.^[^ ^78^ ^]^ The remediation of contaminated soil is an urgent environmental problem that may damage entire ecosystems through the biological chain and endanger human health. HOFs can separate pollutants from soil by adsorption or render them soluble using chemical reducing agents, thereby limiting their migration and bioavailability in soil. When used in bioenvironmental applications, the stability and safety of HOFs must be carefully studied in vitro and in vivo.^[^ ^145^ ^]^ The long‐term biosafety of HOFs also needs to be evaluated for biomedical applications. Most reported HOFs have been in the millimeter or micron scale, and it is challenging to shrink stacked HOFs to nanoscale materials using current technologies. The preparation of nanoscale HOFs through top‐down or bottom‐up strategies will greatly promote their biological and clinical applications.

#### 7.2.4 Cross‐Disciplinary Development of HOFs for Energy and Environmental Applications

HOFs show rich pore structures, high specific surface areas, good crystallinity, flexibility, molecular selective recognition, biocompatibility, low cytotoxicity, self‐healing, recrystallization, recycling, and reusability.^[^ ^7^, ^29^ ^]^ Using these diverse characteristics must be considered for future applications, especially coupling their environment and energy applications to meet the needs of more complex practical applications. The use of HOF‐based materials to couple pollutant removal and energy utilization provides a novel material idea for solving environmental pollution and energy crises. It is expected to achieve pollutant removal, resource recycling, and bring economic and social benefits.

HOFs can act as a bridge between environment and energy applications to realize coupled explorations of the two. This material design concept should be employed to address scientific challenges in cross‐applications of environment and energy. The analysis should focus on how the uniqueness and advantages of HOFs are seamlessly integrated into material structures, facilitating a rapid and accurate assessment of the rationality and compatibility of structure‐function design. This can be accomplished by coupling high‐throughput theoretical calculations and experiments. The idea of cross‐coupled applications of HOFs in energy and environment has been echoed in both fields, but its development still faces various challenges.

## 8 Conclusion

HOFs are crystalline organic porous materials that show more advantages than other materials. This paper systematically summarizes the latest cutting‐edge work in terms of the basic characteristics, structural design, performance control, synthesis methods, and applications of HOFs. Providing a clear theoretical reference for the underlying construction logic, stability control, and multi‐functional design of HOFs. Significantly, it comprehensively reviews research progress in the energy and environmental applications of HOFs for the first time. HOFs demonstrate promising application prospects across various domains, including energy separation and storage, energy conversion, and utilization, energy resource production, pollutant detection, pollutant adsorption and separation, pollutant degradation and transformation, and biological applications. Based on the strong attraction of HOFs in the coupling of energy and environment, we propose collaborative cross‐disciplinary ideas and offer material application guidance for the conversion of environmental pollutants coupled with energy resources. This highlights a powerful new direction for cross‐disciplinary research of HOFs. This paper is anticipated to catalyze extensive applications of HOFs in energy, environment, and their interdisciplinary fields, fostering further exploration of their potential applications.

## Conflict of Interest

The authors declare no conflict of interest.

## Supporting information

Supporting Information

## References

[1] a) T. L. Easun , F. Moreau , Y. Yan , S. Yang , M. Schröder , Chem. Soc. Rev. 2017, 46, 239; 27896354 10.1039/c6cs00603e

[2] a) Z. J. Lin , J. Lü , M. Hong , R. Cao , Chem. Soc. Rev. 2014, 43, 5867; 24699533 10.1039/c3cs60483g

[3] a) N. W. Ockwig , O. Delgado‐Friedrichs , M. O'Keeffe , O. M. Yaghi , Acc. Chem. Res. 2005, 38, 176; 15766236 10.1021/ar020022l

[4] Z. Wang , S. Zhang , Y. Chen , Z. Zhang , S. Ma , Chem. Soc. Rev. 2020, 49, 708. 31993598 10.1039/c9cs00827f

[5] a) X. Y. Song , Y. Wang , C. Wang , D. Wang , G. W. Zhuang , K. O. Kirlikovali , P. Li , O. K. Farha , J. Am. Chem. Soc. 2022, 144, 10663; 35675383 10.1021/jacs.2c02598

[6] H. Furukawa , K. E. Cordova , M. O'Keeffe , O. M. Yaghi , Science 2013, 341, 1230444. 23990564 10.1126/science.1230444

[7] a) B. Wang , X. L. Lv , J. Lv , L. Ma , R. B. Lin , H. Cui , J. Zhang , Z. Zhang , S. Xiang , B. Chen , Chem. Commun. 2020, 56, 66; 10.1039/c9cc07802a 31790104

[8] T. S. Moore , T. F. Winmill , J. Chem. Soc., Trans. 1912, 101, 1635.

[9] W. M. Latimer , W. H. Rodebush , J. Am. Chem. Soc. 1920, 42, 1419.

[10] J. D. Watson , F. H. C. Crick , Nature 1953, 171, 737. 13054692 10.1038/171737a0

[11] E. Arunan , G. R. Desiraju , R. A. Klein , J. Sadlej , S. Scheiner , I. Alkorta , D. C. Clary , R. H. Crabtree , J. J. Dannenberg , P. Hobza , H. G. Kjaergaard , A. C. Legon , B. Mennucci , D. J. Nesbitt , Pure Appl. Chem. 2011, 83, 1637.

[12] D. J. Duchamp , R. E. Marsh , Acta Crystallogr. B. 1969, 25, 5.

[13] F. H. Herbstein , M. Kapon , G. M. Reisner , J. Inclusion Phenom. Mol. Recognit. Chem. 1987, 5, 211.

[14] O. Ermer , J. Am. Chem. Soc. 1988, 110, 3747.

[15] a) M. Simard , D. Su , J. D. Wuest , J. Am. Chem. Soc. 1991, 113, 4696;

[16] B. F. Hoskins , R. Robson , J. Am. Chem. Soc. 1989, 111, 5962.

[17] O. M. Yaghi , H. Li , J. Am. Chem. Soc. 1995, 117, 10401.

[18] A. P. Côté , A. I. Benin , N. W. Ockwig , M. O'Keeffe , A. J. Matzger , O. M. Yaghi , Science 2005, 310, 1166. 16293756 10.1126/science.1120411

[19] Y. He , S. Xiang , B. Chen , J. Am. Chem. Soc. 2011, 133, 14570. 21863860 10.1021/ja2066016

[20] a) M. Mastalerz , I. M. Oppel , Angew. Chem., Int. Ed. 2012, 51, 5252; 10.1002/anie.201201174 22473702

[21] a) F. Hu , C. Liu , M. Wu , J. Pang , F. Jiang , D. Yuan , M. Hong , Angew. Chem., Int. Ed. 2017, 56, 2101; 10.1002/anie.201610901 28090721

[22] T. H. Chen , I. Popov , W. Kaveevivitchai , Y. C. Chuang , Y. S. Chen , O. Daugulis , A. J. Jacobson , O. Š. Miljanić , Nat. Commun. 2014, 5, 5131. 25307413 10.1038/ncomms6131

[23] Q. Chen , Y. Zhao , B. H. Han , Chin. Sci. Bull. 2013, 58, 2352.

[24] a) Q. Huang , W. Li , Z. Mao , L. Qu , Y. Li , H. Zhang , T. Yu , Z. Yang , J. Zhao , Y. Zhang , M. P. Aldred , Z. Chi , Nat. Commun. 2019, 10, 3074; 31300644 10.1038/s41467-019-10575-5 PMC6625987

[25] P. Cui , D. P. McMahon , P. R. Spackman , B. M. Alston , M. A. Little , G. M. Day , A. I. Cooper , Chem. Sci. 2019, 10, 9988. 32055355 10.1039/c9sc02832c PMC6991173

[26] a) W. Yang , A. Greenaway , X. Lin , R. Matsuda , A. J. Blake , C. Wilson , W. Lewis , P. Hubberstey , S. Kitagawa , N. R. Champness , M. Schröder , J. Am. Chem. Soc. 2010, 132, 14457; 20866087 10.1021/ja1042935

[27] a) A. Karmakar , R. Illathvalappil , B. Anothumakkool , A. Sen , P. Samanta , A. V. Desai , S. Kurungot , S. K. Ghosh , Angew. Chem., Int. Ed. 2016, 55, 10667; 10.1002/anie.201604534 27464784

[28] W. Yang , J. W. Wang , H. L. Wang , Z. B. Bao , J. C. G. Zhao , B. L. Chen , Cryst. Growth Des. 2017, 17, 6132.

[29] a) L. Chen , B. Zhang , L. Chen , H. Liu , Y. Hu , S. Qiao , Mater. Adv. 2022, 3, 3680;

[30] Y. Liu , G. Chang , F. Zheng , L. Chen , Q. Yang , Q. Ren , Z. Bao , Chem‐Eur. J. 2023, 29, e202202655. 36414543 10.1002/chem.202202655

[31] H. Jiang , D. Alezi , M. Eddaoudi , Nat. Rev. Mater. 2021, 6, 466.

[32] Q. Zhu , J. Johal , D. E. Widdowson , Z. Pang , B. Li , C. M. Kane , V. Kurlin , G. M. Day , M. A. Little , A. I. Cooper , J. Am. Chem. Soc. 2022, 144, 9893. 35634799 10.1021/jacs.2c02653 PMC9490843

[33] E. O. Pyzer‐Knapp , L. Chen , G. M. Day , A. I. Cooper , Sci. Adv. 2021, 7, eabi4763. 34389543 10.1126/sciadv.abi4763 PMC8363149

[34] I. Hisaki , Y. Suzuki , E. Gomez , B. Cohen , N. Tohnai , A. Douhal , Angew. Chem., Int. Ed. 2018, 57, 12650. 10.1002/anie.201805472 29885200

[35] H. W. H. Lai , R. A. Wiscons , C. A. Zentner , M. Zeller , J. L. C. Rowsell , Cryst. Growth Des. 2016, 16, 821.

[36] Y. Wang , K. Ma , J. Bai , T. Xu , W. Han , C. Wang , Z. Chen , K. O. Kirlikovali , P. Li , J. Xiao , O. K. Farha , Angew. Chem., Int. Ed. 2022, 61, e202115956. 10.1002/anie.202115956 34931436

[37] I. Hisaki , N. Q. Emilya Affendy , N. Tohnai , CrystEngComm 2017, 19, 4892.

[38] W. Yan , X. Yu , T. Yan , D. Wu , E. Ning , Y. Qi , Y. F. Han , Q. Li , Chem. Commun. 2017, 53, 3677. 10.1039/c7cc00557a 28265598

[39] J. K. Gao , Y. L. Cai , X. F. Qian , P. X. Liu , H. Wu , W. Zhou , D. X. Liu , L. B. Li , R. B. Lin , B. L. Chen , Angew. Chem., Int. Ed. 2021, 60, 20400. 10.1002/anie.202106665 34219344

[40] H. L. Wang , B. Li , H. Wu , T. L. Hu , Z. Z. Yao , W. Zhou , S. C. Xiang , B. L. Chen , J. Am. Chem. Soc. 2015, 137, 9963. 26214340 10.1021/jacs.5b05644

[41] L. Ma , Y. Xie , R. S. H. Khoo , H. Arman , B. Wang , W. Zhou , J. Zhang , R. B. Lin , B. Chen , Chem‐Eur. J. 2022, 28, e202104269. 34982835 10.1002/chem.202104269

[42] J. Luo , J. W. Wang , J. H. Zhang , S. Lai , D. C. Zhong , CrystEngComm 2018, 20, 5884.

[43] P. Li , Y. He , J. Guang , L. Weng , J. C.‐G. Zhao , S. Xiang , B. Chen , J. Am. Chem. Soc. 2014, 136, 547. 24392725 10.1021/ja4129795

[44] H. Wang , H. Wu , J. Kan , G. Chang , Z. Yao , B. Li , W. Zhou , S. Xiang , J. Cong‐Gui Zhao , B. Chen , J. Mater. Chem. A 2017, 5, 8292.

[45] Y. S. Yang , L. B. Li , R. B. Lin , Y. X. Ye , Z. Z. Yao , L. Yang , F. H. Xiang , S. M. Chen , Z. J. Zhang , S. C. Xiang , B. L. Chen , Nat. Chem. 2021, 13, 933. 34239085 10.1038/s41557-021-00740-z

[46] P. Li , Y. B. He , Y. F. Zhao , L. H. Weng , H. L. Wang , R. Krishna , H. Wu , W. Zhou , M. O'Keeffe , Y. Han , B. L. Chen , Angew. Chem., Int. Ed. 2015, 54, 574. 10.1002/anie.201410077 25394888

[47] J. Nicks , S. A. Boer , N. G. White , J. A. Foster , Chem. Sci. 2021, 12, 3322. 34164102 10.1039/d0sc06906j PMC8179369

[48] K. E. Maly , E. Gagnon , T. Maris , J. D. Wuest , J. Am. Chem. Soc. 2007, 129, 4306. 17358060 10.1021/ja067571x

[49] K. K. Ma , P. Li , J. H. Xin , Y. W. Chen , Z. J. Chen , S. Goswami , X. F. Liu , S. Kato , H. Y. Chen , X. Zhang , J. Q. Bai , M. C. Wasson , R. R. Maldonado , R. Q. Snurr , O. K. Farha , Cell Rep. Phys. Sci. 2020, 1, 100024.

[50] C. A. Zentner , H. W. H. Lai , J. T. Greenfield , R. A. Wiscons , M. Zeller , C. F. Campana , O. Talu , S. A. FitzGerald , J. L. C. Rowsell , Chem. Commun. 2015, 51, 11642. 10.1039/c5cc04219d 26099041

[51] Z. J. Lin , J. Y. Qin , X. P. Zhan , K. C. Wu , G. J. Cao , B. L. Chen , ACS Appl. Mater. Interfaces 2022, 14, 21098. 35482947 10.1021/acsami.2c05176

[52] Q. Yin , P. Zhao , R. J. Sa , G. C. Chen , J. Lu , T. F. Liu , R. Cao , Angew. Chem., Int. Ed. 2018, 57, 7691. 10.1002/anie.201800354 29696754

[53] D. Inokuchi , Y. Hirao , K. Takahashi , K. Matsumoto , H. Mori , T. Kubo , J. Phys. Chem. C 2019, 123, 6599.

[54] I. Hisaki , Y. Suzuki , E. Gomez , Q. Ji , N. Tohnai , T. Nakamura , A. Douhal , J. Am. Chem. Soc. 2019, 141, 2111. 30615836 10.1021/jacs.8b12124

[55] X. Z. Luo , X. J. Jia , J. H. Deng , J. L. Zhong , H. J. Liu , K. J. Wang , D. C. Zhong , J. Am. Chem. Soc. 2013, 135, 11684. 23885835 10.1021/ja403002m

[56] Q. Yin , E. V. Alexandrov , D. H. Si , Q. Q. Huang , Z. B. Fang , Y. Zhang , A. A. Zhang , W. K. Qin , Y. L. Li , T. F. Liu , D. M. Proserpio , Angew. Chem., Int. Ed. 2022, 61, e202115854. 10.1002/anie.202115854 34877789

[57] P. H. Li , P. Li , M. R. Ryder , Z. C. Liu , C. L. Stern , O. K. Farha , J. F. Stoddart , Angew. Chem., Int. Ed. 2019, 58, 1664. 10.1002/anie.201811263 30548232

[58] B. Q. Yu , S. B. Geng , H. L. Wang , W. Zhou , Z. J. Zhang , B. L. Chen , J. Z. Jiang , Angew. Chem., Int. Ed. 2021, 60, 25942. 10.1002/anie.202110057 34499385

[59] Q. Y. Huang , W. L. Li , Z. Mao , H. Zhang , Y. Li , D. Y. Ma , H. Y. Wu , J. Zhao , Z. Y. Yang , Y. Zhang , G. Li , M. P. Aldred , Z. G. Chi , Chem‐Us 2021, 7, 1321.

[60] S. Suku , R. Ravindran , J. Mol. Struct. 2021, 1226, 129314.

[61] S. A. Boer , P. X. Wang , M. J. MacLachlan , N. G. White , Cryst. Growth Des. 2019, 19, 4829.

[62] S. Zieba , A. Gzella , A. T. Dubis , A. Lapinski , Cryst. Growth Des. 2021, 21, 3838.

[63] X. M. Zheng , N. Xiao , Z. H. Long , L. J. Wang , F. X. Ye , J. H. Fang , L. J. Shen , X. W. Xiao , Synthetic Met. 2020, 263, 116365.

[64] a) D. W. Kang , M. Kang , H. Kim , J. H. Choe , D. W. Kim , J. R. Park , W. R. Lee , D. Moon , C. S. Hong , Angew. Chem., Int. Ed. 2019, 58, 16152; 10.1002/anie.201911087 31502347

[65] G. L. Xing , I. Bassanetti , S. Bracco , M. Negroni , C. Bezuidenhout , T. Ben , P. Sozzani , A. Comotti , Chem. Sci. 2019, 10, 730. 30809339 10.1039/c8sc04376k PMC6354830

[66] S. Chand , S. C. Pal , A. Pal , Y. X. Ye , Q. J. Lin , Z. J. Zhang , S. C. Xiang , M. C. Das , Chem‐Eur. J. 2019, 25, 1691. 30462360 10.1002/chem.201805177

[67] A. Garai , A. G. Kumar , S. Banerjee , K. Biradha , Chem‐Asian J 2019, 14, 4389. 31674149 10.1002/asia.201901338

[68] J. Samanta , R. W. Dorn , W. L. Zhang , X. F. Jiang , M. S. Zhang , R. J. Staples , A. J. Rossini , C. F. Ke , Chem‐Us 2022, 8, 253.

[69] K. T. Holman , A. M. Pivovar , J. A. Swift , M. D. Ward , Acc. Chem. Res. 2001, 34, 107. 11263869 10.1021/ar970272f

[70] Y. T. Li , M. Handke , Y. S. Chen , A. G. Shtukenberg , C. H. T. Hu , M. D. Ward , J. Am. Chem. Soc. 2018, 140, 12915. 30264567 10.1021/jacs.8b07065

[71] Y. Z. Liu , C. H. Hu , A. Comotti , M. D. Ward , Science 2011, 333, 436. 21778396 10.1126/science.1204369

[72] Y. Wang , X. Hou , C. Liu , M. K. Albolkany , Y. Wang , N. Wu , C. Chen , B. Liu , Nat. Commun. 2020, 11, 3124. 32561736 10.1038/s41467-020-16976-1 PMC7305155

[73] A. Yamamoto , S. Uehara , T. Hamada , M. Miyata , I. Hisaki , N. Tohnai , Cryst. Growth Des. 2012, 12, 4600.

[74] F. Q. Liu , J. W. Liu , Z. Gao , L. Wang , X. Z. Fu , L. X. Yang , Y. Tao , W. H. Yin , F. Luo , Appl. Catal B‐Environ. 2019, 258, 117973.

[75] Y. X. Lin , X. F. Jiang , S. T. Kim , S. B. Alahakoon , X. S. Hou , Z. Y. Zhang , C. M. Thompson , R. A. Smaldone , C. F. Ke , J. Am. Chem. Soc. 2017, 139, 7172. 28506061 10.1021/jacs.7b03204

[76] X. F. Jiang , X. Z. Cui , A. J. E. Duncan , L. Li , R. P. Hughes , R. J. Staples , E. V. Alexandrov , D. M. Proserpio , Y. Y. Wu , C. F. Ke , J. Am. Chem. Soc. 2019, 141, 10915. 31246447 10.1021/jacs.9b05232

[77] J. Jiang , Y. Zhao , O. M. Yaghi , J. Am. Chem. Soc. 2016, 138, 3255. 26863450 10.1021/jacs.5b10666

[78] Y. Zhang , M. Tian , Z. Majeed , Y. Xie , K. Zheng , Z. Luo , C. Li , C. Zhao , Separations 2023, 10, 196.

[79] a) B. Han , H. L. Wang , C. M. Wang , H. Wu , W. Zhou , B. L. Chen , J. Z. Jiang , J. Am. Chem. Soc. 2019, 141, 8737; 31117661 10.1021/jacs.9b03766 PMC7928070

[80] Y. H. Luo , L. Zhang , W. X. Fang , S. H. Ma , H. Dong , S. Su , Z. Y. Zheng , D. N. Li , L. H. Zhai , Chem. Commun. 2021, 57, 5901. 10.1039/d1cc01626a 34008620

[81] X. Zhang , J. X. Wang , L. B. Li , J. Y. Pei , R. Krishna , H. Wu , W. Zhou , G. D. Qian , B. L. Chen , B. Li , Angew. Chem., Int. Ed. 2021, 60, 10304. 10.1002/anie.202100342 33630416

[82] J. Y. Yang , J. K. Wang , X. N. Zhang , M. Chen , B. Q. Tian , N. Wang , X. Huang , H. X. Hao , Micropor. Mesopor. Mat. 2022, 330, 111624.

[83] D. Sarkar , P. C Rao , H. B. Aiyappa , S. Kurungot , S. Mandal , K. Ramanujam , S. Mandal , RSC Adv. 2016, 6, 37515.

[84] H. L. Wang , Z. B. Bao , H. Wu , R. B. Lin , W. Zhou , T. L. Hu , B. Li , J. C. G. Zhao , B. L. Chen , Chem. Commun. 2017, 53, 11150. 10.1039/c7cc06187k 28871296

[85] P. Li , O. Alduhaish , H. D. Arman , H. L. Wang , K. Alfooty , B. L. Chen , Cryst. Growth Des. 2014, 14, 3634.

[86] S. Nandi , D. Chakraborty , R. Vaidhyanathan , Chem. Commun. 2016, 52, 7249. 10.1039/c6cc02964g 27174692

[87] Y. J. Wang , M. H. Zhang , Q. Q. Yang , J. B. Yin , D. Liu , Y. X. Shang , Z. X. Kang , R. M. Wang , D. F. Sun , J. Z. Jiang , Chem. Commun. 2020, 56, 15529. 10.1039/d0cc05402j 33220663

[88] L. Gong , Y. Ye , Y. Liu , Y. Li , Z. Bao , S. Xiang , Z. Zhang , B. Chen , ACS Appl. Mater. Interfaces 2022, 14, 19623. 35465666 10.1021/acsami.2c04746

[89] Y. B. Xie , F. Y. Zhong , H. X. Chen , D. N. Chen , J. W. Wang , J. K. Gao , J. M. Yao , J. Solid State Chem. 2019, 277, 525.

[90] J. F. Feng , T. F. Liu , R. Cao , Angew. Chem., Int. Ed. 2020, 59, 22392. 10.1002/anie.202006926 32885555

[91] B. T. Liu , X. H. Pan , D. Y. Nie , X. J. Hu , E. P. Liu , T. F. Liu , Adv. Mater. 2020, 32, 2005912. 10.1002/adma.202005912 33124716

[92] B. Yu , L. Li , S. Liu , H. Wang , H. Liu , C. Lin , C. Liu , H. Wu , W. Zhou , X. Li , T. Wang , B. Chen , J. Jiang , Angew. Chem., Int. Ed. 2021, 60, 8983. 10.1002/anie.202016710 33496055

[93] a) B. Dunn , H. Kamath , J. M. Tarascon , Science 2011, 334, 928; 22096188 10.1126/science.1212741

[94] a) Z. Chen , K. O. Kirlikovali , K. B. Idrees , M. C. Wasson , O. K. Farha , Chem‐Us 2022, 8, 693;

[95] a) H. L. Chang , Y. W. Bai , X. Y. Song , Y. F. Duan , P. P. Sun , B. Tian , G. Shi , H. You , J. Gao , F. N. Shi , Electrochim. Acta 2019, 321, 134647;

[96] X. Liu , X. Yang , H. Wang , I. Hisaki , K. Wang , J. Jiang , J. Mater. Chem. A 2022, 10, 1808.

[97] Y. L. Wu , N. E. Horwitz , K. S. Chen , D. A. Gomez‐Gualdron , N. S. Luu , L. Ma , T. C. Wang , M. C. Hersam , J. T. Hupp , O. K. Farha , R. Q. Snurr , M. R. Wasielewski , Nat. Chem. 2017, 9, 466. 28430197 10.1038/nchem.2689

[98] W. Xiong , W. Huang , M. Zhang , P. Hu , H. Cui , Q. Zhang , Chem. Mater. 2019, 31, 8069.

[99] Y. Wu , X. Mao , M. Zhang , X. Zhao , R. Xue , S. Di , W. Huang , L. Wang , Y. Li , Y. Li , Adv. Mater. 2021, 33, 2106079. 10.1002/adma.202106079 34632649

[100] J. Sun , W. Xue , L. Zhang , L. Dai , J. Bi , F. Yao , J. Deng , P. Xiong , Y. Fu , J. Zhu , Ind. Eng. Chem. Res. 2022, 61, 6997.

[101] C. F. Guo , Y. Gao , S. Q. Li , Y. X. Wang , X. J. Yang , C. W. Zhi , H. Zhang , Y. F. Zhu , S. Q. Chen , S. L. Chou , S. X. Dou , Y. Xiao , X. P. Luo , Adv. Funct. Mater. 2024, 2314851.

[102] E. Vorobyeva , F. Lissel , M. Salanne , M. R. Lukatskaya , ACS Nano 2021, 15, 15422. 34546032 10.1021/acsnano.1c07339

[103] a) W. Zhao , J. Peng , W. Wang , B. Jin , T. Chen , S. Liu , Q. Zhao , W. Huang , Small 2019, 15, 1901351; 10.1002/smll.201901351 30957989

[104] a) A. Halder , M. Ghosh , A. Khayum M , S. Bera , M. Addicoat , H. S. Sasmal , S. Karak , S. Kurungot , R. Banerjee , J. Am. Chem. Soc. 2018, 140, 10941; 30132332 10.1021/jacs.8b06460

[105] a) Z. Luo , Y. Ouyang , H. Zhang , M. Xiao , J. Ge , Z. Jiang , J. Wang , D. Tang , X. Cao , C. Liu , W. Xing , Nat. Commun. 2018, 9, 2120; 29844358 10.1038/s41467-018-04501-4 PMC5974284

[106] L. Giri , B. Mohanty , R. Thapa , B. K. Jena , V. R. Pedireddi , ACS Omega 2022, 7, 22440. 35811884 10.1021/acsomega.2c01585 PMC9260925

[107] C. Wang , R. Furlan de Oliveira , K. Jiang , Y. Zhao , N. Turetta , C. Ma , B. Han , H. Zhang , D. Tranca , X. Zhuang , L. Chi , A. Ciesielski , P. Samorì , Nat. Commun. 2022, 13, 510. 35082288 10.1038/s41467-022-28116-y PMC8791956

[108] J. Wang , L. Zhang , S. Lin , Z. Anorg. Allg. Chem. 2022, 648, e202200125.

[109] Y. Guo , Q. Sun , Q. Huang , Y. Hu , K. Su , T. T. Li , S. Huang , J. Qian , Carbon 2022, 196, 457.

[110] Y. Zheng , S. Chen , H. Song , H. Guo , K. A. I. Zhang , C. Zhang , T. Liu , Nanoscale 2020, 12, 14441. 32614348 10.1039/d0nr04346j

[111] S. Wang , Z. Shen , Q. Wang , H. Y. Wang , Electrochim. Acta 2021, 400, 139475.

[112] R. X. Zheng , Q. L. Meng , H. Zhang , T. Li , D. Yang , L. Zhang , X. L. Jia , C. P. Liu , J. B. Zhu , X. Z. Duan , M. L. Xiao , W. Xing , J. Energy Chem. 2024, 90, 7.

[113] J. Lü , C. Perez‐Krap , M. Suyetin , N. H. Alsmail , Y. Yan , S. Yang , W. Lewis , E. Bichoutskaia , C. C. Tang , A. J. Blake , R. Cao , M. Schröder , J. Am. Chem. Soc. 2014, 136, 12828. 25184689 10.1021/ja506577g PMC4183619

[114] W. Yang , F. Yang , T. L. Hu , S. C. King , H. Wang , H. Wu , W. Zhou , J. R. Li , H. D. Arman , B. Chen , Cryst. Growth Des. 2016, 16, 5831.

[115] S. M. Chen , Y. Ju , H. Zhang , Y. B. Zou , S. Lin , Y. B. Li , S. Q. Wang , E. Ma , W. H. Deng , S. C. Xiang , B. L. Chen , Z. J. Zhang , Angew. Chem., Int. Ed. 2023, 62, e2023084. 10.1002/anie.202308418 37401627

[116] a) A. A. Zhang , Y. L. Li , Z. B. Fang , L. Xie , R. Cao , Y. Liu , T. F. Liu , ACS Appl. Mater. Interfaces 2022, 14, 21050; 35476406 10.1021/acsami.2c02917

[117] a) D. H. Nam , O. Shekhah , G. H. Lee , A. Mallick , H. Jiang , F. W. Li , B. Chen , J. Wicks , M. Eddaoudi , E. H. Sargent , J. Am. Chem. Soc. 2020, 142, 21513; 33319985 10.1021/jacs.0c10774

[118] a) S. Qiao , B. Zhang , Q. Li , Z. Li , W. Wang , J. Zhao , X. Zhang , Y. Hu , ChemSusChem 2019, 12, 5032; 31552705 10.1002/cssc.201902582

[119] H. Coskun , A. Aljabour , P. de Luna , H. Sun , N. Nishiumi , T. Yoshida , G. Koller , M. G. Ramsey , T. Greunz , D. Stifter , M. Strobel , S. Hild , A. W. Hassel , N. S. Sariciftci , E. H. Sargent , P. Stadler , Adv. Mater. 2020, 32, 1902177. 10.1002/adma.201902177 32419235

[120] F. Wang , Q. Wang , S. Wang , K. Zhang , S. Jia , J. Chen , X. Wang , ACS Nano 2022, 16, 9049. 35695291 10.1021/acsnano.2c00507

[121] S. Wang , Y. Wang , Y. F. Fu , T. F. Liu , G. X. Wang , J. Energy Chem. 2023, 87, 408.

[122] a) D. Wakerley , S. Lamaison , J. Wicks , A. Clemens , J. Feaster , D. Corral , S. A. Jaffer , A. Sarkar , M. Fontecave , E. B. Duoss , S. Baker , E. H. Sargent , T. F. Jaramillo , C. Hahn , Nat. Energy 2022, 7, 130;

[123] A. A. Zhang , D. Si , H. Huang , L. Xie , Z. B. Fang , T. F. Liu , R. Cao , Angew. Chem., Int. Ed. 2022, 61, e202203955. 10.1002/anie.202203955 35441462

[124] a) B. T. Nguyen , H. L. Nguyen , T. C. Nguyen , K. E. Cordova , H. Furukawa , Chem. Mater. 2016, 28, 6243;

[125] W. Xia , Y. Xie , S. Jia , S. Han , R. Qi , T. Chen , X. Xing , T. Yao , D. Zhou , X. Dong , J. Zhai , J. Li , J. He , D. Jiang , Y. Yamauchi , M. He , H. Wu , B. Han , J. Am. Chem. Soc. 2023, 145, 17253. 37498730 10.1021/jacs.3c04612

[126] a) J. R. Karra , K. S. Walton , J. Phys. Chem. C 2010, 114, 15735;

[127] J. X. Wang , J. Pei , X. W. Gu , Y. X. Lin , B. Li , G. Qian , Chem. Commun. 2021, 57, 10051. 10.1039/d1cc03438c 34505863

[128] M. P. Suh , H. J. Park , T. K. Prasad , D. W. Lim , Chem. Rev. 2012, 112, 782. 22191516 10.1021/cr200274s

[129] a) N. Rangnekar , N. Mittal , B. Elyassi , J. Caro , M. Tsapatsis , Chem. Soc. Rev. 2015, 44, 7128; 26155855 10.1039/c5cs00292c

[130] a) A. J. Rieth , M. Dincă , J. Am. Chem. Soc. 2018, 140, 3461; 29425040 10.1021/jacs.8b00313

[131] X. Song , Y. Wang , C. Wang , X. Gao , Y. Zhou , B. Chen , P. Li , J. Am. Chem. Soc. 2024, 146, 627. 38133431 10.1021/jacs.3c10492

[132] Y. Cai , H. Chen , P. Liu , J. Chen , H. Xu , T. Alshahrani , L. Li , B. Chen , J. Gao , Micropor. Mesopor. Mat. 2023, 352, 112495.

[133] S. Liu , Z. X. Kang , L. L. Fan , X. T. Li , B. C. Zhang , Y. Feng , H. Y. Liu , W. D. Fan , R. M. Wang , D. F. Sun , J. Membrane Sci. 2023, 678, 121674.

[134] B. Wang , R. B. Lin , Z. Zhang , S. Xiang , B. Chen , J. Am. Chem. Soc. 2020, 142, 14399. 32786796 10.1021/jacs.0c06473

[135] Z. Bao , D. Xie , G. Chang , H. Wu , L. Li , W. Zhou , H. Wang , Z. Zhang , H. Xing , Q. Yang , M. J. Zaworotko , Q. Ren , B. Chen , J. Am. Chem. Soc. 2018, 140, 4596. 29540058 10.1021/jacs.7b13706

[136] Z. Yuan , X. H. Jiang , L. J. Chen , J. J. Chen , L. Li , Y. S. Yang , Y. B. Li , F. H. Xiang , S. C. Xiang , B. L. Chen , Z. J. Zhang , CCS Chem. 2023, 6, 663.

[137] Y. Wang , S. B. Peh , D. Zhao , Small 2019, 15, 1900058. 10.1002/smll.201900058 30993886

[138] X. Zhang , L. Li , J. X. Wang , H. M. Wen , R. Krishna , H. Wu , W. Zhou , Z. N. Chen , B. Li , G. Qian , B. Chen , J. Am. Chem. Soc. 2020, 142, 633. 31838841 10.1021/jacs.9b12428 PMC11061857

[139] P. Q. Liao , W. X. Zhang , J. P. Zhang , X. M. Chen , Nat. Commun. 2015, 6, 8697. 26510376 10.1038/ncomms9697 PMC4846320

[140] R. B. Lin , H. Wu , L. Li , X. L. Tang , Z. Li , J. Gao , H. Cui , W. Zhou , B. Chen , J. Am. Chem. Soc. 2018, 140, 12940. 30216725 10.1021/jacs.8b07563

[141] Y. Chen , Z. Qiao , H. Wu , D. Lv , R. Shi , Q. Xia , J. Zhou , Z. Li , Chem. Eng. Sci. 2018, 175, 110.

[142] Y. Chen , Y. Yang , Y. Wang , Q. Xiong , J. Yang , S. Xiang , L. Li , J. Li , Z. Zhang , B. Chen , J. Am. Chem. Soc. 2022, 144, 17033. 36069372 10.1021/jacs.2c06585

[143] Y. L. Cai , J. K. Gao , J. H. Li , P. X. Liu , Y. C. Zheng , W. Zhou , H. Wu , L. B. Li , R. B. Lin , B. L. Chen , Angew. Chem., Int. Ed. 2023, 62, e202308579. 10.1002/anie.202308579 37486880

[144] Q. Yin , K. Pang , Y. N. Feng , L. L. Han , A. Morsali , X. Y. Li , T. F. Liu , Nat. Commun. 2024, 15, 634. 38245504 10.1038/s41467-024-44921-z PMC10799873

[145] Y. Feng , Y. Xu , S. Liu , D. Wu , Z. Su , G. Chen , J. Liu , G. Li , Coordin. Chem. Rev. 2022, 459, 214414.

[146] Z. Zhang , Y. Ye , S. Xiang , B. Chen , Acc. Chem. Res. 2022, 55, 3752. 36454588 10.1021/acs.accounts.2c00686

[147] X. Qin , Z. Zhan , Z. Ding , Curr. Opin. Electroche. 2023, 39, 101283.

[148] J. Mei , N. L. C. Leung , R. T. K. Kwok , J. W. Y. Lam , B. Z. Tang , Chem. Rev. 2015, 115, 11718. 26492387 10.1021/acs.chemrev.5b00263

[149] Q. Huang , W. Li , Z. Yang , J. Zhao , Y. Li , Z. Mao , Z. Yang , S. Liu , Y. Zhang , Z. Chi , CCS Chem 2022, 4, 1643.

[150] Y. Lv , D. Li , A. Ren , Z. Xiong , Y. Yao , K. Cai , S. Xiang , Z. Zhang , Y. S. Zhao , ACS Appl. Mater. Interfaces 2021, 13, 28662. 34114811 10.1021/acsami.1c06312

[151] H. Zhou , Q. Ye , X. Wu , J. Song , C. M. Cho , Y. Zong , B. Z. Tang , T. S. A. Hor , E. K. L. Yeow , J. Xu , J. Mater. Chem. C 2015, 3, 11874.

[152] Y. X. Lin , C. H. Jiang , Y. B. Wang , J. X. Wang , B. Li , G. D. Qian , J. Mater. Chem. A 2023, 12, 153.

[153] C. Wang , Y. Wang , K. O. Kirlikovali , K. Ma , Y. Zhou , P. Li , O. K. Farha , Adv. Mater. 2022, 34, 2202287. 10.1002/adma.202202287 35790037

[154] Q. Y. Huang , K. I. Otake , S. Kitagawa , Angew. Chem., Int. Ed. 2023, 62, e2023102. 10.1002/anie.202310225 37596804

[155] Y. F. Zhao , H. Zeng , X. W. Zhu , W. G. Lu , D. Li , Chem. Soc. Rev. 2021, 50, 4484. 33595006 10.1039/d0cs00955e

[156] S. Xu , Q. Zhang , Mater. Today Energy 2021, 20, 100635.

[157] a) J. A. Swift , A. M. Reynolds , M. D. Ward , Chem. Mater. 1998, 10, 4159;

[158] a) S. Lee , E. A. Kapustin , O. M. Yaghi , Science 2016, 353, 808; 27540171 10.1126/science.aaf9135

[159] Y. Li , S. Tang , A. Yusov , J. Rose , A. N. Borrfors , C. T. Hu , M. D. Ward , Nat. Commun. 2019, 10, 4477. 31578331 10.1038/s41467-019-12453-6 PMC6775153

[160] a) J. Yang , Y. W. Yang , Small 2020, 16, 1906846;

[161] R. Domínguez‐González , I. Rojas‐León , E. Martínez‐Ahumada , D. Martínez‐Otero , H. A. Lara‐García , J. Balmaseda‐Era , I. A. Ibarra , E. G. Percástegui , V. Jancik , J. Mater. Chem. A 2019, 7, 26812.

[162] Y. Liu , J. Dai , L. Guo , Z. Zhang , Y. Yang , Q. Yang , Q. Ren , Z. Bao , CCS Chem 2022, 4, 381.

[163] Y. Liu , H. Wu , L. Guo , W. Zhou , Z. Zhang , Q. Yang , Y. Yang , Q. Ren , Z. Bao , Angew. Chem., Int. Ed. 2022, 61, e202117609. 10.1002/anie.202117609 34989467

[164] J. L. Huang , Y. B. Li , H. Zhang , Z. Yuan , S. C. Xiang , B. L. Chen , Z. J. Zhang , Angew. Chem., Int. Ed. 2023, 62, e2023159.

[165] Z. Yuan , L. J. Chen , X. Zhou , L. Li , Y. B. Li , Y. S. Yang , Z. Q. Zhou , Y. T. Chen , S. C. Xiang , B. L. Chen , Z. J. Zhang , J. Mater. Chem. A 2023, 11, 21857.

[166] a) P. Singare , R. Lokhande , Nat. Sci. 2009, 1, 191.

[167] a) Z. Zhang , X. Dong , J. Yin , Z. G. Li , X. Li , D. Zhang , T. Pan , Q. Lei , X. Liu , Y. Xie , F. Shui , J. Li , M. Yi , J. Yuan , Z. You , L. Zhang , J. Chang , H. Zhang , W. Li , Q. Fang , B. Li , X. H. Bu , Y. Han , J. Am. Chem. Soc. 2022, 144, 6821; 35380829 10.1021/jacs.2c00563

[168] M. Zhang , J. Samanta , B. A. Atterberry , R. Staples , A. J. Rossini , C. Ke , Angew. Chem., Int. Ed. 2022, 61, e202214189. 10.1002/anie.202214189 36331335

[169] B. Li , W. G. Qiu , G. P. A. Yap , Y. L. Dory , J. P. Claverie , Adv. Funct. Mater. 2023, 34, 2311964.

[170] I. Saptiama , Y. V. Kaneti , H. Oveisi , Y. Suzuki , K. Tsuchiya , K. Takai , T. Sakae , S. Pradhan , M. S. A. Hossain , N. Fukumitsu , K. Ariga , Y. Yamauchi , B. Chem. Soc. JPN. 2018, 91, 195.

[171] A. Kaushik , K. Marvaniya , Y. Kulkarni , D. Bhatt , J. Bhatt , M. Mane , E. Suresh , S. Tothadi , K. Patel , S. Kushwaha , Chem‐Us 2022, 8, 2749.

[172] A. Maurya , K. Marvaniya , P. Dobariya , M. V. Mane , S. Tothadi , K. Patel , S. Kushwaha , Small 2023, 20, 2306824. 10.1002/smll.202306824 37975153

[173] P. Wu , X. Y. Yin , Y. F. Zhao , F. Z. Li , Y. Y. Yang , N. Liu , J. L. Liao , T. Lan , J. Hazard. Mater. 2023, 459, 132179. 37531757 10.1016/j.jhazmat.2023.132179

[174] H. T. Chen , H. Q. Huang , H. Y. Xu , T. Wu , Y. B. Xu , X. M. Ma , W. Yi , G. S. Chen , S. M. Huang , G. F. Ouyang , Small 2023, 2308716.

[175] M. K. Ghosh , S. Giri , T. K. Ghorai , J. Mol. Struct. 2020, 1206, 127727.

[176] a) K. Jedynak , D. Wideł , N. Rędzia , Colloids Interfaces 2019, 3, 30;

[177] X. T. Jiang , Q. Yin , B. T. Liu , J. Y. Chen , R. Wang , T. F. Liu , Nanoscale Adv 2021, 3, 3441. 36133715 10.1039/d1na00199j PMC9419181

[178] X. X. Zhang , W. Liu , F. Han , L. S. Jiang , Z. Y. Li , Appl. Surf. Sci. 2024, 644, 158770.

[179] C. H. Zeng , Z. Luo , J. Yao , CrystEngComm 2017, 19, 613.

[180] Y. Lv , X. Qin , K. Hu , F. Ye , S. Zhao , Sensor. Actuat. B: Chem. 2022, 353, 131132.

[181] Y. L. Hu , Y. Y. Li , Y. R. Shi , Y. X. Kuang , S. X. Zhou , Y. Peng , Y. F. Liu , L. Y. Chen , N. B. Zhou , J. Zheng , F. Zhu , G. F. Ouyang , Food Chem. 2023, 415, 135790. 36868067 10.1016/j.foodchem.2023.135790

[182] a) H. Shi , D. Feng , H. Li , D. Yu , X. Chen , J. Photoch. Photobio. A. 2023, 435, 114292;

[183] Y. Liu , L. Chen , L. Yang , T. Lan , H. Wang , C. Hu , X. Han , Q. Liu , J. Chen , Z. Feng , X. Cui , Q. Fang , H. Wang , L. Li , Y. Li , H. Xing , S. Yang , D. Zhao , J. Li , Green Energy Environ. 2024, 9, 217.

[184] W. P. Li , J. F. Shi , Y. Chen , X. Y. Liu , X. X. Meng , Z. Y. Guo , S. H. Li , B. Y. Zhang , Z. Y. Jiang , Chem. Eng. J. 2023, 468, 143609.

[185] Z. Tang , X. Li , L. Tong , H. Yang , J. Wu , X. Zhang , T. Song , S. Huang , F. Zhu , G. Chen , G. Ouyang , Angew. Chem., Int. Ed. 2021, 60, 23608. 10.1002/anie.202110351 34459532

[186] G. Chen , L. Tong , S. Huang , S. Huang , F. Zhu , G. Ouyang , Nat. Commun. 2022, 13, 4816. 35974100 10.1038/s41467-022-32454-2 PMC9381776

[187] G. Chen , S. Huang , X. Ma , R. He , G. Ouyang , Nat. Protoc. 2023, 18, 2032. 37198321 10.1038/s41596-023-00828-5

[188] H. S. Yang , J. H. Fu , W. Huang , T. Wu , S. M. Huang , G. S. Chen , G. F. Ouyang , Small Struct 2023, 4, 2200346.

[189] R. B. Lin , B. Chen , Nat. Chem. 2019, 11, 1078. 31723258 10.1038/s41557-019-0382-y

[190] a) H. Zhang , D. Yu , S. Liu , C. Liu , Z. Liu , J. Ren , X. Qu , Angew. Chem., Int. Ed. 2022, 61, e202109068; 10.1002/anie.202109068 34735035

[191] C. Huang , C. Zhao , Q. Deng , H. Zhang , D. Yu , J. Ren , X. Qu , Nat. Catal. 2023, 6, 729.

[192] J. K. Tang , J. Liu , Q. Z. Zheng , R. Yao , M. Wang , Angew. Chem., Int. Ed. 2023, 62, e2023127. 10.1002/anie.202312784 37817650

[193] N. Yin , Y. H. Wang , Y. Liu , R. Niu , S. Zhang , Y. Cao , Z. J. Lv , S. Y. Song , X. G. Liu , H. J. Zhang , Adv. Mater. 2023, 35, 2303567. 10.1002/adma.202303567 37466394

[194] B. T. Liu , X. H. Pan , D. Y. Zhang , R. Wang , J. Y. Chen , H. R. Fang , T. F. Liu , Angew. Chem., Int. Ed. 2021, 60, 25701. 10.1002/anie.202110028 34477299

[195] Q. Ye , S. H. Chen , Y. Zhang , B. Ruan , Y. J. Zhang , X. K. Zhang , T. Jiang , X. Wang , N. Ma , F. C. Tsai , Macromol. Biosci. 2021, 21, 2100317. 10.1002/mabi.202100317 34626523

[196] Y. Wang , R. Cao , C. Wang , X. Y. Song , R. N. Wang , J. C. Liu , M. M. Zhang , J. Y. Huang , T. T. You , Y. H. Zhang , D. P. Yan , W. D. Han , L. Yan , J. S. Xiao , P. Li , Adv. Funct. Mater. 2023, 33, 2214388.

[197] J. Xiao , A. R. M. Shaheer , C. Liu , T. F. Liu , R. Cao , Aggregate 2024, 5, e481.

[198] Q. Zhou , Y. Guo , Y. Zhu , Nat. Catal. 2023, 6, 574.

[199] Z. Cheng , Y. Fang , Y. Yang , H. Zhang , Z. Fan , J. Zhang , S. Xiang , B. Chen , Z. Zhang , Angew. Chem., Int. Ed. 2023, 62, e202311480. 10.1002/anie.202311480 37725404

[200] L. J. Yang , J. W. Yuan , G. Wang , Q. Cao , C. Zhang , M. M. Li , J. X. Shao , Y. Xu , H. Li , J. M. Lu , Adv. Funct. Mater. 2023, 33, 2300954.

[201] X. Zhao , Q. Yin , X. Mao , C. Cheng , L. Zhang , L. Wang , T. F. Liu , Y. Li , Y. Li , Nat. Commun. 2022, 13, 2721. 35581214 10.1038/s41467-022-30523-0 PMC9114359

[202] Z. J. Lin , R. Cao , Acta Chim. Sinica 2020, 78, 1309.

[203] W. K. Qin , D. H. Si , Q. Yin , X. Y. Gao , Q. Q. Huang , Y. N. Feng , L. Xie , S. Zhang , X. S. Huang , T. F. Liu , R. Cao , Angew. Chem., Int. Ed. 2022, 61, e202202089. 10.1002/anie.202202089 35460153

[204] I. Hisaki , C. Xin , K. Takahashi , T. Nakamura , Angew. Chem., Int. Ed. 2019, 58, 11160. 10.1002/anie.201902147 30891889

[205] Z. H. Zhu , H. L. Wang , H. H. Zou , F. P. Liang , Dalton T 2020, 49, 10708. 10.1039/d0dt01998d 32672293

[206] C. C. Fang , X. F. Xu , X. Y. Zhang , L. M. Dai , F. L. Yao , W. Y. Zhang , Y. S. Fu , J. W. Sun , J. W. Zhu , Chin. Sci. B‐Chin. 2023, 68, 3335.

[207] Z. W. Yang , Y. F. Zhang , W. J. Wu , Z. F. Zhou , H. X. Gao , J. T. Wang , Z. Y. Jiang , J. Membrane Sci. 2022, 664, 121118.

[208] X. Y. Gao , W. Y. Lu , Y. Wang , X. Y. Song , C. Wang , K. O. Kirlikovali , P. Li , Sci. China Chem. 2022, 65, 2077.

[209] W. K. Qin , C. H. Tung , L. Z. Wu , J. Mater. Chem. A 2023, 11, 12521.

