# Supplementary material for: Structural Design and Energy and Environmental Applications of Hydrogen‐Bonded Organic Frameworks: A Systematic Review
Source: Adv Sci (Weinh). 2024 Apr 22;11(22):2400101. doi: 10.1002/advs.202400101 (PMC11165539; doi:10.1002/advs.202400101)
Supplement: Supplementary file 1 — Supporting Information [file ADVS-11-2400101-s001.pdf]

ADVANCED SCIENCE
Open Access

Supporting Information

for *Adv. Sci.*, DOI 10.1002/advs.202400101

Structural Design and Energy and Environmental Applications of Hydrogen-Bonded Organic Frameworks: A Systematic Review

Xiaoming Liu, Guangli Liu, Tao Fu, Keren Ding, Jinrui Guo, Zhenran Wang, Wei Xia and Huayuan Shangguan**

Structural Design and Energy & Environmental Applications of Hydrogen-Bonded Organic Frameworks: A Systematic Review

Xiaoming Liu [a,#], Guangli Liu [b,#], Tao Fu [b,#,*], Keren Ding [c], Jinrui Guo [d], Zhenran Wang [e], Wei Xia [a], Huayuan Shangguan [f,*]

[a] *Department of resources and environment, Moutai Institute, Renhuai 564507, China*
[b] *College of Environmental Sciences and Engineering, Peking University, Beijing 100871, China*
[c] *AgResearch, Ruakura Research Centre, Hamilton 3240, New Zealand*
[d] *College of Environmental Science and Engineering, Tongji University, Shanghai 200092, China*
[e] *School of Environmental Science and Engineering, Southwest Jiaotong University, Chengdu 611756, China*
[f] *Key Laboratory of Urban Environment and Health, Institute of Urban Environment, Chinese Academy of Sciences, Xiamen 361021, China*

[#]*These authors contributed equally to this work.*
[*]*Co-corresponding authors.*
Tao Fu, taofuky@stu.pku.edu.cn, College of Environmental Sciences and Engineering, Peking University, Beijing 100871, China
Huayuan Shangguan, hyshangguan@iue.ac.cn, Institute of Urban Environment, Chinese Academy of Sciences, Xiamen 361021, China.

Table S1 Summary of porosity and functionality in porous HOFs.

H-Bonding motifs	HOFs	Pore size/Å	Pore volume / cm^3g^{-1}	SBET/m^2g^{-1}	Applications	References
Carboxylate	HOF-11	6.2 × 6.8	0.35	687	C_2H_2/CH_4, CO_2/CH_4, CO_2/N_2 separation	[1]
	HOF-12	8.6 × 10.8	0.63	320	CO_2/CH_4 separation	[2]
	TCF-1	5.5 × 2.9	-	96.8	CO_2, CH_4, and Xe adsorption	[3]
	HOF-100	8 × 12	0.36	900	Mustard gas detoxification	[4]
	HOF-14	31.2 × 24.1	1.36	2573	C3/CH_4, C2/CH_4 separation, mustard gas detoxification	[4]
	PFC-1	18 × 23	0.95	2122	Photodynamic therapy	[5]
	PFC-2	29.7,10.7	0.64	1014	C2/CH_4 separation	[6]
	HOF-TCBP	17.8 × 26.3	0.83	2066	C4/CH_4, C3/CH_4 separation	[7]
	ECUT-HOF-30	4.0 × 4.1	-	402	C_2H_2/CO_2 separation	[8]
	HOF-76	7.0	0.4	1121	C_2H_6/C_2H_4 separation	[9]
	PETHOF-1	16	-	1150	-	[10]
	PETHOF-2	11	-	1140	-	[10]
	HOF-C8	5.6 × 9.8	0.354	465	Crystal jumping behavior	[11]
	CPHATN-1	8.8	-	379	Acid-induced color change	[12]
	CPBTQ-1	5.0	0.37	471	Acid-induced color change	[13]
	Tp-apo	8.5	-	718	Vapor sorption of hydrocarbons	[14]
	TpMe-apo	7.7	-	561	Fluorescence	[15]
	CPHAT-1	6.4	-	649	Iodine adsorption	[16]
	CBPHAT-1	14.5	-	1288	Anisotropic fluorescence emission, acid-induced color	[17]

					change	
	CoTCPp	3.98 × 6.47	0.052	98	Catalysis	[18]
	PFC-5	5.2 × 4.0	-	256	C_2/CH_4 separation	[19]
	PFC-11	-	0.38	751	Vapor adsorption	[20]
	HOF-BTB	18.5	0.42	1095	CO_2, N_2, CH_4 adsorption, C_2/CH_4 separation, Xe/Kr separation	[21]
	ABTPA-2	18.0	-	1183	CH_4 adsorption, negative thermal expansion	[22]
	CBPE-1	6.8	0.20	555	Fluorescence	[23]
	HOF-20	13.0	0.57	1323	Aniline sensing	[24]
Diaminotriazine (DAT)	HOF-1	8.2	-	359	C_2H_2/C_2H_4 separation	[25]
	HOF-2	4.8	0.13	238	Enantioselective separation of secondary alcohols	[26]
	HOF-3	7	-	165	C_2H_2/CO_2 separation	[27]
	HOF-4	3.8 × 8.1	-	312	C_2H_4/C_2H_6 separation	[28]
	HOF-5	3.9 × 5.5	0.44	1101	C_2H_2/CH_4, CO_2/CH_4, CO_2/N_2 separation	[29]
	HOF-6	~6.4, ~7.5	-	130	CO_2/N_2 separation and proton conduction	[30]
	HOF-7	3.2 × 4.7 ,4.2 × 6.7	-	124	CO_2/N_2 separation	[31]
	HOF-9	6.9 × 8.8	0.17	286	Selective recognition toward Py over BTX	[32]
	HOF-10	12.8 × 26.0	-	187	Fluorescence sensing of Ag^+ ions	[33]
	UPC-HOF-6	~2.8	-	237	HOF films for H_2/N_2 separation	[34]
	HOF-D8	6.3×13	-	515	-	[35]
Urea	TTBI	7.8	1.02	2796		[36]
	T2-γ	19.9	1.57	3425	CH_4 storage	[37]

	T2-β	-	-	1665	-	[37]
	T2-δ	-	-	365	H_2 storage	[37]
	T2E-α	-	-	3599	-	[37]
Imidazole	FDM-15	11.5	-	749	C60 adsorption and separation of aromatic compounds	[38]
Pyrazole	trispyrazole 1	16.5	-	1159	Fluorocarbons and CFCs removal	[39]
	trispyrazole 16	15.8	-	903	Aggregation-induced emission	[40]
	trispyrazole 23	11.9	-	221	-	[40]
	trispyrazole 25	26.4	-	1821	-	[40]
Pyridine	HOF-8	6.8 × 4.5	-	-	CO_2 and benzene adsorption	[41]
	SOF-1	7.4	0.23	474	Gas storage	[42]
	SOF-7	13.5 × 14.0	0.233	900	CO_2 adsorption	[43]
	SOF-9	12.1 × 14.2	-	182	CO_2 adsorption	[44]
	SOF-10	6.0	-	221	CO_2 adsorption	[44]
	Py open	6.0	-	219	-	[45]
	JLUE-SOF-3-DMSO	-	-	81	Iodine adsorption	[46]
	TCPP-1,3-DPP	-	-	258	Combined chemical-photodynamic therapy	[47]
Cyanide	HOF-FJU-1	6.0 , 11.4	0.15	385	C_2H_2/C_2H_4 separation	[48]
	HOF-FJU-4	-	-	-	Micro-lasers	[49]
	HOF-FJU-5	-	-	-	Micro-lasers	[49]
	HOF-40	4.15 × 3.85	0.12	234	Superior separation for Xe/Kr mixtures with excellent chemical stability	[50]
	ZJU-HOF-25	-	-	-	Construction of organic fluorophores with	[51]

					high quantum efficiency	
	HOF-26,HOF-27,HOF-28	13.3×6.5, 12.1×11.0,11.3×5.2	-	-	-	[52]
Sulfonic acid	HOF-GS-10	-	-	-	Proton-conducting	[53]
	HOF-GS-11	-	-	-	Proton-conducting	[53]
	KUF-1	-	-	-	IV NH_3 adsorption	[54]
	HOF-ZJU-101	3.6	-	339	CO_2/N_2, CO_2/CH_4, C_2H_2/C_2H_4 separation	[55]
	HOF-ZJU-102	3.5	-	262	C_2H_2/C_2H_4 separation	[56]
	HOF-ZJU-103	3.0-7.0	-	389	Xe/Kr separation	[57]
	HOF-ZJU-104	2.8-7.0	-	422	Xe/Kr separation	[57]
	HOF-ZJU-201	4.8-5.8	-	423	Xe/Kr, C_3H_8/CH_4,C_2H_6/CH_4, CH_4/N_2 separation	[58]
	HOF-ZJU-202	4.9-5.9	-	366	Xe/Kr, C_3H_8/CH_4, C_2H_6/CH_4, CH_4/N_2 separation	[58]
	PFC-58				CO_2 Photoreduction	[59]
	BioHOF-1	6.4	-	-	Enzyme encapsulation	[60]
	1-Co	2.1 × 2.1 × 3.2	-	1192	Asymmetric catalysis	[61]
	1-Ni	2.1 × 2.1 × 3.2	-	1239	Asymmetric catalysis	[61]
	HOF-19	8.0 × 13.6	0.45	685	Heterogeneous catalysis	[62]
	Gd-B	~4.85	-	257	MeOH adsorption	[63]
	SPEB	4.6 × 6.2	-	50	Molecular rotor	[64]
	d-POS-1	9.1 × 10.4	-	398	Guest-responsive fluorescence	[65]
	CPOS-1-4	-	-	12-216	Proton conduction	[66]

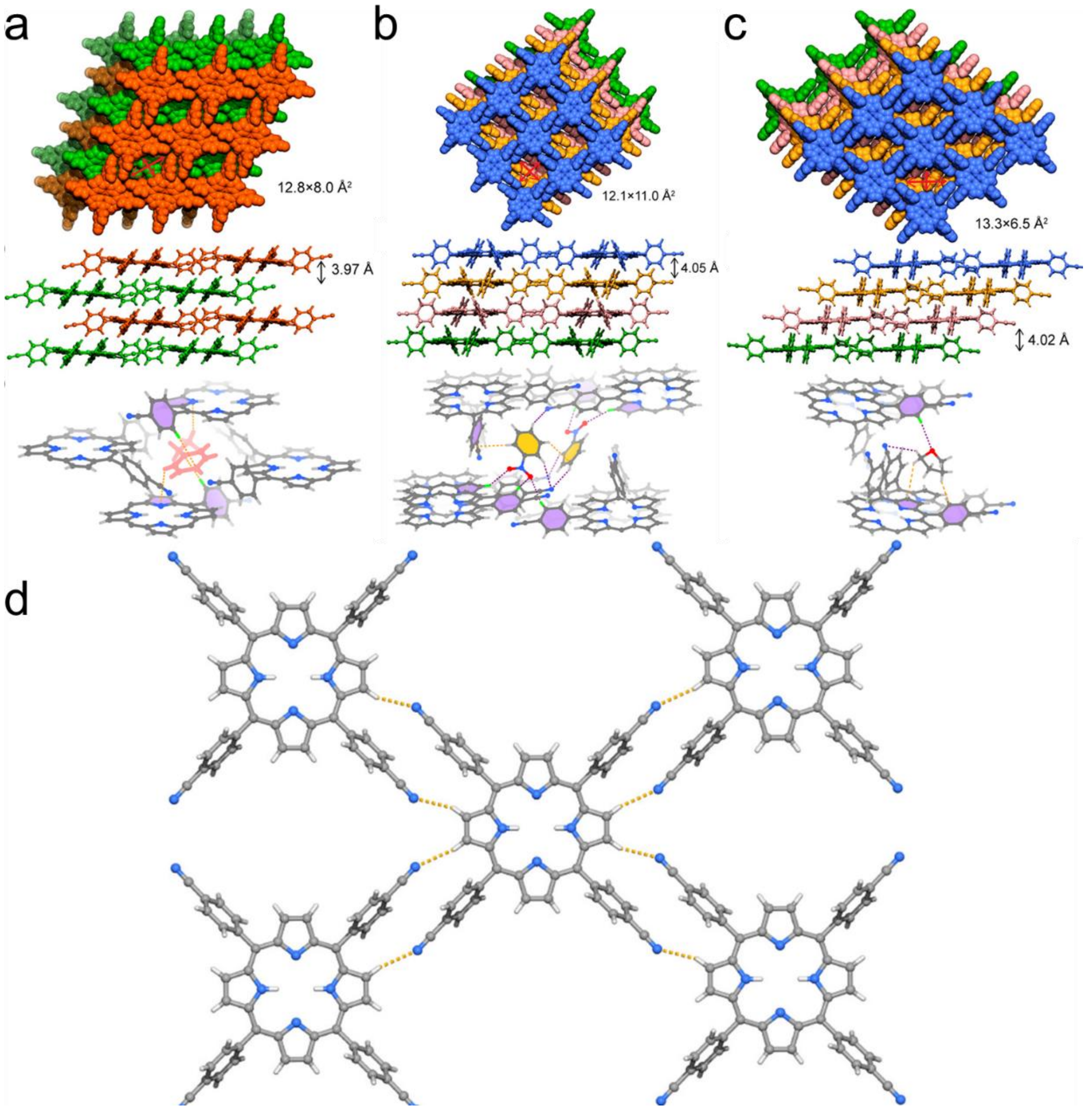

Figure S1. (a-c) Crystal structures of HOF-26/27/28, C–H···O/N, C–H···π interactions, and hydrogen-bonding donors from PTTBN are highlighted in purple, orange, and light green, respectively. Reproduced with permission.[52] Copyright 2020, American Chemical Society. (d) Structural unit in HOF-29 extended to the sql net through intermolecular C-N···H hydrogen bonds (highlighted in yellow). Reproduced with permission.[67] Copyright 2022, Wiley-VCH.

References

[1] W. Yang, J. Wang, H. Wang, Z. Bao, J. C.-G. Zhao, B. Chen, *Cryst. Growth Des.* **2017**, 17, 6132.

[2] W. Yang, W. Zhou, B. L. Chen, *Cryst. Growth Des.* **2019**, 19, 5184.

[3] I. Bassanetti, S. Bracco, A. Comotti, M. Negroni, C. Bezuidenhout, S. Canossa, P. P. Mazzeo, L. Marchió, P. Sozzani, *J. Mater. Chem. A* **2018**, 6, 14231.

[4] K. Ma, P. Li, J. H. Xin, Y. Chen, Z. Chen, S. Goswami, X. Liu, S. Kato, H. Chen, X. Zhang, J. Bai, M. C. Wasson, R. R. Maldonado, R. Q. Snurr, O. K. Farha, *Cell Rep. Phys. Sci.* **2020**, 1, 100024.

[5] Q. Yin, P. Zhao, R. J. Sa, G. C. Chen, J. Lü, T. F. Liu, R. Cao, *Angew. Chem. Int. Edit.* **2018**, 57, 7691.
[6] Q. Yin, Y. L. Li, L. Li, J. Lü, T. F. Liu, R. Cao, *ACS Appl. Mater. Inter.* **2019**, 11, 17823.
[7] F. Hu, C. Liu, M. Wu, J. Pang, F. Jiang, D. Yuan, M. Hong, *Angew. Chem. Int. Edit.* **2017**, 56, 2101.
[8] L. Wang, L. Yang, L. Gong, R. Krishna, Z. Gao, Y. Tao, W. Yin, Z. Xu, F. Luo, *Chem. Eng. J.* **2020**, 383, 123117.
[9] X. Zhang, L. B. Li, J. X. Wang, H. M. Wen, R. Krishna, H. Wu, W. Zhou, Z. N. Chen, B. Li, G. D. Qian, B. L. Chen, *J. Am. Chem. Soc.* **2020**, 142, 633.
[10] J. H. Tang, X. Li, W. Q. Zhao, Y. J. Wang, P. Cui, R. J. Zeng, L. P. Yu, S. G. Zhou, *Bioresour. Technol.* **2019**, 279, 234.
[11] T. Takeda, M. Ozawa, T. Akutagawa, *Angew. Chem. Int. Edit.* **2019**, 58, 10345.
[12] I. Hisaki, Y. Suzuki, E. Gomez, Q. Ji, N. Tohnai, T. Nakamura, A. Douhal, *J. Am. Chem. Soc.* **2019**, 141, 2111.
[13] I. Hisaki, Q. Ji, K. Takahashi, N. Tohnai, T. Nakamura, *Cryst. Growth Des.* **2020**, 20, 3190.
[14] I. Hisaki, S. Nakagawa, N. Ikenaka, Y. Imamura, M. Katouda, M. Tashiro, H. Tsuchida, T. Ogoshi, H. Sato, N. Tohnai, M. Miyata, *J. Am. Chem. Soc.* **2016**, 138, 6617.
[15] I. Hisaki, N. Ikenaka, S. Tsuzuki, N. Tohnai, *Mater. Chem. Front.* **2018**, 2, 338.
[16] I. Hisaki, N. Ikenaka, E. Gomez, B. Cohen, N. Tohnai, A. Douhal, *Chem-Eur. J.* **2017**, 23, 11611.
[17] a)I. Hisaki, Y. Suzuki, E. Gomez, B. Cohen, N. Tohnai, A. Douhal, *Angew. Chem. Int. Edit.* **2018**, 57, 12650; b)E. Gomez, Y. Suzuki, I. Hisaki, M. Moreno, A. Douhal, *J. Mater. Chem. C* **2019**, 7, 10818.
[18] Z. Q. Zhang, J. Li, Y. H. Yao, S. Sun, *Cryst. Growth Des.* **2015**, 15, 5028.
[19] Q. Yin, J. Lü, H. F. Li, T. F. Liu, R. Cao, *Cryst. Growth Des.* **2019**, 19, 4157.
[20] Y. L. Li, E. V. Alexandrov, Q. Yin, L. Li, Z. B. Fang, W. B. Yuan, D. M. Proserpio, T. F. Liu, *J. Am. Chem. Soc.* **2020**, 142, 7218.
[21] a)C. A. Zentner, H. W. H. Lai, J. T. Greenfield, R. A. Wiscons, M. Zeller, C. F. Campana, O. Talu, S. A. FitzGerald, J. L. C. Rowsell, *Chem. Commun.* **2015**, 51, 11642; b)T. U. Yoon, S. B. Baek, D. Kim, E. J. Kim, W. G. Lee, B. K. Singh, M. S. Lah, Y. S. Bae, K. S. Kim, *Chem. Commun.* **2018**, 54, 9360; c)W. G. Lee, T. U. Yoon, Y. S. Bae, K. S. Kim, S. B. Baek, *RSC Adv.* **2019**, 9, 36808.
[22] P. Cui, E. S. Grape, P. R. Spackman, Y. Wu, R. Clowes, G. M. Day, A. K. Inge, M. A. Little, A. I. Cooper, *J. Am. Chem. Soc.* **2020**, 142, 12743.
[23] Y. Suzuki, N. Tohnai, I. Hisaki, *Chem-Eur. J.* **2020**, 26, 17056.
[24] B. Wang, R. He, L. H. Xie, Z. J. Lin, X. Zhang, J. Wang, H. L. Huang, Z. J. Zhang, K. S. Schanze, J. Zhang, S. C. Xiang, B. L. Chen, *J. Am. Chem. Soc.* **2020**, 142, 12478.
[25] Y. B. He, S. C. Xiang, B. L. Chen, *J. Am. Chem. Soc.* **2011**, 133, 14570.
[26] P. Li, Y. B. He, J. Guang, L. H. Weng, J. C. G. Zhao, S. C. Xiang, B. L. Chen, *J. Am. Chem. Soc.* **2014**, 136, 547.
[27] P. Li, Y. B. He, Y. F. Zhao, L. H. Weng, H. L. Wang, R. Krishna, H. Wu, W. Zhou, M. O'Keeffe, Y. Han, B. L. Chen, *Angew. Chem. Int. Edit.* **2015**, 54, 574.
[28] P. Li, Y. B. He, H. D. Arman, R. Krishna, H. L. Wang, L. H. Weng, B. L. Chen, *Chem. Commun.* **2014**, 50, 13081.
[29] H. L. Wang, B. Li, H. Wu, T. L. Hu, Z. Z. Yao, W. Zhou, S. C. Xiang, B. L. Chen, *J. Am. Chem. Soc.* **2015**, 137, 9963.
[30] W. Yang, F. Yang, T. L. Hu, S. C. King, H. L. Wang, H. Wu, W. Zhou, J. R. Li, H. D. Arman, B. L. Chen, *Cryst. Growth Des.* **2016**, 16, 5831.

[31] W. Yang, B. Li, H. L. Wang, O. Alduhaish, K. Alfooty, M. A. Zayed, P. Li, H. D. Arman, B. L. Chen, *Cryst. Growth Des.* **2015**, 15, 2000.
[32] Y. J. Wang, D. Liu, J. B. Yin, Y. X. Shang, J. Du, Z. X. Kang, R. M. Wang, Y. L. Chen, D. F. Sun, J. Z. Jiang, *Chem. Commun.* **2020**, 56, 703.
[33] H. L. Wang, H. Wu, J. L. Kan, G. G. Chang, Z. Z. Yao, B. Li, W. Zhou, S. C. Xiang, J. C. G. Zhao, B. L. Chen, *J. Mater. Chem. A* **2017**, 5, 8292.
[34] S. Feng, Y. X. Shang, Z. K. Wang, Z. X. Kang, R. M. Wang, J. Z. Jiang, L. L. Fan, W. D. Fan, Z. N. Liu, G. D. Kong, Y. Feng, S. Q. Hu, H. L. Guo, D. F. Sun, *Angew. Chem. Int. Edit.* **2020**, 59, 3840.
[35] T. Khadivjam, H. Che-Quang, T. Maris, Z. Ajoyan, A. J. Howarth, J. D. Wuest, *Chem-Eur. J.* **2020**, 26, 7026.
[36] M. Mastalerz, I. M. Oppel, *Angew. Chem. Int. Edit.* **2012**, 51, 5252.
[37] A. Pulido, L. J. Chen, T. Kaczorowski, D. Holden, M. A. Little, S. Y. Chong, B. J. Slater, D. P. McMahon, B. Bonillo, C. J. Stackhouse, A. Stephenson, C. M. Kane, R. Clowes, T. Hasell, A. I. Cooper, G. M. Day, *Nature* **2017**, 543, 657.
[38] W. Q. Yan, X. P. Yu, T. Yan, D. F. Wu, E. L. Ning, Y. Qi, Y. F. Han, Q. W. Li, *Chem. Commun.* **2017**, 53, 3677.
[39] T. H. Chen, I. Popov, W. Kaveevivitchai, Y. C. Chuang, Y. S. Chen, O. Daugulis, A. J. Jacobson, O. S. Miljanic, *Nat. Commun.* **2014**, 5, 5131.
[40] M. I. Hashim, H. T. M. Le, T. H. Chen, Y. S. Chen, O. Daugulis, C. W. Hsu, A. J. Jacobson, W. Kaveevivitchai, X. Liang, T. Makarenko, O. S. Miljanic, I. Popovs, H. V. Tran, X. Q. Wang, C. H. Wu, J. I. Wu, *J. Am. Chem. Soc.* **2018**, 140, 6014.
[41] X. Z. Luo, X. J. Jia, J. H. Deng, J. L. Zhong, H. J. Liu, K. J. Wang, D. C. Zhong, *J. Am. Chem. Soc.* **2013**, 135, 11684.
[42] W. B. Yang, A. Greenaway, X. A. Lin, R. Matsuda, A. J. Blake, C. Wilson, W. Lewis, P. Hubberstey, S. Kitagawa, N. R. Champness, M. Schröder, *J. Am. Chem. Soc.* **2010**, 132, 14457.
[43] J. Lü, C. Perez-Krap, M. Suyetin, N. H. Alsmail, Y. Yan, S. H. Yang, W. Lewis, E. Bichoutskaia, C. C. Tang, A. J. Blake, R. Cao, M. Schröder, *J. Am. Chem. Soc.* **2014**, 136, 12828.
[44] J. Lü, C. Perez-Krap, F. Trousselet, Y. Yan, N. H. Alsmail, B. Karadeniz, N. M. Jacques, W. Lewis, A. J. Blake, F. X. Coudert, R. Cao, M. Schröder, *Cryst. Growth Des.* **2018**, 18, 2555.
[45] H. Yamagishi, H. Sato, A. Hori, Y. Sato, R. Matsuda, K. Kato, T. Aida, *Science* **2018**, 361, 1242.
[46] Y. X. Li, H. Y. Yu, F. F. Xu, Q. Y. Guo, Z. G. Xie, Z. Y. Sun, *Crystengcomm* **2019**, 21, 1742.
[47] X. T. He, Y. H. Luo, D. L. Hong, F. H. Chen, Z. Y. Zheng, C. Wang, J. Y. Wang, C. Chen, B. W. Sun, *ACS Appl. Nano Mater.* **2019**, 2, 2437.
[48] Y. S. Yang, L. B. Li, R. B. Lin, Y. X. Ye, Z. Z. Yao, L. Yang, F. H. Xiang, S. M. Chen, Z. J. Zhang, S. C. Xiang, B. L. Chen, *Nat Chem* **2021**, 13, 933.
[49] Y. C. Lv, D. L. Li, A. Ren, Z. L. Xiong, Y. A. Yao, K. C. Cai, S. C. Xiang, Z. J. Zhang, Y. S. Zhao, *ACS Appl. Mater. Inter.* **2021**, 13, 28662.
[50] L. S. Gong, Y. X. Ye, Y. Liu, Y. B. Li, Z. B. Bao, S. C. Xiang, Z. J. Zhang, B. L. Chen, *ACS Appl. Mater. Inter.* **2022**, 14, 19623.
[51] L. Zhang, H. J. Li, H. Q. Zheng, Y. Yang, Y. J. Cui, D. R. Yang, G. D. Qian, *Adv. Opt. Mater.* **2023**, 11, 2202598.
[52] L. Ma, H. Arman, Y. Xie, W. Zhou, B. L. Chen, *Cryst. Growth Des.* **2022**, 22, 3808.
[53] A. Karmakar, R. Illathvalappil, B. Anothumakkool, A. Sen, P. Samanta, A. V. Desai, S. Kurungot, S. K. Ghosh, *Angew. Chem. Int. Edit.* **2016**, 55, 10667.

[54] D. W. Kang, M. Kang, H. Kim, J. H. Choe, D. W. Kim, J. R. Park, W. R. Lee, D. Moon, C. S. Hong, *Angew. Chem. Int. Edit.* **2019**, 58, 16152.
[55] Z. B. Bao, D. Y. Xie, G. G. Chang, H. Wu, L. Y. Li, W. Zhou, H. L. Wang, Z. G. Zhang, H. B. Xing, Q. W. Yang, M. J. Zaworotko, Q. L. Ren, B. L. Chen, *J. Am. Chem. Soc.* **2018**, 140, 4596.
[56] Y. Liu, J. J. Dai, Z. G. Zhang, Y. W. Yang, Q. W. Yang, Q. L. Ren, Z. B. Bao, *Chem-Asian J.* **2021**, 16, 3978.
[57] Y. Liu, J. J. Dai, L. D. Guo, Z. G. Zhang, Y. W. Yang, Q. W. Yang, Q. L. Ren, Z. B. Bao, *CCS Chem.* **2022**, 4, 381.
[58] a)Y. Liu, H. Wu, L. Guo, W. Zhou, Z. Zhang, Q. Yang, Y. Yang, Q. Ren, Z. Bao, *Angew. Chem. Int. Edit.* **2022**, 61, e202117609; b)Y. Liu, Q. Q. Xu, L. H. Chen, C. H. Song, Q. W. Yang, Z. G. Zhang, D. Lu, Y. W. Yang, Q. L. Ren, Z. B. Bao, *Nano Res.* **2022**, 15, 7695.
[59] A. A. Zhang, D. H. Si, H. B. Huang, L. Xie, Z. B. Fang, T. F. Liu, R. Cao, *Angew. Chem. Int. Edit.* **2022**, 61, e202203955.
[60] W. Liang, F. Carraro, M. B. Solomon, S. G. Bell, H. Amenitsch, C. J. Sumby, N. G. White, P. Falcaro, C. J. Doonan, *J. Am. Chem. Soc.* **2019**, 141, 14298.
[61] W. Gong, D. D. Chu, H. Jiang, X. Chen, Y. Cui, Y. Liu, *Nat. Commun.* **2019**, 10, 600.
[62] Z. Yu, X. Liu, M. Zhao, W. Zhao, J. Liu, J. Tang, H. Liao, Z. Chen, S. Zhou, *Bioresour. Technol.* **2019**, 274, 198.
[63] Y. Wang, X. D. Hou, C. Y. Liu, M. K. Albolkany, Y. Wang, N. N. Wu, C. H. Chen, B. Liu, *Nat. Commun.* **2020**, 11, 3124.
[64] A. Comotti, S. Bracco, A. Yamamoto, M. Beretta, T. Hirukawa, N. Tohnai, M. Miyata, P. Sozzani, *J. Am. Chem. Soc.* **2014**, 136, 618.
[65] A. Yamamoto, T. Hirukawa, I. Hisaki, M. Miyata, N. Tohnai, *Tetrahedron Lett* **2013**, 54, 1268.
[66] G. L. Xing, T. T. Yan, S. Das, T. Ben, S. L. Qiu, *Angew. Chem. Int. Edit.* **2018**, 57, 5345.
[67] L. Ma, Y. Xie, R. S. H. Khoo, H. Arman, B. Wang, W. Zhou, J. Zhang, R.-B. Lin, B. Chen, *Chem-Eur. J.* **2022**, 28, e202104269.
